# Intracellular phase separation of globular proteins facilitated by short cationic peptides

**DOI:** 10.1038/s41467-022-35529-2

**Published:** 2022-12-22

**Authors:** Vivian Yeong, Jou-wen Wang, Justin M. Horn, Allie C. Obermeyer

**Affiliations:** Department of Chemical Engineering, Columbia University, New York, NY 10027 USA

**Keywords:** Synthetic biology, Biomaterials - cells, Biophysical chemistry, Peptides

## Abstract

Phase separation provides intracellular organization and underlies a variety of cellular processes. These biomolecular condensates exhibit distinct physical and material properties. Current strategies for engineering condensate formation include using intrinsically disordered domains and altering protein surface charge by chemical supercharging or site-specific mutagenesis. We propose adding to this toolbox designer peptide tags that provide several potential advantages for engineering protein phase separation in bacteria. Herein, we demonstrate the use of short cationic peptide tags for sequestration of proteins of interest into bacterial condensates and provide a foundational study for their development as tools for condensate engineering. Using a panel of GFP variants, we demonstrate how cationic tag and globular domain charge contribute to intracellular phase separation in *E. coli* and observe that the tag can affect condensate disassembly at a given net charge near the phase separation boundary. We showcase the broad applicability of these tags by appending them onto enzymes and demonstrating that the sequestered enzymes remain catalytically active.

## Abstract (web summary)

Phase separation provides intracellular organisation via membraneless entities called biomolecular condensates. Here, the authors show that short, cationic peptide tags can drive biomolecular condensation of engineered proteins in E. coli through associative interactions with RNA.

## Introduction

Biomacromolecules can undergo liquid-liquid phase separation to form membraneless entities, often referred to as biomolecular condensates^1^. These condensates organize cellular contents in a manner orthogonal to traditional membrane-delimited organelles. Biomolecular condensates have been shown to regulate diverse biological processes—for instance, cell signaling^2^, stress response^3^, and genome restructuring^4^—and exhibit many distinct properties. Condensates are dynamic, can maintain internal environments that are different from the surrounding cytoplasm, and have the ability to alter the folded structure and reaction kinetics of the macromolecules that are sequestered within them^1,5–9^. Consequently, there has been significant interest in imparting condensates with complementary responsive elements and enzymatic functionality. Recent developments include the ability to form and sequester proteins inside condensates in response to temperature, light, and small molecules, giving rise to engineered compartments with applications in synthetic biology and biocatalysis^6,10–16^.

Many native proteins that form biomolecular condensates contain intrinsically disordered regions (IDRs), which have been shown to undergo homotypic and heterotypic phase separation^17–20^. The fusion of these endogenous IDRs onto non-native globular proteins has enabled the formation of biorthogonal translation hubs, light-responsive metabolic clusters, and controlled release protein depots^13–15^. In addition to endogenous IDRs, engineered disordered sequences have also been used to promote intracellular phase separation^10,11^. For instance, the fusion of artificial IDPs to split proteins promotes recruitment to the condensed phase in *E. coli*^11^. Moreover, phase behavior of these engineered IDPs can be tuned by altering the polypeptide sequence, primarily by controlling the relative aromatic and aliphatic content as well as molecular weight. While current strategies for engineering condensates take advantage of homotypic or specific biological, multivalent interactions to drive intracellular phase separation, the use of non-specific electrostatic interactions may provide a versatile alternative. Electrostatic interactions are a key driving force underlying the formation of many biomolecular condensates and have been shown to contribute to selective macromolecule partitioning in the condensed phase^5,17,19–21^. The IDRs of many proteins that natively phase separate are comprised of charged and aromatic residues^17,20^. Intermolecular interactions between these residues and other charged macromolecules in the cell are often sufficient to form a separate intracellular phase. Moreover, a number of studies have demonstrated that simply altering protein surface charge by genetic or chemical modification can also drive phase separation both in vitro and in vivo^22–25^.

**Fig. 1:**
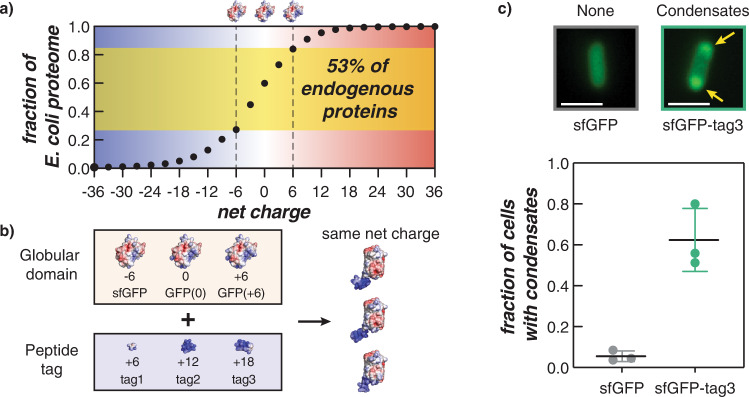
Cationic peptide tags promote the formation of biomolecular condensates in *E. coli*. **a** The cumulative distribution of overall net charge in the *E. coli* proteome reveals that the majority of proteins are not highly charged. 53% of proteins have an expected net charge between −6 and +6. **b** A schematic summarizing the GFP variants used in this study to understand how the protein globular domain and cationic peptide tags affect condensate formation in bacterial cells. Electrostatic surface maps of the globular and peptide domains as well as fusions were generated in PyMol from predicted structures. A detailed description of the method to generate these is included in the Supplementary Information. **c** Microscopy images of *E. coli* cells expressing sfGFP (left) and sfGFP-tag3 (right) at 24 h post-induction. Intracellular condensates (indicated by the yellow arrows) are observed in cells expressing supercationic proteins. Scale bar is 2 µm. Images were analyzed using MicrobeJ and a custom MATLAB script to determine the fraction of cells that form condensates for each strain, sfGFP (gray circles) and sfGFP-tag3 (green circles) (*n* > 400 cells across three biological replicates per GFP variant). Each data point corresponds to a single biological replicate with at least 130 cells analyzed. The average and standard deviation of the three biological replicates are also plotted.

Despite the success of current strategies to engineer synthetic condensates and protein sequestration, the tunability of these strategies remains a challenge and the ability to engineer multiple distinct condensates has not been established. Therefore, additional methods to promote intracellular phase separation are needed. While supercationic proteins have been demonstrated to form biomolecular condensates in vivo, global re-engineering of protein surface charge can be disruptive and difficult to execute. Very few native proteins are highly charged, and distributing charges on the protein surface requires a significant number of site-specific mutations (Fig. 1a)^26^. Consequently, the ability to supercharge a native protein by appending a small, highly charged tag offers several key advantages for promoting electrostatically-driven intracellular phase separation.

Although the use of small charge-dense tags may be advantageous for engineering protein phase separation, our understanding of how it alters the overall phase behavior of the resultant protein is limited. Several studies have investigated the role of charge anisotropy in the context of the charge patterning in polymeric systems and charge patchiness in native proteins; however, no studies have explored the effects of varying charge on both the phase separation domain and the protein globular domain^17,27–29^. A detailed examination of how both domains contribute to protein phase separation would provide additional insights for engineering biomolecular condensates in cells.

Here, we engineer intracellular condensate formation by appending to GFP short cationic peptide tags ranging from 9 to 27 amino acids in length. We investigate the interactions between these cationic peptides with various charged GFP globular domains that are representative of a majority of the *E. coli* proteome (Fig. 1a, b). We find that overall protein charge primarily governs intracellular phase separation; however, charge anisotropy may be important for tuning the persistence of condensates at a given charge threshold. We further establish the use of these cationic tags as a viable strategy for sequestering any protein of interest into bacterial condensates without the need to globally re-engineer protein surface charge or append large disordered phase separation domains.

## Results

### Designing a library of GFPs with cationic peptide tags for intracellular phase separation

To investigate the contributions of the globular domain and peptide tag on bacterial condensate formation, we constructed a library of 9 GFP tagged variants that ranged in overall charge from 0 to +24 in increments of +6 (Fig. 1b and Supplementary Fig. 1). Each variant consisted of a charge-dense peptide tag appended to the C-terminus of an isotropically charged GFP—sfGFP(−7), GFP(0), or GFP(+6). Cationic peptide tags consisted of the amino acid sequence [GGSKKRKKR]_*n*_, whereby tag1 contained only GGSKKRKKR, tag2 contained two repeats of this sequence, etc. Since sfGFP had a net charge of −7, tagged sfGFP variants contained an additional lysine reside (+1) between the C-terminus of sfGFP and the [GGSKKRKKR]_*n*_ tag in order to generate charge-equivalent variants (see the Supplementary Information). Previously reported untagged isotropic GFP variants^24,25^ were also included as controls to study the effects of overall positive charge and charge distribution on complex coacervation in *E. coli*. The charged GFP globular domains reported here are representative of 53% of the *E. coli* proteome (Fig. 1a)^26^.

Expression of GFP variants in *E. coli* was sufficient to induce condensate formation. Microscopy images acquired at 24 h post-induction depict the presence of GFP condensates in cells expressing sfGFP-tag3 and their absence in cells expressing the untagged control (Fig. 1c). Visual identification of condensates was verified by quantitative image analysis using MicrobeJ and a custom MATLAB script that calculated the fraction of condensate-containing cells detected in an image stack. Quantitative image analysis further confirmed that the majority of cells expressing the tagged GFP variant formed condensates.

### Parameters for condensate formation mediated by charge anisotropy

**Fig. 2:**
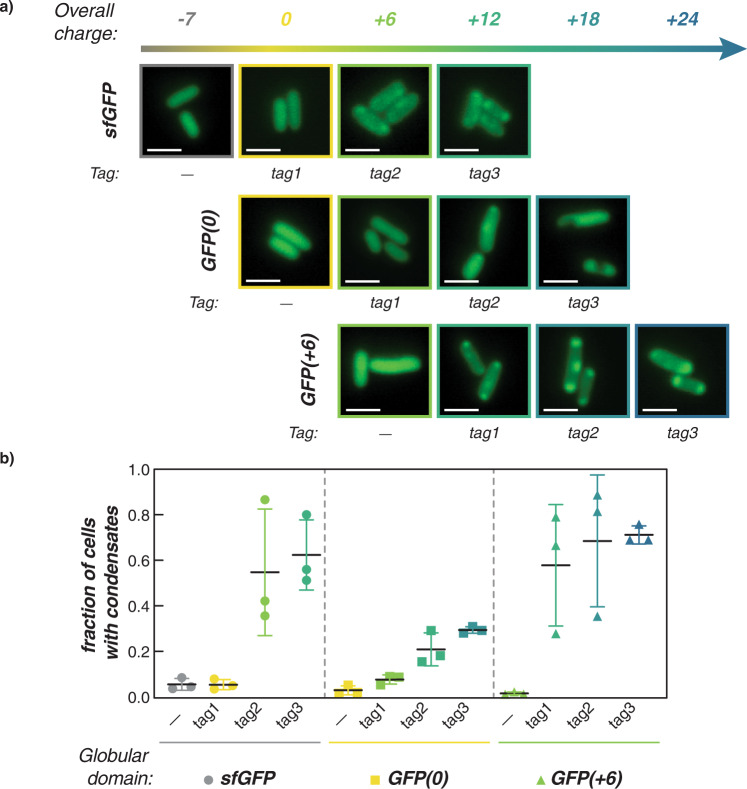
Intracellular phase separation depends on globular domain and peptide tag charge. **a** Representative microscopy images of *E. coli* cells expressing each GFP variant at 24 h post-induction (additional time points can be found in Supplementary Figs. 2–8, 10 and 11). Generally, GFP variants with an overall net charge ≥ +12 formed intracellular condensates. Additionally, sfGFP-tag2 (net charge +6) was also observed to form condensates at 24 h. Scale bar is 2 µm. **b** Analysis of *n* > 400 cells per GFP variant confirms that a net charge of ≥ +12 resulted in condensate formation. Plot depicts the fraction of cells in which condensates were detected using an automated MATLAB script. The average and standard deviation of three biological replicates are plotted for each GFP variant. Circles indicate sfGFP variants, squares indicate GFP(0) variants, and triangles indicate GFP(+6) variants; −6 net charge (gray), 0 net charge (yellow), +6 net charge (light green), +12 net charge (green), +18 net charge (teal), +24 net charge (blue).

To examine the influence of the globular protein charge on cationic tag-driven coacervation, all GFP variants were expressed in *E. coli* at 25 °C an the formation of condensates was monitored by optical microscopy at 2, 4, 6, and 24 h post-induction (Supplementary Figs. 2–5). Broadly, cells expressing GFP variants with a net charge greater than +6 formed condensates at 24 h post-induction, suggesting that overall charge had a greater effect on intracellular phase separation than charge distribution (Fig. 2a). Moreover, this net charge requirement for phase separation driven by electrostatic interactions is consistent with previous studies examining the intracellular phase separation of GFPs with isotropically-distributed surface charge^25^. Condensates were not observed in variants with a net charge ≤+6 at 24 h. However, one notable exception was sfGFP-tag2, which formed weak condensates with relatively modest enrichment of the GFP (~1.5 fold) in the condensed phase at 24 h despite having a net charge (+6) (Supplementary Figs. 6–8). This result suggests that while overall protein charge is the dominant parameter governing intracellular phase separation, charge distribution can have a small but noticeable effect, particularly near the critical point for phase separation via complex coacervation.

Quantitative image analysis confirmed results from Fig. 2a and showed that condensates were not observed 24 h post-induction in GFP strains when the net charge was <+6 (Fig. 2b and Supplementary Figs. 9 and 10). While the automated image analysis did identify some false positives, the relatively low rate (<0.11) enabled facile differentiation between strains that formed condensates and those that did not. To verify this approach, this method of analysis was also applied to previously reported isotropically-charged GFP variants as untagged controls, and revealed that cell fraction <0.11 was an appropriate cutoff for determining the presence of condensates at 8 and 24 h^25^ (Fig. 2b and Supplementary Fig. 9). This approach to image analysis also showed variance between some biological replicates, but this variance was reflective of observed differences between the replicates—with some replicates having clear condensates in nearly all of the cells and others having weak condensates in a smaller fraction of the imaged cells. Altogether, the results of this quantitative analysis fully aligned with those drawn from visual inspection. The initial differentiation of cells with and without condensates was determined using a fixed, minimal intensity threshold (1.20) to distinguish condensates from the cytoplasm, whereby the pixel intensity in identified condensate(s) was at least 20% higher than that of the cytoplasm identified at the midpoint of the cell. But, this intensity threshold could readily be varied to probe the relative partitioning of each protein variant between the condensate and cytoplasm. By varying this threshold from 1.05 to 2.00, we observed that the distribution of charge impacts the ratio of protein in the condensed and dilute phases (Supplementary Figs. 6–8). In particular, condensates formed with the GFP(0) tagged variants showed minimal enrichment of the protein in the condensed phase relative to condensates formed with the same overall net charge but a higher magnitude of charge on the globular domain (e.g., sfGFP tagged variants, GFP(+6) tagged variants, and GFP(+18)). Additionally, we found that isotropically supercharged variants consistently had an increased ratio of protein in the condensed relative to the dilute phase when compared to condensates formed via cationic tags.

### Traversing the phase boundary permits reversible condensate formation

**Fig. 3:**
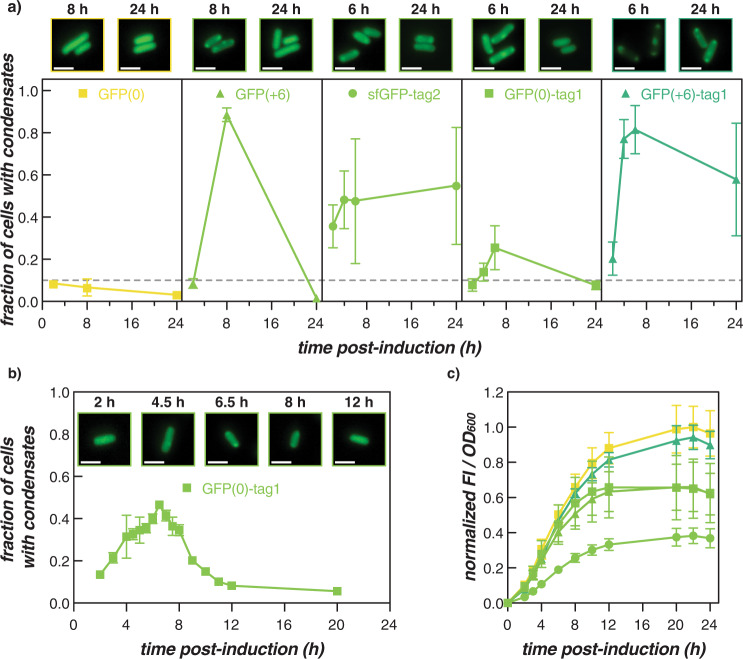
Changing GFP concentration allows for reversible intracellular phase separation. **a** The fraction of cells with condensates was monitored over time for select GFP variants. Interestingly, GFP(+6) and GFP(0)-tag1 form condensates at 6–8 h, but not at earlier or later time points, suggesting that intracellular phase separation of supercationic GFP is reversible. **b** Condensate formation and disassembly in cells expressing GFP(0)-tag1 monitored for 20 h after induction. The maximum fraction of cells with condensates was observed at 6.5 h post-induction, after which condensates fully disassembled at 12 h. Representative microscopy images of cells at the indicated time post-induction are provided (in **a** and **b**). Scale bars are 2 µm. **c** The ratio of GFP fluorescence intensity to cell density was used as a proxy for intracellular GFP concentration. Intracellular concentrations of GFP(+6) and GFP(0)-tag1 over time follow a similar trend that is distinct from the other GFP variants shown. The average and standard deviation of three biological replicates are plotted for each GFP variant. The symbols and colors used are the same as for **a**. Circles indicate sfGFP variants, squares indicate GFP(0) variants, and triangles indicate GFP(+6) variants; 0 net charge (yellow), +6 net charge (light green), +12 net charge (green). Individual biological replicates of the variants shown in parts (**a**) and (**c**) can be found in Supplementary Figs. 9, 10, and 12.

A closer analysis of intracellular condensate formation over time revealed that while the majority of cationic GFP variants formed condensates that persisted, condensate disassembly was observed for two of the variants, both of which had an overall charge of +6. As a result, all GFP variants with a net charge of +6—untagged GFP(+6), GFP(0)-tag1, and sfGFP-tag2—were further examined and the fraction of cells containing condensates was plotted as a function of time (Fig. 3a). Similar plots for all untagged and tagged variants can be found in Supplementary Fig. 11.

Reversible condensate formation has previously been observed in cells expressing GFP(+6) without a tag, whereby condensates formed at 8 h post-induction were completely disassembled by 24 h^25^. Here, we report that the charge-equivalent variant, GFP(0)-tag1, behaved similarly: condensates were first observed at early time points post-induction (4–6 h) and then fully disassembled by the final observed time point (24 h) (Fig. 3a). Upon closer monitoring, cells expressing GFP(0)-tag1 formed condensates around 4 h post-induction and exhibited the highest fraction of cells containing condensates at 6.5 h post-induction (Fig. 3b). After peaking in early stationary phase, these condensates then disappeared as protein expression continued, leaving a homogeneous distribution of protein throughout the cell (Fig. 3b, c). This finding provides additional evidence that for intracellular complex coacervation, or associative phase separation driven by electrostatic interactions, a protein net charge of +6 represents a threshold for phase separation at achievable protein concentrations in *E. coli*. We note that there are many other possible driving forces for intracellular phase separation that are unlikely to be significantly influenced by protein net charge. As a control for globular domain charge, GFP(0) was not observed to phase separate at any time point, indicating that the inclusion of one tag repeat was responsible for the temporary formation of condensates in cells expressing GFP(0)-tag1. When appended to a more cationic globular domain, tag1 facilitated condensate formation at all time points monitored (2, 4, 6, and 24 h post-induction; see GFP(+6)-tag1 in Fig. 3a). Interestingly, another charge-equivalent variant—sfGFP-tag2—formed condensates at 2 h that persisted until 24 h. Appending tag2 results in a larger increase in the charge patchiness on the protein, which may allow sfGFP-tag2 to undergo intracellular phase separation at both earlier and later time points.

We hypothesized that the reversible formation of condensates could be explained by increasing protein concentration throughout the phases of growth as phase separation is also a function of protein concentration^24,25,30^. Growth assays were performed for the five GFP variants highlighted in Fig. 3a, whereby optical density and GFP fluorescence intensity were monitored to approximate the intracellular GFP concentration over time (Fig. 3c and Supplementary Fig. 12). For all GFP variants, the ratio of GFP fluorescence to optical density increased from 2 to ~12 h post-induction and then plateaued. GFP(0) and GFP(+6)-tag1 demonstrated the highest GFP expression (Fig. 3c). In contrast, sfGFP-tag2 showed the lowest GFP expression with GFP(+6) and GFP(0)-tag1 exhibiting intermediate expression. In support of our hypothesis, GFP(+6) and GFP(0)-tag1 exhibited very similar GFP expression levels, suggesting that both charge-equivalent variants likely have similar underlying mechanisms for reversible phase separation. Since the five GFP variants exhibited different GFP concentrations at early (2 h), intermediate (6–8 h), and late (24 h) time points, we hypothesized that the mechanism for reversible condensate formation involves traversing the intracellular binodal phase boundary and that this phase boundary varies with protein charge (Supplementary Fig. 13). We demonstrated that overall protein charge plays a more dominant role than charge distribution in determining whether intracellular phase separation is observed (see Fig. 2). However, distinct differences are observed in the relative concentration of protein in the condensed and dilute phases for proteins with the same net charge but varying charge distribution. For example, amongst proteins with a net charge of +18 we observe an increased ratio of protein in the condensed phase relative to the cytoplasm as the charge on the globular domain increases, with isotropically supercharged protein having the highest enrichment of protein in the condensed phase (Supplementary Fig. 6).

Previously, we provided evidence using nucleic acid stains that RNA is a component of GFP condensates formed with an isotropically supercharged variant, which we also demonstrate here for a selection of tagged GFP variants (Supplementary Fig. 14). DAPI staining revealed that DNA was excluded from GFP(+6)-tag3 condensates. In contrast, staining with SYTO17, which non-selectively binds to DNA and RNA, demonstrated an even staining across the cell, indicating that RNA partitions into the GFP condensates whereas DNA is excluded. We had also demonstrated using isotropic GFP variants that the phase boundary broadens with increasing GFP charge when mixed with RNA in vitro^25^. We further showed that broadening of the phase boundary correlated with the propensity to form condensates in *E. coli*. In line with our previous findings, we hypothesize that GFP(+6)-tag1 exhibits a broader phase boundary and sufficient interaction strength, allowing the protein to remain phase separated despite a large change in GFP concentration during cell growth (Supplementary Fig. 13). In contrast, GFP(+6) and GFP(0)-tag1 have a lower net charge and, therefore, a smaller two-phase region in the cellular environment. This implies that the protein concentration in the cell traverses the phase boundary more than once to both form and disassemble condensates despite smaller changes in GFP concentration. Finally, the charge-equivalent variant, sfGFP-tag2, is able to maintain condensate formation. The increased interaction strength provided by the longer tag2 permits the protein to undergo intracellular phase separation at lower protein concentrations than its charge-equivalent counterparts, and the small changes in protein concentration over time are not sufficient for traversal of the binodal phase boundary, allowing the condensate to persist. These experiments emphasize that sizeable perturbations can be made at the physiological phase boundary by modest modifications to protein charge anisotropy and concentration.

### Condensates behave as GFP/RNA coacervates

**Fig. 4:**
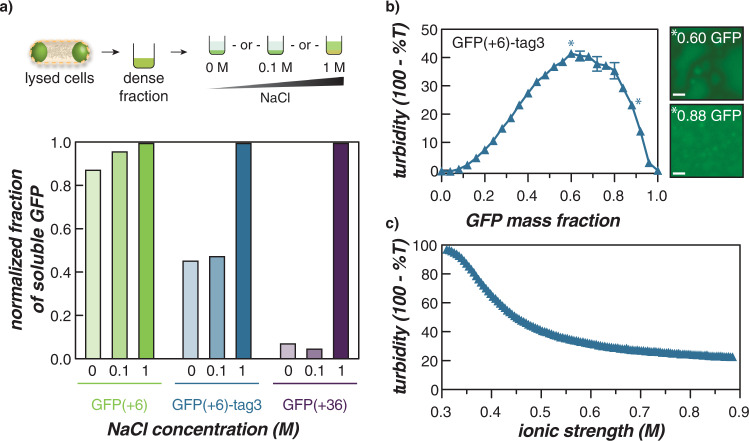
In vitro characterization of GFP(+6)-tag3/RNA coacervates. **a** The schematic (top) summarizes the solubility of GFP(+6)-tag3 intracellular condensates in buffers of increasing ionic strength. Similar to the highly supercharged, condensate-forming variant—GFP(+36)—GFP(+6)-tag3 was extracted from condensates ex vivo by increasing NaCl concentration, while a variant, GFP(+6) that did not form condensates at this time did not show preferential extraction as a function of salt concentration (bottom). Scheme adapted with permission from *ACS Cent. Sci*. **2020**, *6*(12), 2301–2310. Copyright 2020 American Chemical Society. **b** Turbidimetry measurements of GFP(+6)-tag3 with total RNA from torula yeast at 1 mg/ml total macromolecule concentration. Data points represent the average and error bars represent the standard deviation of three technical replicates. Microscopy images (right) depict the GFP channel image of the GFP/RNA mixture at 0.6 (top) or 0.88 (bottom) GFP mass fraction, which demonstrated the highest turbidity or formation of individual droplets that did not coalesce on the surface of the plate, respectively. Scale bars are 10 µm. **c** NaCl titration was conducted at 0.6 GFP mass fraction in 1 ml total volume of GFP/RNA mixture prepared from 1 mg/ml protein and RNA stocks. Both turbidity and salt stability assays were conducted in a physiological buffer that mimics the intracellular ion concentration in *E. coli* (70 mM K_2_HPO_4_, 60 mM KCl, 40 mM NaCl, pH 7.4).

While some engineered bacterial condensates demonstrated reversible formation, the condensates formed by a majority of the GFP variants persisted. As result, engineered bacterial condensates were also investigated for physical properties characteristic of complex coacervates to establish that they were formed via these engineered electrostatic interactions. To probe the physical properties of the GFP condensates, solubility assays were conducted to demonstrate that condensates were held together by electrostatic interactions. GFP(+6)-tag3 was chosen as a representative tagged condensate-forming variant because it would be the least likely to undergo condensate reversibility in cells due to its high charge density. GFP(+36) was previously shown to be extracted from condensates using a buffer with high ionic strength and was used here as a positive control^25^. GFP(+6) was used as an untagged control that did not form persistent condensates. Protein expression was induced for 24 h and the presence or absence of condensates was verified by microscopy prior to cell harvesting. All cells were then lysed and washed using a buffer without NaCl (50 mM NaH_2_PO_4_, pH 8.0), and then resuspended in buffers with varying NaCl concentration (0, 0.1, or 1 M) (Fig. 4a). The fraction of GFP extracted from condensates was calculated by comparing the ratio of GFP relative to the total protein found in the soluble fraction as determined by SDS-PAGE analysis (Supplementary Fig. 15).

As expected, GFP(+6) showed no significant preference for extraction with different NaCl concentrations as it was soluble at the time cells were harvested (Fig. 4a). In contrast, the two condensate-forming variants—GFP(+6)-tag3 and GFP(+36)—preferred extraction in buffer supplemented with the highest NaCl concentration (1 M). Notably, a larger fraction of GFP(+6)-tag3 was extracted at lower NaCl concentrations when compared to GFP(+36). This can be explained by differences in overall net charge; since GFP(+6)-tag3 has a lower net charge (+24), its extraction requires less salt and, therefore, becomes more soluble at lower NaCl concentrations. Nonetheless, increased extraction of GFP(+6)-tag3 from condensates at higher ionic strength suggests the key role of electrostatics in the formation of condensates, which is consistent with the formation of intracellular complex coacervates.

To provide further evidence that condensates are formed through electrostatic interactions, we investigated the ability of GFP(+6)-tag3 to undergo phase separation in vitro with RNA, the most abundant anionic biopolymer in the cell by weight^31^. Purified GFP(+6)-tag3 was mixed with total RNA from torula yeast at a constant total macromolecule concentration in a buffer (70 mM K_2_HPO_4_, 60 mM KCl, 40 mM NaCl, pH 7.4) that mimicked intracellular ion concentrations and pH (Fig. 4b). Phase separation was observed across a wide range of mixing ratios under physiological conditions and a modest total macromolecule concentration. Moreover, the GFP/RNA mixture formed liquid-like coacervates that coalesced even at the mixing ratio corresponding to the highest turbidity (Fig. 4b and Supplementary Fig. 16). A titration of NaCl was also performed to determine the critical salt concentration, which aligned with the results of the solubility assay, with the coacervate phase dissolving at an ionic strength of ~0.4 M (Fig. 4c). We note the discrepancy in the maximum turbidity value between the turbidity (Fig. 4b) and salt stability (Fig. 4c) assays was due to differences in the pathlength of the vessels in which measurements were obtained. Taken together, these in vitro experiments provide supporting evidence for the complex coacervate-like nature of the engineered condensates formed from cationically tagged proteins in bacteria.

### Sequestration of enzymes in engineered bacterial condensates

**Fig. 5:**
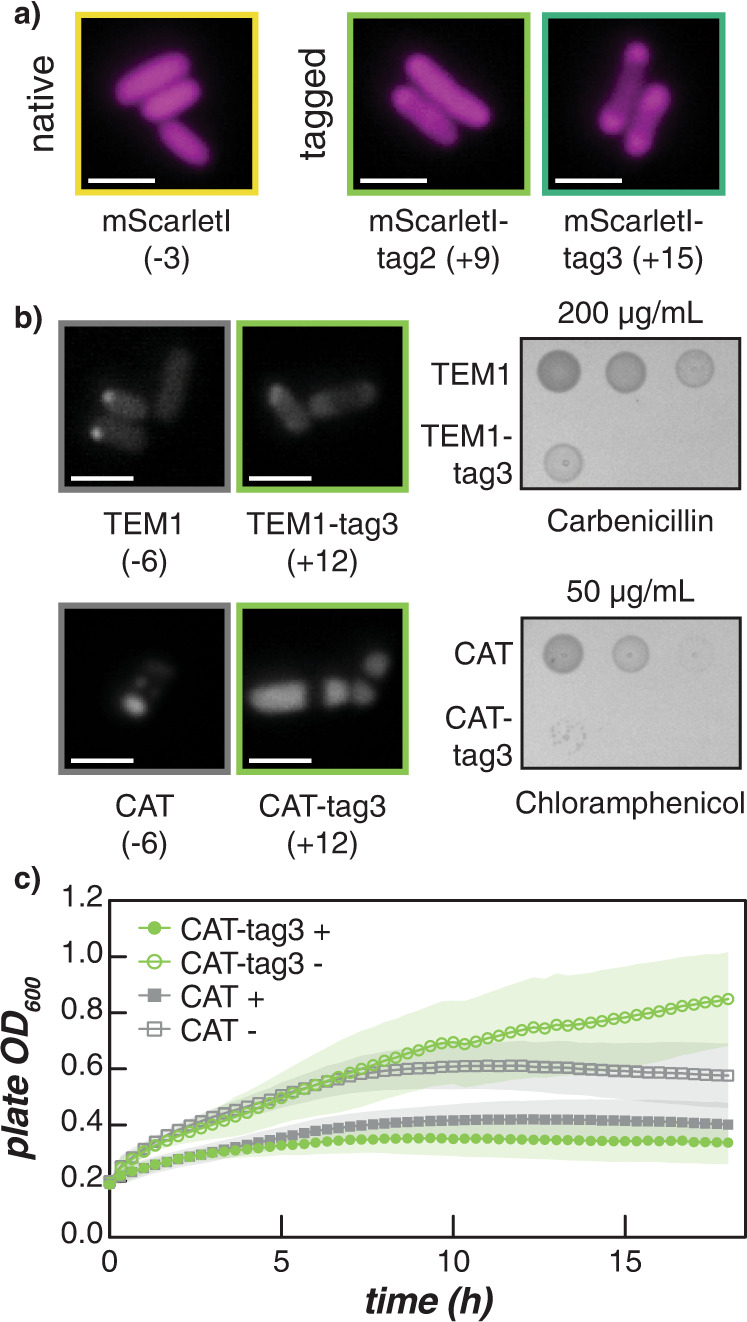
Cationic tags induce intracellular phase separation of proteins of interest. **a** Microscopy images of mScarletI and tagged variants at 24 h post-induction. **b** Microscopy images of β-lactamase (TEM1), chloramphenicol acetyltransferase (CAT), and tagged derivatives at 24 h post-induction. Each protein contained an N-terminal tetracysteine tag for fluorescence staining (left). Scale bars on all micrographs are 2 µm. Spotting assay on LB plates supplemented with the respective antibiotic indicate that the presence of the cationic tag does not completely inhibit growth (right). Reduced fitness of the tagged relative to the untagged strains was also observed on control plates with no antibiotic (Supplementary Fig. 26). **c** The growth of CAT (squares) and CAT-tag3 (circles) in liquid culture was monitored in the presence (+; filled symbols) or absence (−; open symbols) of chloramphenicol. Treatment with antibiotic resulted in modest growth defects, but no differences were observed between the CAT variants. Data points represent the average of five biological replicates and shaded regions show the standard deviation.

We then wanted to demonstrate the utility of these cationic tags by examining how they affected the phase separation of a diverse set of proteins. Tags with two or three repeats were appended to mScarletI, a red fluorescent protein that has an expected charge of −3. Figure 5a depicts representative microscopy images of cells expressing the untagged and tagged mScarletI variants at 24 h post-induction. Addition of either tag was sufficient to drive condensate formation in cells. Quantitative image analysis revealed that while both tagged mScarletI variants remain phase separated at all time points, condensates become significantly less distinct for mScarletI-tag2 at 24 h (Supplementary Figs. 17–20).

To further validate the ability of cationic tags to promote the formation of biomolecular condensates, cationic tags were appended to enzymes that confer antibiotic resistance, TEM1 (β-lactamase) and CAT (chloramphenicol acetyltransferase) which degrade carbenicillin and chloramphenicol, respectively. Both native enzymes have an expected charge of −6, which is in line with the charge of the globular domains investigated using GFP variants. In addition to the cationic tag, a N-terminal tetracysteine (TC) tag was added to these proteins to enable visualization via fluorescence microscopy with FlAsH-EDT_2_ or ReAsH-EDT_2_. Staining unexpectedly revealed local regions of high protein concentration in cells expressing untagged TEM1 and CAT at 24 h post-induction; however, appending tag3 resulted in the formation of condensates that were similar in size to those formed with tagged fluorescent proteins (Fig. 5b and Supplementary Fig. 21). Moreover, the condensates observed were similar in size and morphology to sfGFP-tag3 condensates that were stained with ReAsH-EDT_2_, indicating that CAT-tag3 and TEM1-tag3 condensates were not an artifact of the staining procedure used (Supplementary Fig. 22). While the untagged enzymes formed puncta, closer inspection of their size distinguished them from those formed with the cationic tagged variants (Supplementary Fig. 23). Furthermore, we evaluated the cellular localization of DNA, via DAPI staining, for CAT variants lacking the TC tag (Supplementary Fig. 24). Cells expressing CAT-tag3 show compaction of the nucleoid, consistent with the exclusion of DNA from the cellular condensates. In contrast, cells expressing CAT without any tags showed uniform distribution of DAPI signal throughout the length of the cell. This combined evidence supports the role of cationic tags promoting complex coacervation of various proteins of interest inside cells.

Growth assays were also conducted to test the ability of untagged and tagged strains to survive on LB plates supplemented with the respective antibiotic and inducer (Supplementary Fig. 25). Both TEM1-tag3 and CAT-tag3 were able to grow on respective antibiotic plates (Fig. 5b). However, a clear reduction in fitness was observed in the absence of antibiotic (this was also observed for a sfGFP-tag3 control; Supplementary Fig. 26). In addition to arabinose, TEM1 variants were also grown on antibiotic plates supplemented with glucose, which reduces expression below uninduced levels. When grown on glucose plates without antibiotic, minimal growth differences were observed between the TEM1 variants, suggesting that condensate formation is likely responsible for the reduction in fitness on solid media. In spite of this, TEM1-tag3 maintained growth across all carbenicillin concentrations tested. CAT-tag3 also grew on 25 µg/ml and 50 µg/ml chloramphenicol plates but appeared to be more sensitive to higher concentrations of chloramphenicol. However, these differences in antibiotic resistance for CAT-tag3 and TEM1-tag3 variants cannot be directly compared due to variations in experimental setup (different inducible constructs were used to express the CAT and TEM1 variants resulting in varying levels of protein expression) and recommended antibiotic concentrations^32^ (see Supplementary Fig. 25). In vitro studies of purified CAT and CAT-tag3 demonstrated that both proteins maintain activity, indicating that the peptide tag does not directly compromise enzymatic activity (Supplementary Fig. 27). Although a reduction in fitness when grown on solid media was observed for all tagged variants, incorporation of the cationic peptide tag allowed for increased sequestration of the enzyme into bacterial condensates without abolishing enzymatic activity.

Moreover, growth in liquid media showed noticeably different trends (Fig. 5c, Supplementary Fig. 28). Cells expressing sfGFP or CAT, with and without cationic tags, were grown in a 96-well microtiter plate and the optical density was monitored on a plate reader for 18 h. Similar growth was observed for CAT and CAT-tag3 treated with 200 µg/ml chloramphenicol regardless of whether protein expression was induced with IPTG or not. Interestingly, in the absence of chloramphenicol, no difference was observed between the induced and uninduced CAT-tag3 samples whereas CAT overexpression appeared to impair cell growth. sfGFP and sfGFP-tag3 were also included as controls. As expected, sfGFP variants did not grow when treated with chloramphenicol. In the absence of chloramphenicol, sfGFP and sfGFP-tag3 overexpression also resulted in decreased cell growth. These findings suggest that while cationic tags significantly perturb growth on solid media, when grown in liquid culture the formation of condensates via cationic tags is well tolerated.

To assess growth differences between isotropic and tagged GFP variants of equivalent charge, a spotting assay was performed on agar plates with or without IPTG (Supplementary Fig. 29). Briefly, cultures were normalized to the same OD_600_ and a 5-fold serial dilution was performed in a 96-well plate. Samples were then transferred to LB agar plates using a 96-pin replicator and grown at 25 °C for ~30 h. For both isotropic and tagged GFP variants, cell growth was reduced upon overexpression of GFP variants with increasing charge (Supplementary Fig. 29). However, tagged variants demonstrated better growth than their isotropic equivalents, indicating that peptide tags may help minimize fitness reductions associated with increasing protein charge. As a control, all GFP variants appeared to grow similarly in the absence of inducer, indicating that observed growth differences are likely due to protein overexpression and condensate formation (Supplementary Fig. 29).

## Discussion

Herein, we report the ability of short cationic peptides to promote the formation of complex coacervate-based biomolecular condensates in *E. coli*. In this study, cationic tags of varying lengths were appended onto globular domains in order to investigate how the net charge of these individual components act in concert to affect intracellular phase separation. The expected charge on globular domains tested ranged from −6 to +6, which spans the expected charge of 53% of the *E. coli* proteome^26^ (Fig. 1a, b). The use of tags, 9–27 amino acids long, described here provides a simple and versatile approach to engineer protein phase separation in cells. We show that the shortest tag is able to promote condensate reversibility at the physiological phase boundary and that near the physiological phase boundary, tag length can determine condensate behavior (Fig. 3 and Supplementary Figs. 10–13).

Our results indicated that overall charge was the main parameter governing whether or not any condensates would form. We observed condensate formation via complex coacervation as GFP variants with net charge >+6 generally phase separated regardless of the identity of the globular domain or tag (Fig. 2 and Supplementary Figs. 2–11). We note here that the persistence of condensates at 24 h post-induction cannot be decoupled from phase separation triggered by cellular stress (observed in eukaryotic systems^3,33,34^) as the cell is in the stationary phase at this point. Many of these variants exhibited condensate formation well before stationary phase (for instance, at 2 or 4 h post-induction) indicating that at these early time points, condensate formation is likely due to associative phase separation of cationic GFP with endogenous anionic biomacromolecules. Even at high intracellular protein concentrations, GFP variants did not form condensates if they were not sufficiently charged. This is illustrated by the fact that GFP(+6)-tag1 formed condensates while GFP(0) did not even though both proteins exhibited similar intracellular protein concentrations (Fig. 3 and Supplementary Figs. 11 and 12). Moreover, we investigated the physical properties of condensates and verified in vitro that, like complex coacervates, they are held together by electrostatic interactions and are sensitive to the ionic strength of the solution (Fig. 4 and Supplementary Figs. 15 and 16).

While overall charge had a larger effect on condensate formation than charge distribution, charge distribution did have significant effects on the partitioning of the tagged protein in the condensed phase. This impact on phase separation became markedly pronounced near the phase separation threshold. Closer examination of GFP variants with a net charge of +6 revealed that the variants with increased charge patchiness (in particular, sfGFP-tag2) maintained condensates over time while the lower charge density variants formed condensates that disassembled at later time points (Fig. 3 and Supplementary Fig. 10). Various studies in protein and polypeptide systems have reported that increasing the charge anisotropy provides broader phase boundaries^27,29,35,36^, which we hypothesize allows sfGFP-tag2 to remain phase separated even as protein concentration and cytoplasmic interaction strength fluctuates (Supplementary Figs. 11–13). In contrast, lower charge density variants with a net charge of +6 have an anticipated decreased interaction strength and are closer to the critical point. As a result, these variants are able to traverse this phase boundary in response to changes in the cytoplasmic environment. In addition to these differences near the phase boundary, we also noted that condensates formed with a net neutral globular domain (GFP(0)) were quite different than those formed with globular domains with higher net charge. For example, the GFP(0) condensates consistently had lower partitioning of the GFP into the condensed phase, resulting in a lower overall fraction of cells with condensates.

Cationic peptide tags are useful tools for bioengineering and biological study given the dynamic nature of condensates, the ability to design condensate reversibility or persistence, and the ease of altering a protein’s overall charge density. The work reported here provides a foundation for the development of these cationic tags as potential tools to control condensate formation in bacteria. The primary sequence of a globular protein can be used to predict the tag length/charge required for intracellular phase separation. After calculating the net charge of the globular protein of interest, C-terminal cationic tags should be appended such that the engineered protein has a net charge of +6 if transient condensate formation is desired or a net charge >+6 for persistent condensates. We have demonstrated that appending these designer tags onto enzymes can result in their sequestration in condensates (Fig. 5 and Supplementary Figs. 21 and 22) and engineered enzymes within the coacervate-like condensate retain their activity, highlighting the potential utility of these tags (Supplementary Fig. 26). Simultaneously, however, this approach may present a few limitations that require further characterization and improvement. Most critically, additional studies are needed to probe the impact of condensate formation on cell viability and methods to minimize growth defects are necessary in order to fully harness the utility of these cationic tags. The effects of these tags on very large proteins and recombinant proteins that have a tendency to aggregate are unknown. Additionally, we are limited by the protein expression system to a certain intracellular protein concentration regime. Enhanced control of protein expression would permit more precise delineation of the intracellular phase boundary. In addition, varying the identity of charged amino acids and non-charged residues (e.g., hydrophilic, hydrophobic, or aromatic) in peptide tags may allow for further fine-tuning of condensate formation and dynamics at or near the intracellular phase boundary. Striking an intricate balance between overall protein charge and charge anisotropy may allow for improved control of engineered condensate formation and disassembly. With this initial demonstration, we have expanded the groundwork for rational, charged-based engineering of condensate formation and protein sequestration. As an extension of this work, we envision using cationic peptide tags to recruit multiple proteins to intracellular condensates and build complex, functional organelles by incorporating enzymes involved in biochemical pathways.

## Methods

### Cloning

Three different cationic tags (tag1, tag2, and tag3) were created by modifying the number of repeats of the amino acid sequence, GGSKKRKKR. These tags were then appended to each of the isotropic GFP (sfGFP(−7), GFP(0), and GFP(+6)) variants using a combination of PCR, restriction enzyme digestion, and T4 ligation. Primer sequences, plasmid templates, and restriction sites used can be found in the Supplementary Information along with details for cloning of mScarletI, CAT, and TEM1 variants. All GFP and mScarletI variants contained a N-terminal 6xHis tag unless specified otherwise.

### Protein expression

Glycerol stocks of NiCo21(DE3) cells (New England Biolabs) transformed with each GFP variant were streaked onto LB agar plates with added ampicillin (100 µg/ml, Gold Biotechnology). A single colony was inoculated into sterilized LB media supplemented with ampicillin (100 µg/ml) and grown in an incubator (Thermo Scientific MaxQ 6000) overnight at 37 °C with shaking at 225 rpm. The overnight cultures were back diluted to OD_600_ ~0.1 in 25 ml LB media supplemented with ampicillin (100 µg/ml) in sterile 125 ml Erlenmeyer flasks. Cultures were grown at 37 °C, shaking at 225 rpm for 2–3 h. At OD_600_ = 0.8–1.0, cultures were induced with 1 mM isopropyl β-d−1-thiogalactopyranoside (IPTG, Gold Biotechnology) and then kept at 25 °C with shaking at 225 rpm for 24 h. In total, 20 µl aliquots were taken from the cultures at 0, 2, 4, 6, and 24 h after induction for imaging via optical microscopy. Cells were only induced with IPTG at OD_600_ = 0.8–1.0 and additional IPTG was not added after.

### Optical microscopy

Cell samples were applied to agarose pads for imaging. The agarose pads were made by preparing 1 w/v% agarose (TopVision) in milliQ water. In total, 50 µl of melted agarose was then pipetted onto a 25 mm × 75 mm microscope slide and immediately covered by a 18 mm × 18 mm coverslip (Thermo Scientific). After the agarose solidified, the coverslip was slowly removed and then 1.5 µl of cell culture was added on top of the agarose pad. The agarose pad with sample was gently covered with the coverslip and sealed on all four sides with clear nail polish. Cells were imaged on agarose pads at 0, 2, 4, 6, and 24 h post-induction. Images were taken using a ×100 oil 1.40 NA UPlanSAPO objective (Olympus) with illumination by GFP (*λ*_*ex*_ = 479–522 nm; *λ*_*em*_ = 525–550 nm; EVOS GFP light cube) or Texas Red (*λ*_*ex*_ = 585–629 nm; *λ*_*em*_ = 628–632 nm; EVOS Texas Red light cube) and brightfield channels on an EVOS FL Auto 2 inverted fluorescent microscope. In total, 10–16 images were taken for each sample in order to acquire a sufficient number of cells per strain (>400 across three biological replicates). Figures 3 and 5, and fluorescent images in the Supplementary Information depict raw images that were background subtracted using the rolling ball algorithm with a 100 pixel radius and pseudocolored either green (cells expressing GFP or stained with FlAsH-EDT_2_) or magenta (cells expressing mScarletI or stained with ReAsH-EDT_2_) using the standard LUT in ImageJ. Brightness was manually adjusted for merged and brightfield images in the Supplementary Information for visual clarity.

### Image analysis

Raw microscopy images from the GFP or Texas Red channels were pre-processed using ImageJ with a plug-in, MicrobeJ (version 15.3 l (14)). Briefly, images were compiled into stacks and were subjected to a rolling ball background subtraction with a 100 pixel radius. Stacks of images were individually thresholded using the Li method in MicrobeJ and cells that met specific area, length, width, circularity, and angularity constraints were identified and considered for analysis. Images of individual cells identified by MicrobeJ were loaded into MATLAB for processing and identification of condensates. A custom MATLAB script was used to identify condensates and determine the fraction of condensate-containing cells. The fraction of cells containing condensates was then plotted in GraphPad Prism (version 9.1.0). The specific constraints used and complete MATLAB code can be found in the Supplementary Information.

### Chloramphenicol sensitivity spotting assay

Overnight cultures of TC-CAT, TC-CAT-tag3, TC-sfGFP, and TC-sfGFP-tag3 were prepared as described above and then diluted to OD_600_ = 0.1 in LB media supplemented with 100 µg/ml ampicillin and 10 µM FlAsH-EDT_2_ (Fisher Scientific). Cultures were grown at 37 °C with shaking at 225 rpm until they reached OD_600_ = 0.8–1, at which point cultures were induced with 1 mM IPTG and allowed to grow at 25 °C with shaking for 2 h. Subsequently, all cultures optical densities were normalized to OD_600_ = 1 in LB media supplemented with 100 µg/ml ampicillin. Normalized density cultures were diluted 100-fold into LB media. From this dilution, cultures were serially diluted 20-fold in LB media. In total, 5 µl of all normalized cultures and subsequent dilutions (100X and 2000X) were then spotted onto LB agar plates supplemented with 1 mM IPTG, 100 µg/ml ampicillin, and varying concentrations of chloramphenicol (0, 25, 50, 200, or 300 µg/ml). Samples on plates were allowed to dry fully before incubation in the dark at 25 °C for ~30 h. Plates were then imaged on a Gel Doc XR + System (Bio-Rad). Colonies from the plates were also picked and diluted in 20 µl LB media and imaged on a microscope slide to confirm the presence of condensates (as described in “Optical microscopy”). Spotting assays were similarly performed for TEM1 variants and additional details about these assays can be found in the Supplementary Information.

### Reporting summary

Further information on research design is available in the Nature Portfolio Reporting Summary linked to this article.

## Supplementary information

Supplementary Information

Reporting Summary

## Data Availability

The following data from public repositories was used herein: superfolder GFP crystal structure, PDB ID: 2B3P, and *E. coli* proteome information, UniProt: UP000002032. Raw uncropped microscopy and gel images are available via figshare 10.6084/m9.figshare.21568371. New plasmids reported here are available from Addgene, Article ID: 28233333. All other data and biological materials are available from the corresponding author upon request.

## Code availability

The MATLAB code used to identify condensate-containing cells can be found in the Supplementary Information.
