# Supplementary material for: Intracellular phase separation of globular proteins facilitated by short cationic peptides
Source: Nat Commun. 2022 Dec 22;13:7882. doi: 10.1038/s41467-022-35529-2 (PMC9780332; doi:10.1038/s41467-022-35529-2)
Supplement: Supplementary file 1 — Supplementary Information [file 41467_2022_35529_MOESM1_ESM.pdf]

Supplementary Information for:

Intracellular phase separation of globular proteins facilitated by short cationic peptides

Vivian Yeong, Jou-Wen Wang, Justin M. Horn, and Allie C. Obermeyer

Table of Contents

Supplementary Methods 1
Cloning 1
Electrostatic maps of GFP variants 8
Constraints for image analysis using MicrobeJ 8
Image analysis using a custom MATLAB script 8
Cell growth assay 8
Solubility assay 9
Protein purification for *in vitro* tests 9
MALDI-TOF mass spectrometry 9
In vitro turbidity assays and microscopy of coacervates 10
NaCl titration to determine critical salt concentration 10
FlAsH staining of TC-AmpR and -AmpR-tag3 cultures 10
ReAsH-EDT staining of TC-CAT, TC-CAT-tag3, TC-sfGFP, and TC-sfGFP-tag3 cultures 10
Carbenicillin Sensitivity Spotting Assay 11
Nucleic acid staining of cells with DAPI or SYTO17 11
Spotting assay and analysis 11
CAT and CAT-tag3 purification 11
Enzyme activity assay 12

Supplementary Figures 13
1. Electrostatic maps of GFP variants 13
2. Representative microscopy images of tagged GFP, 2 h post-induction 14
3. Representative microscopy images of tagged GFP, 4 h post-induction 16
4. Representative microscopy images of tagged GFP, 6 h post-induction 18
5. Representative microscopy images of tagged GFP, 24 h post-induction 20
6. Fraction of cells with condensates as a function of threshold, 24 h post-induction 22
7. Relative GFP localization in cells, 24 h post induction 23
8. Relative GFP localization in cells over time 24
9. Fraction of cells with condensates, isotropic GFPs 25
10. Fraction of cells with condensates, GFP with cationic tags 26
11. Reversible condensate formation 27
12. Cell growth and protein expression 28
13. Schematic of traversing the physiological phase boundary 29
14. Nucleic acid staining of cells expressing tagged GFP variants, 24 h post-induction 30
15. Solubility assay, SDS-PAGE gels 31
16. In vitro coacervation of RNA with GFP(+6)-tag3 32
17. Representative microscopy images of tagged mScarletI, 2 h post-induction 33
18. Representative microscopy images of tagged mScarletI, 4 h post-induction 34
19. Representative microscopy images of tagged mScarletI, 24 h post-induction 35
20. Fraction of cells with condensates, mScarletI with cationic tags 36
21. Representative microscopy images of TC-tagged enzymes, 24 h post-induction 37
22. Representative microscopy images of TC-tagged GFPs, 24 h post-induction 38

23. ReAsH-EDT2 and FlAsH-EDT2 staining of cells, 24 h post-induction ………………………………… 39
24. DAPI staining of cells expressing CAT, CAT-tag3, and sfGFP-tag3, 24 h post-induction ……………… 40
25. Experimental design of antibiotic sensitivity assays ……………………………………………………….. 41
26. Plate images for antibiotic sensitivity assays …………………………………………………………… 42
27. Evaluation of CAT activity *in vitro* ………………………………………………………………………... 43
28. Growth of isotropic and tagged variants in liquid media ……………………………………………… 44
29. Differential growth of isotropic and tagged sfGFP variants on solid media ……………………………… 45

Supplementary Notes

MATLAB script for identification of condensate-containing cells ………………………………………… 46

Supplementary References ……………………………………………………………………………….... 49

Supplementary Methods

Cloning

The primers used to clone positive tags onto the C-terminus of GFP were purchased from Integrated DNA technologies (IDT). Primer sequences (denoted 5' to 3') and template plasmids used are shown below. Restriction enzyme sites were engineered into the primer sequence, with the forward primer containing a NcoI cut site and the reverse primers containing a XhoI cut site. All GFP variants contained a N-terminal 6xHis tag.

The following was used as the forward primer for all GFP variants: GATATACCATGGGTCATCACCACCACC

For the cloning of GFP variants, all reverse primers were designed to contain the following sites:
XhoI cut site STOP (Tag)Tag Plasmid Binding

pVY-1: sfGFP-tag1
GFP Template: sfGFP plasmid
Reverse Primer: TAATCTCGAGTTATCTTTTCTTCCTTTTCTTTGAACCGCCTTTCTTGTACAGCTCGTC-CA

pVY-2: sfGFP-tag2
GFP Template: sfGFP plasmid
Reverse Primer: TAATCTCGAGTTATCTTTTCTTCCTTTTCTTTGAACCGCCTCTTTTCTTCCTTTTCTTT-GAACCGCCTTTCTTGTACAGCTCGTCCATTC

pVY-3: sfGFP-tag3
GFP Template: pVY-1
Reverse Primer:
ATACTCGAGTTATTAACGCTTCTTACGCTTTTTGCTACCGCCACGCTTCTTACGCTTTTTGCTACCG-CCTCTTTTCTTCCTTTTCTTTGAA

pVY-4: GFP(0)-tag1
GFP Template: GFP(0) plasmid
Reverse Primer:
ATACTCGAGTTATTAACGCTTCTTACGCTTTTTGCTACCGCCCTTGTAGCGTTCGTCCATTC

pVY-5: GFP(0)-tag2
GFP Template: GFP(0) plasmid
Reverse Primer: ATACTCGAGCTATTAACGCTTCTTACGCTTTTTGCTACCGCCACGCTTCTTGC-GTTTCTTGCTTCCTCCCTTGTAGCGTTCGTCCATTC

pVY-6: GFP(0)-tag3
GFP template: pVY-5
Reverse Primer: ATACTCGAGTTATTATCGCTTTTTACGTTTCTTTGAACCTCCACGCTTCTTAC-GCTTTTTGCTACCGCCACGCTT

pVY-7: GFP(+6)-tag1
GFP template: GFP(+6) plasmid
Reverse Primer: ATACTCGAGTTATTAACGCTTCTTACGCTTTTTGCTACCGCCCTTGTAGCGTTCGTC-CATTC (same as pVY-4 reverse primer)

VY-8: GFP(+6)-tag2

GFP template: GFP(+6) plasmid

Reverse Primer: ATACTCGAGCTATTAACGCTTCTTACGCTTTTGCTACCGCCACGCTTCTTGCGTTTCTTGCTTCCTCCCTTGTAGCGTTCGTCCATTC (same as pVY-5 reverse primer)

pVY-9: GFP(+6)-tag3

Template: pVY-8

Reverse Primer: ATACTCGAGTTATTATCGCTTTTACGTTTCTTTGAACCTCCACGCTTCTTACGCTTTTGCTACCGCCACGCTT (same as pVY-6 reverse primer)

Primers were diluted to 10 µM in Milli-Q water. PCR reactions were performed using Phusion polymerase as instructed by New England Biolabs (NEB). PCR products were purified using the QIAquick PCR purification kit (Qiagen). Purified PCR products were digested with restriction enzymes, NcoI and XhoI. DpnI was added to the digestion of PCR amplified GFP inserts but not to the digestion of the vector. Reactions were run on an 1% agarose gel (TopVision agarose) and relevant bands were then excised and purified using a QIAquick gel extraction kit (Qiagen). The final plasmid was assembled using T4 ligase (NEB) using a 5:1 molar ratio of insert to vector. 2 µL of the ligation reaction was transformed into chemically competent NEB5α cells. Following sequence verification by Genewiz, plasmids were transformed into NiCo21(DE3) cells (NEB) for protein expression and *in vivo* assays.

For the following plasmids, mScarletI-tag was amplified from DNA sequences purchased from TWIST biosciences. Vector templates and amplified PCR products were then digested using restriction enzymes and loaded on a 1% agarose gel (TopVision agarose). The relevant bands were then excised and purified as described above. Plasmids were assembled using T4 ligase at a 3:1 molar ratio of insert to vector, transformed into NEB5α and NiCo21(DE3) cells, and sequence verified.

pVY-10: mScarletI-tag2

mScarletI-tag2 TWIST template: GAAGTGCCATTCCGCCTGACCTATAAGGCTCGTATGATATATTCAGGGAGACCACAACGGTTTCCCTCTACAAATAATTTTGTTTAACTTTCTAGATTTAAGAAGGAGATATACATATGGGTCATCACCACCACCATCACGGTGGCGCTAGTAAAGGAGAAGCTGTGATTAAAGAGTTCATGCGCTTCAAAGTTCACATGGAGGGTTCTATGAACGGTCACGAGTTCGAGATCGAAGGCGAAGGCGAGGGCCGTCCGTATGAAGGCACCCAGACCGCCAAACTGAAAGTGACTAAAGGCGGCCCGCTGCCTTTTTCCTGGGACATCCTGAGCCCGCAATTTATGTACGGTTCTAGGGCGTTCATCAAACACCCAGCGGATATCCCGGACTATTATAAGCAGTCTTTTCCGGAAGGTTTCAAGTGGGAACGCGTAATGAATTTTGAAGATGGTGGTGCCGTGACCGTCACTCAGGACACCTCCCTGGAGGATGGCACCCTGATCTATAAAGTTAAACTGCGTGGTACTAATTTTCCACCTGATGGCCCGGTGATGCAGAAAAAGACGATGGGTTGGGAGGCGTCTACCGAACGCTTGTATCCGGAAGATGGTGTGCTGAAAGGCGACATTAAAATGGCCCTGCGCCTGAAAGATGGCGGCCGCTATCTGGCTGACTTCAAAACCACGTACAAAGCCAAGAAACCTGTGCAGATGCCTGGCGCGTACAATGTGGACCGCAAACTGGACATCACCTCTCATAATGAAGATTATACGGTGGTAGAGCAATATGAGCGCTCCGAGGGTCGTCATTCTACCGGTGGCATGGATGAACTATACAAAGGAGGAAGCAAGAAACGCAAGAAGCGTGGCGGTAGCAAAAAGCGTAAGAAGCGTTAATAAACTTAATTAAGGATCCGAATTCGAGCTCCGTCGACAAGCTTGCGGCCGCACTCGAGCACCTGCAGGCATGCAAGCTCTAGAGGCAAGGCTAGGTGGAGGCTCAGTG

Forward Primer: ATAAGGCTCGTATGATATATTCAGG

Reverse Primer: TGCCTCTAGAGCTTGCAT

Vector Template: pET-24a-mScarletI-v2 (plasmid map can be provided upon request)

Restriction Enzymes: NdeI and XhoI

pVY-11: mScarletI-tag3
mScarletI-tag3 TWIST template: GAAGTGCCATTCCGCCTGACCTATAAGGCTCGTATGATATATTCAG-
GGAGACCACAACGGTTTCCCTCTACAAATAATTTTGTTTAACTTTCTAGATTTAAGAAGGAGA-
TATACATATGGGTCATCACCACCACCATCACGGTGGCGCTAGTAAAGGAGAAGCTGTGATTAAA-
GAGTTCATGCGCTTCAAAGTTCACATGGAGGGTTCTATGAACGGTCACGAGTTCGAGATCGAAGG-
CGAAGGCGAGGGCCGTCCGTATGAAGGCACCCAGACCGCCAAACTGAAAGTGACTAAAGGCGGC-
CCGCTGCCTTTTTCCTGGGACATCCTGAGCCCGCAATTTATGTACGGTTCTAGGGCGTTCATCAAA-
CACCCAGCGGATATCCCGGACTATTATAAGCAGTCTTTTCCGGAAGGTTTCAAGTGGGAACGCGTA-
ATGAATTTTGAAGATGGTGGTGCCGTGACCGTCACTCAGGACACCTCCCTGGAGGATGGCACCCT-
GATCTATAAAGTTAAACTGCGTGGTACTAATTTTCCACCTGATGGCCCGGTGATGCAGAAAAAGAC-
GATGGGTTGGGAGGCGTCTACCGAACGCTTGTATCCGGAAGATGGTGTGCTGAAAGGCGACATTA-
AAATGGCCCTGCGCCTGAAAGATGGCGGCCGCTATCTGGCTGACTTCAAAACCACGTACAAAGC-
CAAGAAACCTGTGCAGATGCCTGGCGCGTACAATGTGGACCGCAAACTGGACATCACCTCTCATA-
ATGAAGATTATACGGTGGTAGAGCAATATGAGCGCTCCGAGGGTCGTCATTCTACCGGTGGCAT-
GGATGAACTATACAAAGGAGGAAGCAAGAAACGCAAGAAGCGTGGCGGTAGCAAAAAGCGTA-
AGAAGCGTGGAGGTTCAAAGAAACGTAAAAAGCGATAATAAACTTAATTAAGGATCCGAATTC-
GAGCTCCGTCGACAAGCTTGCGGCCGCACTCGAGCACCTGCAGGCATGCAAGCTCTAGAGGCAAG-
GCTAGGTGGAGGCTCAGTG
Forward Primer: ATAAGGCTCGTATGATATATTCAGG (same as pVY-10 forward primer)
Reverse Primer: TGCCTCTAGAGCTTGCAT (same as pVY-10 reverse primer)
Vector template: pET24a-mScarletI-v2 (plasmid map can be provided upon request)
Restriction Enzymes: NdeI and XhoI

pVY-12: CAT-tag3
CAT-tag3 TWIST template:
GAAGTGCCATTCCGCCTGACCTCTTTAAGAAGGAGATATACCATGGGTCATCACCACCACCATCAC-
GGTGGCGCTGAGAAAAAAATCACTGGATATACCACCGTTGATATATCCCAATGGCATCGTAAAGAA-
CATTTTGAGGCATTTCAGTCAGTTGCTCAATGTACCTATAACCAGACCGTTCAGCTGGATATTACGG-
CCTTTTTAAAGACCGTAAAGAAAAATAAGCACAAGTTTTATCCGGCCTTTATTCACATTCTTGCCCG-
CCTGATGAATGCTCATCCGGAATTCCGTATGGCAATGAAAGACGGTGAGCTGGTGATATGGGATAGT-
GTTCACCCTTGTTACACCGTTTTCCATGAGCAAACTGAAACGTTTTCATCGCTCTGGAGTGAATAC-
CACGACGATTTCCGGCAGTTTCTACACATATATTCGCAAGATGTGGCGTGTTACGGTGAAAACCTG-
GCCTATTTCCCTAAAGGGTTTATTGAGAATATGTTTTTCGTCTCAGCCAATCCCTGGGTGAGTTTCAC-
CAGTTTTGATTTAAACGTGGCCAATATGGACAACTTCTTCGCCCCCGTTTTCACCATGGGCAAATAT-
TATACGCAAGGCGACAAGGTGCTGATGCCGCTGGCGATTCAGGTTCATCATGCCGTCTGTGATG-
GCTTCCATGTCGGCAGAATGCTTAATGAATTACAACAGTACTGCGATGAGTGGCAGGGCGGGG-
CGGGAGGAAGCAAGAAACGCAAGAAGCGTGGCGGTAGCAAAAAGCGTAAGAAGCGTGGAG-
GTTCAAAGAAACGTAAAAAGCGATAATAACTCGAGTCTGGTAAAGAAACCGCTGCAGGCTAGGTG-
GAGGCTCAGTG
Forward Primer: CTTTAAGAAGGAGATATACCATGGG
Reverse Primer: GCAGCGGTTTCTTTACCAGA
Vector Template: sfGFP plasmid
Restriction Enzymes: NcoI and XhoI
Note: for the cloning of CAT-tag3, only the vector template was digested with restriction enzymes. The digested vector was then mixed with purified CAT-tag3 insert obtained by PCR amplification of the TWIST template using the forward and reverse primers listed. The insert and backbone were annealed using NEBuilder HiFi DNA Assembly (described below).

The following plasmids were constructed using HiFi Assembly. Briefly, PCR reactions were performed and PCR products were purified as described above. Purified fragments were assembled using NEBuilder HiFi DNA As-

sembly Master Mix and were annealed at 50 °C for 15 min. The assembled plasmid was transformed into NEB5α and following sequence verification into NiCo21(DE3) cells. The sequences of plasmids were confirmed by Sanger sequencing (Genewiz).

pVY-13: mScarletI
mScarletI Template: pEB2-mScarletI from Addgene (#104007)
Forward Primer: TTTAAGAAGGAGGAGTACATATGGGTCATCACCACCACCATCACGGTGGCGCTAG-TAAAGGAGAAGCTGTGATTAAAG
Reverse Primer: CTTTGTTAGCAGCCGGATCTTTATTATTTGTATAGTTCATCCATGC
pET24a vector Template: pET24a-ELP[V-30] from Addgene (#67014)
Forward Primer: AGCGCCACCGTGATGGTGGTGGTGATGACCCATATGTACTCCTCCTTCTTAAAGTTA-AACAAAAT
Reverse Primer: TAAAGATCCGGCTGCTAACAAAGC

pVY-14: TC-CAT
CAT Template: CAT plasmid[1]
Forward Primer: ACTTTAAGAAGGAGATATACATGGGTTGTTGCCCTGGGTGTTGTGGTGGCGCT-GAGAAAAAAATCACTGGATATAC
Reverse Primer: ATGAGTATTCAACATTTCCG
pET24a vector template: CAT plasmid
Vector Forward Primer: CGGAAATGTTGAATACTCATACTCTTCCTTTTTCAATCATG
Vector Reverse Primer: GTATATCTCCTTCTTAAAGTTAAAC

pVY-15: TC-CAT-tag3
CAT Template: pVY-12
Forward Primer: ACTTTAAGAAGGAGATATACATGGGTTGTTGCCCTGGGTGTTGTGGTGGCGCT-GAGAAAAAAATCACTGGATATAC
Reverse Primer: ATGAGTATTCAACATTTCCG
pET24a vector template: CAT plasmid
Vector Forward Primer: CGGAAATGTTGAATACTCATACTCTTCCTTTTTCAATCATG (same as pVY-14 vector forward primer)
Vector Reverse Primer: GTATATCTCCTTCTTAAAGTTAAAC (same as pVY-14 vector reverse primer)

pVY-16: TC-TEM1
Template: sfGFP plasmid
Forward Primer: TTTAAGAAGGAGATATACATATGGGTTGTTGCCCTGGGTGTTGTGGTGGCGCTAG-TATTCAACATTTCCGTG
Reverse Primer: GCCGTTAATTAAGTTTATTACCAATGCTTAATCAGTGAG
pBAD vector template: p2-39 mScarletI-tag2 (plasmid map can be provided upon request)
Vector Forward Primer: TAATAAACTTAATTAACGGCAC
Vector Reverse Primer: ATGTATATCTCCTTCTTAAAGTTAAAC

pVY-17: TC-TEM1-tag3
Template: sfGFP plasmid
Forward Primer: TTTAAGAAGGAGATATACATATGGGTTGTTGCCCTGGGTGTTGTGGTGGCGCTAG-TATTCAACATTTCCGTG
Reverse Primer: TTGCGTTTCTTGCTTCCTCCCCAATGCTTAATCAGTGAG
pBAD vector template: p2-39 mScarletI-tag2 (plasmid map can be provided upon request)
Vector Forward Primer: GGAGGAAGCAAGAAACGC
Vector Reverse Primer: ATGTATATCTCCTTCTTAAAGTTAAACAAAATTATTTC

pVY-18: TC-sfGFP

TC-sfGFP Template: sfGFP plasmid
Forward Primer: ACTTTAAGAAGGAGATATACCATGGGTTGTTGCCCTGGGTGTTGTGGTGGCGCTAG-CAAAGGTGAAGAG
Reverse Primer: ATGAGTATTCAACATTTCCG
pET vector template: sfGFP plasmid
Vector Forward Primer: CGGAAATGTTGAATACTCATACTCTTCCTTTTTCAATCATG
Vector Reverse Primer: GTATATCTCCTTCTTAAAGTTAAAC

pVY-19: TC-sfGFP-tag3

TC-sfGFP-tag3 Template: pVY-3
Forward Primer: ACTTTAAGAAGGAGATATACCATGGGTTGTTGCCCTGGGTGTTGTGGTGGCGCTAG-CAAAGGTGAAGAG (same as pVY-18 forward primer)
Reverse Primer: ATGAGTATTCAACATTTCCG (same as pVY-18 reverse primer)
pET vector template: pVY-3
Vector Forward Primer: CGGAAATGTTGAATACTCATACTCTTCCTTTTTCAATCATG (same as pVY-18 vector forward primer)
Vector Reverse Primer: GTATATCTCCTTCTTAAAGTTAAAC (same as pVY-18 vector reverse primer)

Amino Acid Sequence of GFP, mScarletI, TEM1, and CAT variants:
His6x and TC tags with linkers (GGA) are underlined. Cationic tags are **bolded and underlined.** Tagged sfGFP variants contain an additional lysine residue (**bolded** but not underlined) between the C-terminus of sfGFP and N-terminus of the peptide tag because sfGFP has a net charge of -7 without this additional lysine. This lysine residue was included to ensure charge-equivalence between respective tagged variants and was not added to variants containing GFP(0) or GFP(+6) globular domains.

His6x-sfGFP-tag1

MGHHHHHHGGASKGEELFTGVVPILVELDGDVNGHKFSVRGEGEGDATNGKLTLKFICTTGKLPVP-WPTLVTTLTYGVQCFSRYPDHMKQHDFFKSAMPEGYVQERTISFKDDGTYKTRAEVKFEGDTLVNRI-ELKGIDFKEDGNILGHKLEYNFNSHNVYITADKQKNGIKANFKIRHNVEDGSVQLADHYQQNTPIGDG-PVLLPDNHYLSTQSALSKDPNEKRDHMVLLEFVTAAGITHGMDELYK**KGGSKKRKKR**

His6x-sfGFP-tag2

MGHHHHHHGGASKGEELFTGVVPILVELDGDVNGHKFSVRGEGEGDATNGKLTLKFICTTGKLPVP-WPTLVTTLTYGVQCFSRYPDHMKQHDFFKSAMPEGYVQERTISFKDDGTYKTRAEVKFEGDTLVNRI-ELKGIDFKEDGNILGHKLEYNFNSHNVYITADKQKNGIKANFKIRHNVEDGSVQLADHYQQNTPIGDG-PVLLPDNHYLSTQSALSKDPNEKRDHMVLLEFVTAAGITHGMDELYK**KGGSKKRKKRGGSKKRK-KR**

His6x-sfGFP-tag3

MGHHHHHHGGASKGEELFTGVVPILVELDGDVNGHKFSVRGEGEGDATNGKLTLKFICTTGKLPVP-WPTLVTTLTYGVQCFSRYPDHMKQHDFFKSAMPEGYVQERTISFKDDGTYKTRAEVKFEGDTLVNRI-ELKGIDFKEDGNILGHKLEYNFNSHNVYITADKQKNGIKANFKIRHNVEDGSVQLADHYQQNTPIGDG-PVLLPDNHYLSTQSALSKDPNEKRDHMVLLEFVTAAGITHGMDELYK**KGGSKKRKKRGGSKKRK-KRGGSKKRKKR**

His6x-GFP(0)-tag1

MGHHHHHHGGASKGERLFTGVVPILVELDGDVNGHKFSVRGEGEGDATNGKLTLKFICTTGKLPVP-WPTLVTTLTYGVQCFSRYPDHMKQHDFFKSAMPEGYVQERTISFKDDGTYKTRAEVKFEGDTLVNRI-ELKGRDFKEDGNILGHKLEYNFNSHNVYITADKQKNGIKANFKIRHNVEDGSVQLADHYQQNTPIG-

DGPVLLPRNHYLSTQSALSKDPKEKRDHMVLLEFVTAAGITHGMDERYK**GGSKKRKKR**

His6x-GFP(0)-tag2
MGHHHHHHGGASKGERLFTGVVPILVELDGDVNGHKFSVRGEGEGDATNGKLTLKFICTTGKLPVP-WPTLVTTLTYGVQCFSRYPDHMKQHDFFKSAMPEGYVQERTISFKDDGTYKTRAEVKFEGDTLVNRI-ELKGRDFKEDGNILGHKLEYNFNSHNVYITADKQKNGIKANFKIRHNVEDGSVQLADHYQQNTPIG-DGPVLLPRNHYLSTQSALSKDPKEKRDHMVLLEFVTAAGITHGMDERYK**GGSKKRKKRGGSKKRK-KR**

His6x-GFP(0)-tag3
MGHHHHHHGGASKGERLFTGVVPILVELDGDVNGHKFSVRGEGEGDATNGKLTLKFICTTGKLPVP-WPTLVTTLTYGVQCFSRYPDHMKQHDFFKSAMPEGYVQERTISFKDDGTYKTRAEVKFEGDTLVNRI-ELKGRDFKEDGNILGHKLEYNFNSHNVYITADKQKNGIKANFKIRHNVEDGSVQLADHYQQNTPIG-DGPVLLPRNHYLSTQSALSKDPKEKRDHMVLLEFVTAAGITHGMDERYK**GGSKKRKKRGGSKKRK-KRGGSKKRKKR**

His6x-GFP(+6)-tag1
MGHHHHHHGGASKGERLFTGVVPILVELDGDVNGHKFSVRGEGEGDATNGKLTLKFICTTGKLPVP-WPTLVTTLTYGVQCFSRYPKHMKQHDFFKSAMPEGYVQERTISFKKDGTYKTRAEVKFEGRTLVNRI-ELKGRDFKEDGNILGHKLEYNFNSHNVYITADKQKNGIKANFKIRHNVEDGSVQLADHYQQNTPIG-DGPVLLPRNHYLSTQSALSKDPKEKRDHMVLLEFVTAAGITHGMDERYK**GGSKKRKKR**

His6x-GFP(+6)-tag2
MGHHHHHHGGASKGERLFTGVVPILVELDGDVNGHKFSVRGEGEGDATNGKLTLKFICTTGKLPVP-WPTLVTTLTYGVQCFSRYPKHMKQHDFFKSAMPEGYVQERTISFKKDGTYKTRAEVKFEGRTLVNRI-ELKGRDFKEDGNILGHKLEYNFNSHNVYITADKQKNGIKANFKIRHNVEDGSVQLADHYQQNTPIG-DGPVLLPRNHYLSTQSALSKDPKEKRDHMVLLEFVTAAGITHGMDERYK**GGSKKRKKRGGSKKRK-KR**

His6x-GFP(+6)-tag3
MGHHHHHHGGASKGERLFTGVVPILVELDGDVNGHKFSVRGEGEGDATNGKLTLKFICTTGKLPVP-WPTLVTTLTYGVQCFSRYPKHMKQHDFFKSAMPEGYVQERTISFKKDGTYKTRAEVKFEGRTLVNRI-ELKGRDFKEDGNILGHKLEYNFNSHNVYITADKQKNGIKANFKIRHNVEDGSVQLADHYQQNTPIG-DGPVLLPRNHYLSTQSALSKDPKEKRDHMVLLEFVTAAGITHGMDERYK**GGSKKRKKRGGSKKRK-KRGGSKKRKKR**

His6x-mScarletI
MGHHHHHHGGASKGEAVIKEFMRFKVHMEGSMNGHEFEIEGEGEGRPYEGTQTAKLKVTKGG-PLPFSWDILSPQFMYGSRAFIKHPADIPDYYKQSFPEGFKWERVMNFEDGGAVTVTQDTSLEDGTLI-YKVKLRGTNFPPDGPVMQKKTMGWEASTERLYPEDGVLKGDIKMALRLKDGGRYLADFKTTYKAK-KPVQMPGAYNVDRKLDITSHNEDYTVVEQYERSEGRHSTGGMDELYK

His6x-mScarletI-tag2
MGHHHHHHGGASKGEAVIKEFMRFKVHMEGSMNGHEFEIEGEGEGRPYEGTQTAKLKVTKGG-PLPFSWDILSPQFMYGSRAFIKHPADIPDYYKQSFPEGFKWERVMNFEDGGAVTVTQDTSLEDGTLI-YKVKLRGTNFPPDGPVMQKKTMGWEASTERLYPEDGVLKGDIKMALRLKDGGRYLADFKTTYKAK-KPVQMPGAYNVDRKLDITSHNEDYTVVEQYERSEGRHSTGGMDELYK**GGSKKRKKRGGSKKRKKR**

His6x-mScarletI-tag3
MGHHHHHHGGASKGEAVIKEFMRFKVHMEGSMNGHEFEIEGEGEGRPYEGTQTAKLKVTKGG-

PLPFSWDILSPQFMYGSRAFIKHPADIPDYYKQSFPEGFKWERVMNFEDGGAVTVTQDTSLEDGTLI-YKVKLRGTNFPPDGPVMQKKTMGWEASTERLYPEDGVLKGDIKMALRLKDGGRYLADFKTTYKA-KKPVQMPGAYNVDRKLDITSHNEDYTVVEQYERSEGRHSTGGMDELYK**GGSKKRKKRGGSKKRK-KRGGSKKRKKR**

TC-CAT
MGCCPGCCGGAEKKITGYTTVDISQWHRKEHFEAFQSVAQCTYNQTVQLDITAFLKTVKKNKHK-FYPAFIHILARLMNAHPEFRMAMKDGELVIWDSVHPCYTVFHEQTETFSSLWSEYHDDFRQFLHI-YSQDVACYGENLAYFPKGFIENMFFVSANPWVSFTSFDLNVANMDNFFAPVFTMGKYYTQGDKVLM-PLAIQVHHAVCDGFHVGRMLNELQQYCDEWQGGA

TC-CAT-tag3
MGCCPGCCGGAEKKITGYTTVDISQWHRKEHFEAFQSVAQCTYNQTVQLDITAFLKTVKKNKHK-FYPAFIHILARLMNAHPEFRMAMKDGELVIWDSVHPCYTVFHEQTETFSSLWSEYHDDFRQFLHI-YSQDVACYGENLAYFPKGFIENMFFVSANPWVSFTSFDLNVANMDNFFAPVFTMGKYYTQGDKVLM-PLAIQVHHAVCDGFHVGRMLNELQQYCDEWQGGA**GGSKKRKKRGGSKKRKKRGGSKKRKKR**

TC-TEM1
MGCCPGCCGGASIQHFRVALIPFFAAFCLPVFAHPETLVKVKDAEDQLGARVGYIELDLNSGKILES-FRPEERFPMMSTFKVLLCGAVLSRIDAGQEQLGRRIHYSQNDLVEYSPVTEKHLTDGMTVRELCSAA-ITMSDNTAANLLLTTIGGPKELTAFLHNMGDHVTRLDRWEPELNEAIPNDERDTTMPVAMATTL-RKLLTGELLTLASRQQLIDWMEADKVAGPLLRSALPAGWFIADKSGAGERGSRGIIAALGPDGKPSRIV-VIYTTGSQATMDERNRQIAEIGASLIKHW

TC-TEM1-tag3
MGCCPGCCGGASIQHFRVALIPFFAAFCLPVFAHPETLVKVKDAEDQLGARVGYIELDLNSGKILES-FRPEERFPMMSTFKVLLCGAVLSRIDAGQEQLGRRIHYSQNDLVEYSPVTEKHLTDGMTVRELCSAA-ITMSDNTAANLLLTTIGGPKELTAFLHNMGDHVTRLDRWEPELNEAIPNDERDTTMPVAMATTL-RKLLTGELLTLASRQQLIDWMEADKVAGPLLRSALPAGWFIADKSGAGERGSRGIIAALGPDGKPSRIV-VIYTTGSQATMDERNRQIAEIGASLIKHW**GGSKKRKKRGGSKKRKKRGGSKKRKKR**

TC-sfGFP
MGCCPGCCGGASKGEELFTGVVPILVELDGDVNGHKFSVRGEGEGDATNGKLTLKFICTTGKLPVP-WPTLVTTLTYGVQCFSRYPDHMKQHDFFKSAMPEGYVQERTISFKDDGTYKTRAEVKFEGDTLVNRI-ELKGIDFKEDGNILGHKLEYNFNSHNVYITADKQKNGIKANFKIRHNVEDGSVQLADHYQQNTPIGDG-PVLLPDNHYLSTQSALSKDPNEKRDHMVLLEFVTAAGITHGMDELYK

TC-sfGFP-tag3
MGCCPGCCGGASKGEELFTGVVPILVELDGDVNGHKFSVRGEGEGDATNGKLTLKFICTTGKLPVP-WPTLVTTLTYGVQCFSRYPDHMKQHDFFKSAMPEGYVQERTISFKDDGTYKTRAEVKFEGDTLVNRI-ELKGIDFKEDGNILGHKLEYNFNSHNVYITADKQKNGIKANFKIRHNVEDGSVQLADHYQQNTPIGDG-PVLLPDNHYLSTQSALSKDPNEKRDHMVLLEFVTAAGITHGMDELYK**KGGSKKRKKRGGSKKRK-KRGGSKKRKKR**

Supplementary Table 1. Cationic peptide tags reported herein

Tags	Tag Sequence	Net Charge
tag1	GGSKKRKKR	+6
tag2	GGSKKRKKR GGSKKRKKR	+12
tag3	GGSKKRKKR GGSKKRKKR GGSKKRKKR	+18

Electrostatic maps of GFP variants

The PDB file for sfGFP (PDB ID: 2B3P) was obtained from the RCSB protein data bank and loaded in PyMOL v2.4.1 (Schrödinger, LLC). Using the PDB file for sfGFP as a template, GFP(0) and GFP(+6) were made by introducing residue substitutions in PyMOL. PDBs of the minimized peptide tag structures were obtained using PEP-FOLD3.[2–4] The C-terminus of the globular domain was appended to the N-terminus of the tags in PyMOL and electrostatic surface maps were generated using the ABPS Electrostatics plugin.

Constraints for image analysis using MicrobeJ

Briefly, images stacks were subjected to a rolling ball background subtraction with a 100 pixel radius. Stacks of images were individually thresholded using the Li method in MicrobeJ (version 15.3l (14)) and cells that met the area, length, width, and angularity constraints listed in the table below were identified and considered for analysis. The following constraints were used for all image stacks: circularity = 0.25 – max; curvature = 0 – max; sinuosity = 0 – max; solidity = 0 – max; intensity = 0 – max. Bacteria were detected using the fit shape, rod-shaped mode.

Supplementary Table 2. MicrobeJ image analysis constraints

Images	Area (pixels^2)	Length (pixels)	Width (pixels)	Angularity
Isotropic GFP variants at 2 and 8 h (unless specified other-wise)	100-800	20 - max	0-20	0 – 0.4
Isotropic GFP variants at 24 h (unless specified otherwise) Also used for Tagged GFP variants with cell clumps at 2, 4, 6, and 24 h	100-800	20 - max	0-15	0 – 0.4
Isotropic GFP(+24) and (+36) at 8 and 24 h	100-800	25 - max	0-20	0 – 0.4
Tagged GFP variants at 2, 4, 6, 24 h (unless otherwise speci-fied)	100-800	10 - max	0-25	0 – 0.45
mScarletI variants at 2, 4, 24 h	100-2000	25 - max	0-25	0 – 0.45

Image analysis using a custom MATLAB script

Briefly, the nine central pixels of each cell image were averaged to determine the approximate average pixel intensity for non-condensate areas for each cell. All pixels in a cell with 20% higher intensity than this approximated average intensity were classified as condensates. This intensity threshold was selected after testing intensity thresholds from 0 to 30% higher than the approximated average intensity on isotropic GFP variants that had been separately categorized as condensate-containing or non-condensate containing[5]. The 20% threshold most accurately and clearly distinguished condensate-containing variants from non-condensate-containing variants. A duplicate image with all non-condensate pixels set to zero was then created for each cell. The number of non-zero pixels in the duplicate image was divided by the number of pixels in the original image to determine the percentage of each cell that contained condensates. This percentage was then used to determine the number of condensate-containing cells by counting cells with a non-zero percentage as condensate-containing. The number of condensate-containing cells was thus counted for each experiment and divided by the total number of cells identified by MicrobeJ to determine the fraction of condensate-containing cells.

Cell growth assay

Cells were grown as described in the "Protein expression" section (in the Methods section in the main text) and aliquots were collected at 0, 2, 4, 6, 8, 10, 12, 20, 22, and 24 h after induction. At each time point, aliquots of each sample were diluted 5- or 10-fold with LB media to a total volume of 1 mL. 150 µL of each diluted sample was then transferred to a 96-well black flat bottom tissue culture-treated plate (Corning REF#3904) in triplicate. GFP fluorescence intensity and Tecan OD_{600} were measured for all samples in a plate reader (Tecan Infinite M200 Pro; excitation at 488 nm, emission at 530 nm, gain: 75). For mScarletI variants, Tecan OD_{750} and RFP fluorescence intensity were measured (excitation at 569 nm, emission at 593 nm, gain: 75). The fluorescence intensity and

Tecan OD of wells containing LB media were subtracted from raw fluorescence intensity values and raw Tecan OD values, respectively. Background subtracted fluorescence intensity values were then divided by the adjusted Tecan OD. This ratio of fluorescence intensity to Tecan OD was averaged across three biological replicates for each protein variant and then normalized to the highest average fluorescence intensity/Tecan OD ratio amongst all GFP variants (GFP(0), 22 h).

Solubility assay

Cell cultures (1 L) were grown and expressed as described in the "Protein Expression" section in the Main Methods. Cultures were incubated at 25 ºC for 24 h after induction with IPTG. 900 mL of each culture was then harvested by centrifugation (Thermo Scientific Sorvall Legend XTR) at 4000 rpm for 10 min in a swinging bucket rotor (Thermo Scientific TX-750). The cell pellet was resuspended in 13.5 mL of cell lysis buffer without NaCl (50 mM NaH_2PO_4, pH 8.0) and divided into 4.5 mL aliquots. All samples were subjected to one freeze-thaw cycle before being lysed by sonication on ice (2 s on, 4 s off at 40% amplitude for 5 min of sonics) with a 1/8 in. (diameter) microtip probe. The lysate was then centrifuged at 10,000 rpm for 30 minutes at 4 °C in a fixed angle rotor (Thermo Scientific, Fiberlite F15-8x50cy). The pellets were washed by resuspending in 4.5 mL of lysis buffer without NaCl, divided into three 1.5 mL aliquots in 2 mL microcentrifuge tubes. Samples were then pelleted at 16,000 x g in a microcentrifuge (Thermo Scientific Sorvall Legend Micro 21R) for 30 minutes at 4 °C. The supernatant was removed, and 1.4 mL of cell lysis buffer containing different concentrations of NaCl (0 mM, 100 mM or 1 M) was then added to each of the pellets to solubilize the protein condensates. Finally, samples were centrifuged again at 16,000 x g for 30 minutes at 4 °C. All supernatants (soluble fractions) and pellets (insoluble fractions) were run on Bolt™ 4-12% Bis-Tris Plus gels (Invitrogen) and imaged on a Gel Doc XR+ System (Bio-Rad) to determine condensate solubility. Analysis of gel images was done as described previously[5]. Briefly, a 100-pixel radius rolling ball background subtraction was performed in Fiji for each gel image. Rectangular ROIs were drawn around the band corresponding to GFP or spanning the height of the entire lane to capture all proteins. Histograms were generated from these ROIs, and line segments were drawn at the base of the peaks. The area under the curve was measured in order to measure the band intensities of GFP and total protein for each lane. The fraction of GFP in the soluble fraction was determined by dividing the band intensity of GFP in the soluble fraction by the band intensity of total protein in the soluble fraction. For each variant, the fraction of GFP in the soluble fraction was then normalized in GraphPad Prism (version 9.1.0) to the fraction extracted in 1 M NaCl and plotted as the normalized ratio of GFP to total protein in the supernatant.

Protein purification for *in vitro* tests

Cell cultures (1 L) were grown and expressed as described in the "Protein Expression" section in the Main Methods. Cultures were incubated at 25 ºC for 16-18 h after induction with IPTG. Cultures were then harvested by centrifugation (Thermo Scientific Sorvall Legend XTR) at 4000 rpm for 10 min in a swinging bucket rotor (Thermo Scientific TX-750). The cell pellet was resuspended in lysis buffer (50 mM NaH_2PO_4, 300 mM NaCl, pH 8.0) and lysed by sonication on ice (2 s on, 4 s off at 60% amplitude for 8 min of sonics) using a 0.5 inch probe. The lysate was then centrifuged at 10,000 rpm for 30 min and the supernatant (clarified lysate) was collected for purification using immobilized metal affinity chromatography. 8 mL of His-Pur Ni-NTA slurry (Thermo Scientific) was used to purify protein harvested from 1 L cell culture. Briefly, the resin was equilibrated with lysis buffer and the clarified lysate was incubated with the resin for 5-10 min at room temperature. The resin was then washed in lysis buffer containing 50 mM imidazole and His-tagged proteins were eluted with lysis buffer containing 250 mM imidazole. All flow through, wash, and elution fractions were collected for analysis on a Bolt™ 4-12% Bis-Tris Plus gel (Invitrogen). Pure fractions were concentrated using Amicon Ultra centrifugal filter units with a 10 kDa molecular weight cutoff (Millipore Sigma) and then dialyzed against a physiological buffer (70 mM K_2HPO_4, 60 mM KCl, 40 mM NaCl, pH 7.4), which mimics the cytoplasmic ion concentrations in *E. coli.* The buffer was changed 7 times with at least 3 h equilibration between each buffer exchange.

MALDI-TOF mass spectrometry

The mass of purified GFP(+6)-tag3 used for *in vitro* assays was verified by MALDI-TOF mass spectrometry prior

to use in experiments. As reported previously, salts were removed from the protein stock solution by performing a buffer exchange into Milli-Q water using 10 kDa MWCO Amicon Ultra centrifugal filter (Millipore Sigma)[5]. A 10 mg/mL sinapinic acid matrix (in 7:3 water:acetonitrile with 0.1% trifluoroacetic acid) was prepared and the GF-P(+6)-tag3 sample was premixed at a 4:6 sample:matrix ratio before being spotted on a grounded stainless steel target. A calibration was performed with Protein Standard II (Bruker) before collecting MALDI-TOF spectra on a Bruker ultrafleXtreme MALDI TOF/TOF. All spectra were obtained from the Columbia University Chemistry Department Mass Spectrometry Facility.

In vitro turbidity assays and microscopy of coacervates

Protein stock solutions were prepared at 1 mg/mL in physiological buffer (70 mM K_2HPO_4, 60 mM KCl, 40 mM NaCl, pH 7.4). 1 mg/mL RNA stock solutions were prepared fresh by dissolving total RNA from torula yeast type VI (Sigma-Aldrich) in physiological buffer and adjusting to pH 7.4. Both stock solutions were filtered using a 0.22 µm SFCA Thermo Scientific Nalgene 25 mm Syringe Filter. Protein and RNA stock solutions were mixed at varying mass ratios in a tissue culture-treated polystyrene 96-well half-area plate (Corning, REF#3697). Mixing ratios were prepared in triplicate, and absorbance at 600 nm was measured for each well on a plate reader (Tecan Infinite M200 Pro). Turbidity was then calculated using the equation, Turbidity = $100 - 10^{2-A}$. Following these measurements, GFP/RNA mixtures with high turbidity were prepared in a 384-well glass-bottom plate (Cellvis) and imaged on an Evos FL Auto 2 inverted fluorescence microscope (Invitrogen). Images were acquired using a 20X 0.40 NA Plan Fluor objective on GFP (λ_{ex} = 479-522 nm; λ_{em} = 525-550 nm; EVOS GFP light cube) and brightfield channels.

NaCl titration to determine critical salt concentration

Protein and RNA stock solutions were prepared in physiological buffer (70 mM K_2HPO_4, 60 mM KCl, 40 mM NaCl, pH 7.4) as described above. GFP(+6)-tag3 and RNA were mixed at the mass fraction that provided the highest turbidity, which was determined empirically (0.6 GFP mass fraction). The sample was prepared to a final volume of 1 mL in a 4 mL PMMA cuvette and stirred continuously at 22 °C, 500 rpm. A solution of 5 M NaCl dissolved in physiological buffer was titrated into the sample via 1 µL additions and the absorbance at 600 nm was collected following each addition using a Cary Ultraviolet/visible spectrophotometer. Measurements were collected until the absorbance stabilized. Absorbance was then converted to turbidity as described above and then plotted as a function of ionic strength in Prism GraphPad (version 9.1.0). Ionic strength was calculated using the formula: $I = \frac{1}{2} \sum_i^n c_i z_i^2$, where n is the number of ions in solution, c_i is the concentration of ion species i, and z_i is the valence of ion species i. For ionic strength calculations, we assumed the following dissociation reactions at pH 7.4: $K_2HPO_{4(s)} \rightarrow 2K^+_{(aq)} + HPO_4^{2-}{}_{(aq)}$; $KCl_{(s)} \rightarrow K^+_{(aq)} + Cl^-_{(aq)}$; and $NaCl_{(s)} \rightarrow Na^+_{(aq)} + Cl^-_{(aq)}$.

FlAsH staining of TC-AmpR and -AmpR-tag3 cultures

Overnight cultures were back diluted to OD_{600} = 0.1 in 1 mL of LB media supplemented with 25 µg/mL chloramphenicol, 0.2% w/v arabinose, and 10 µM FlAsH-EDT_2 (Cayman Chemical) and incubated in 14 mL snap cap tubes in an incubator at 37 ºC with shaking at 225 rpm. After 2 h, the cultures were transferred to a 25 ºC incubator and allowed to shake at 225 rpm overnight. After 24 h, 100 µL of each culture was washed three times in an equal volume of BAL (British anti-Lewisite from Fisher Scientific) diluted to 1X in phosphate buffer saline, and adjusted to pH 7.4. Cells were washed in a microcentrifuge tube with centrifugation at 1,400 x g to collect the cell pellet between each washing step. Stained cells were then imaged on a 1% agarose pad as described in the "Optical Microscopy" section.

ReAsH-EDT staining of TC-CAT, TC-CAT-tag3, TC-sfGFP, and TC-sfGFP-tag3 cultures

Overnight cultures were back diluted to OD_{600} ~ 0.1 in 2 mL of LB media supplemented with 100 µg/ml ampicillin, and incubated in 14 mL snap cap tubes in an incubator at 37 ºC with shaking at 225 rpm. After 2 h, the cultures reached OD_{600} = 0.8 -1 and were induced with 1 mM IPTG. Cultures were subsequently stained with 1 µM ReAsH-EDT_2 (Invitrogen) and transferred to a 25 ºC incubator with shaking at 225 rpm overnight. 24 h after IPTG induction, 100 µL of each sample was collected in a microcentrifuge tube and washed three times in an

equal volume of 1X BAL in phosphate buffer saline, pH 7.4 before imaging on a 1.5% LB agarose pad using the protocol described in the "Optical microscopy" section.

Carbenicillin Sensitivity Spotting Assay

Overnight cultures of TC-TEM1 and TC-TEM1-tag3 were prepared as described above and then diluted to OD_{600} = 0.1 in LB media supplemented with 25 µg/ml chloramphenicol, 0.2 % (w/v) arabinose, and 10 µM FlAsH-EDT_2 (Fisher Scientific). Induced cultures were grown at 37 ºC, 225 rpm for 2 h. All cultures were normalized to the OD_{600} of the least dense sample (~0.7) in LB media. Normalized cultures were serially diluted 20-fold in LB media. 5 µL of all normalized cultures and their dilutions (20X and 400X) were then spotted onto LB agar plates supplemented with either 0.2 % (w/v) arabinose or 10 mM glucose, 25 µg/mL chloramphenicol, and varying concentrations of carbenicillin (0, 25, 50, 100, or 200 µg/mL). Samples on plates were allowed to dry fully before incubation in the dark at 25 ºC for ~30 hours and then imaged on a Gel Doc XR+ System (Bio-Rad). Samples from the plate were also picked into 20 µL LB media and imaged on a microscope slide to confirm the presence of condensates (as described in "Optical Microscopy").

Nucleic acid staining of cells with DAPI or SYTO17

GFP variants were expressed in *E. coli* as described in the "Protein Expression" section (in the Methods section of the main text). At 24 h post-induction, 250 µL of each cell culture were centrifuged at 1400 x g for 1 min and washed with 250 µL of 1X PBS. Cells were resuspended in an equal volume of 1X PBS and then 0.5 µL of a solution of stain was added. The DAPI (Invitrogen) solution was prepared at 5 mg/mL in DMF and the SYTO17 (Invitrogen) solution was prepared at 0.5 mM in DMSO. Stained cells were incubated in a dark cabinet at room temperature for 10 min before being spotted on a 1% agarose pad and imaged as described in the "Optical Microscopy" section. This protocol was adapted from a previously reported staining protocol.[5] Cells were imaged on the brightfield, GFP, DAPI (λ_{ex}= 357-444 nm; λ_{em}= 447-560 nm; EVOS DAPI light cube) or Texas Red channels (λ_{ex}= 585-629 nm; λ_{em}= 624-640 nm; Invitrogen Texas Red light cube) channels.

Spotting assay and analysis

Briefly, a saturated overnight culture was inoculated into LB supplemented with antibiotic and grown until the slowest growing culture reached OD_{600} ≥ 0.9 (~ 3 h). All samples were then normalized to OD_{600} = 0.9 in LB media supplemented with 100 µg/mL ampicillin and 200 µL were transferred to a 96-well flat clear bottom black microtiter plate (Corning #3904). Subsequent 5-fold serial dilutions were performed in LB media supplemented with 100 µg/mL ampicillin (5X, 125X, 625X, 3125X, 15625X, and 78125X). A 96-pin multi-blot replicator (VP Scientific, VP407) was then used to transfer ~1.5 µL of each sample to 100 µg/mL ampicillin LB agar plates with or without 1 mM IPTG. Transferred samples were then dried under the flame, incubated in the dark at 25 ºC for ~30 h, and then imaged on a scanner (Epson Perfection V850). Scanned images were analyzed using Fiji. Images were changed to 8-bit, inverted, and background subtracted (rolling ball with 100 pixel radius). The scale from all images was also removed. Circular ROIs of equal radii were drawn around all spots and the mean intensity of all ROIs was measured. The mean intensity of a blank spot on the plate with no cell growth was subtracted from the mean intensity of all samples. For each GFP variant, blank-subtracted mean intensities from all serial dilutions were normalized to those from the undiluted samples and then plotted in GraphPad Prism (version 9.1.0).

CAT and CAT-tag3 purification

CAT and CAT-tag3 were expressed as described in the "Protein Expression"in the Main Methods and 2 x 1 L cultures were harvested by centrifugation (Thermo Scientific Sorvall Legend XTR) at 4000 rpm for 10 min in a swinging bucket rotor (Thermo Scientific TX-750). The cell pellet was resuspended in 10 mM Tris HCl, pH 7.4 and lysed by two rounds of sonication on ice (2 s on, 4 s off at 60% amplitude for 4 min of sonics per round) using a 0.5" probe. The lysate was then centrifuged at 10,000 rpm for 45 min at 4 ºC and the supernatant (clarified lysate 1) was collected. The pellets were then resuspended in 15 mL of 1 M NaCl lysis buffer (50 mM NaH_2PO_4, 1 M NaCl, pH 8.0) and centrifuged again at 10,000 rpm for 45 min at 4 ºC. The supernatant (clarified lysate 2) was collected for purification using immobilized metal affinity chromatography. 16 mL of His-Pur Ni-NTA slurry

(Thermo Scientific) was used to purify the harvested protein (8 mL/1 L cell culture). For the purification of CAT, the resin was equilibrated with lysis buffer (50 mM NaH_2PO_4, 300 mM NaCl, pH 8.0) and clarified lysates 1 and 2 were incubated with the resin overnight in a 4 ºC deli fridge. The resin was then washed in lysis buffer containing 35 mM imidazole, followed by lysis buffer containing 120 mM imidazole before eluting His-tagged proteins in lysis buffer containing 250 mM imidazole. CAT-tag3 was purified in high salt buffer since this protein forms intracellular condensates. The procedures described for the purification of CAT were also used to purify CAT-tag3 except all buffers (lysis, equilibration, wash, and elution) contained 1 M NaCl instead of 300 mM NaCl. All flow through, wash, and elution fractions were collected for analysis on a Bolt™ 4-12% Bis-Tris Plus gel (Invitrogen). Pure fractions were concentrated using Amicon Ultra centrifugal filter units with a 10 kDa molecular weight cut-off (Millipore Sigma) and then dialyzed against 10 mM Tris HCl, 1 mM EDTA, 300 mM NaCl, pH 7.4. NaCl was added because both CAT variants formed precipitates in low salt buffer during dialysis. The buffer was changed 5 times with at least 2 h equilibration between each buffer exchange.

Enzyme activity assay

0.25 mg/mL stock solutions of purified proteins in 10 mM Tris HCl, 1 mM EDTA, 300 mM NaCl, pH 7.4 were prepared and concentrations were verified by BCA protein assay (Pierce). Chloramphenicol acetyltransferase activity assays were then conducted as instructed by Sigma Aldrich. Briefly, solutions of the following were prepared: (a) 100 mM Tris buffer, pH 7.8; (b) 2.5 mM 5,5'-Dithio-bis(2-Nitrobenzoic Acid) Solution, pH 7.8 (DTNB) in 100 mM Tris buffer (a); (c) 5.0 mM Acetyl Coenzyme A Solution in MilliQ water; (d) 0.3% (w/v) Chloramphenicol Solution. The chloramphenicol solution was prepared by first dissolving chloramphenicol in 0.1 mL of methanol and then adding MilliQ water. 10 mM Tris HCl, 1 mM EDTA, 300 mM NaCl, pH 7.4 was used as the enzyme diluent. 100 mM Tris buffer, DTNB, Acetyl CoA, and chloramphenicol were mixed in a 1.5 mL cuvette and allowed to equilibrate at room temperature for ~ 5 min. Once the A_{412} stabilized, either enzyme diluent (blank) or 0.25 mg/mL purified enzyme were added and mixed by pipetting. A_{412} was then monitored for 6 min.

Supplementary Figures

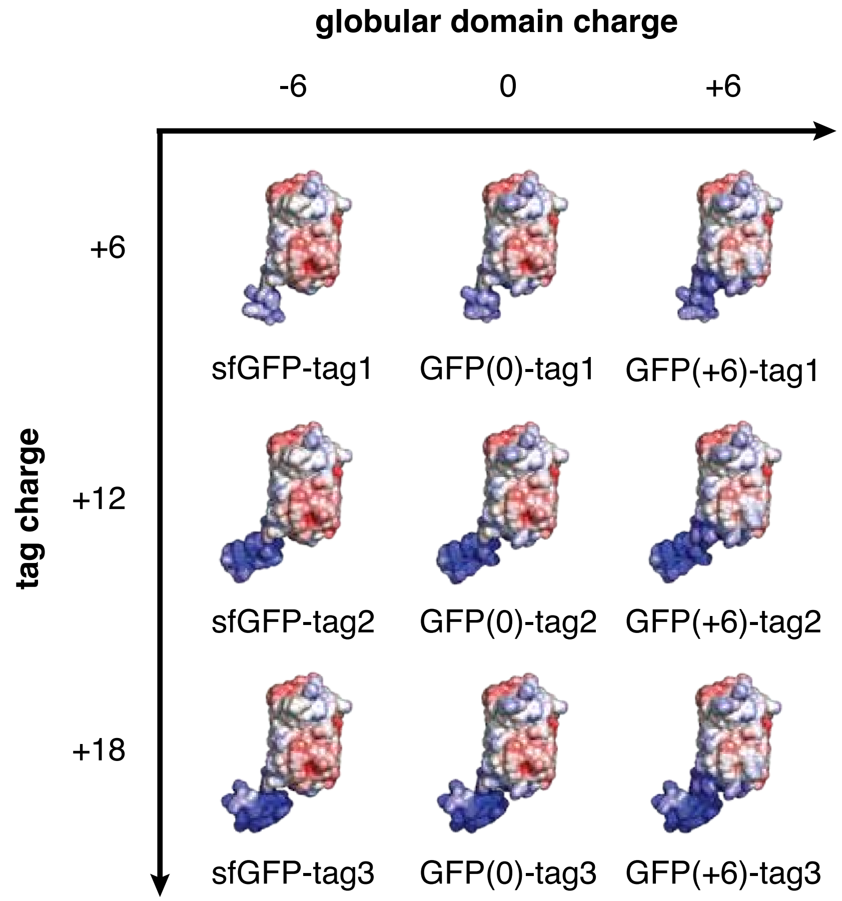

Supplementary Figure 1. Electrostatic surface representations of the cationic GFP variants investigated in this study. Each GFP variant is a combination of a globular domain (sfGFP, GFP(0), or GFP(+6)) and a cationic peptide tag, which consists of one or more repeats of the amino acid sequence, GGSKKRKKR. Additional sequence information can be found in the materials and methods. Electrostatic maps were generated using the ABPS Electrostatics plugin in PyMol. The point mutations on the globular domain were generated in PyMol using the mutagenesis feature. To create the GFP-peptide fusions, peptide structures were prepared using PEP-FOLD3 and were then appended to the C-terminus of the GFP globular domain in PyMol.

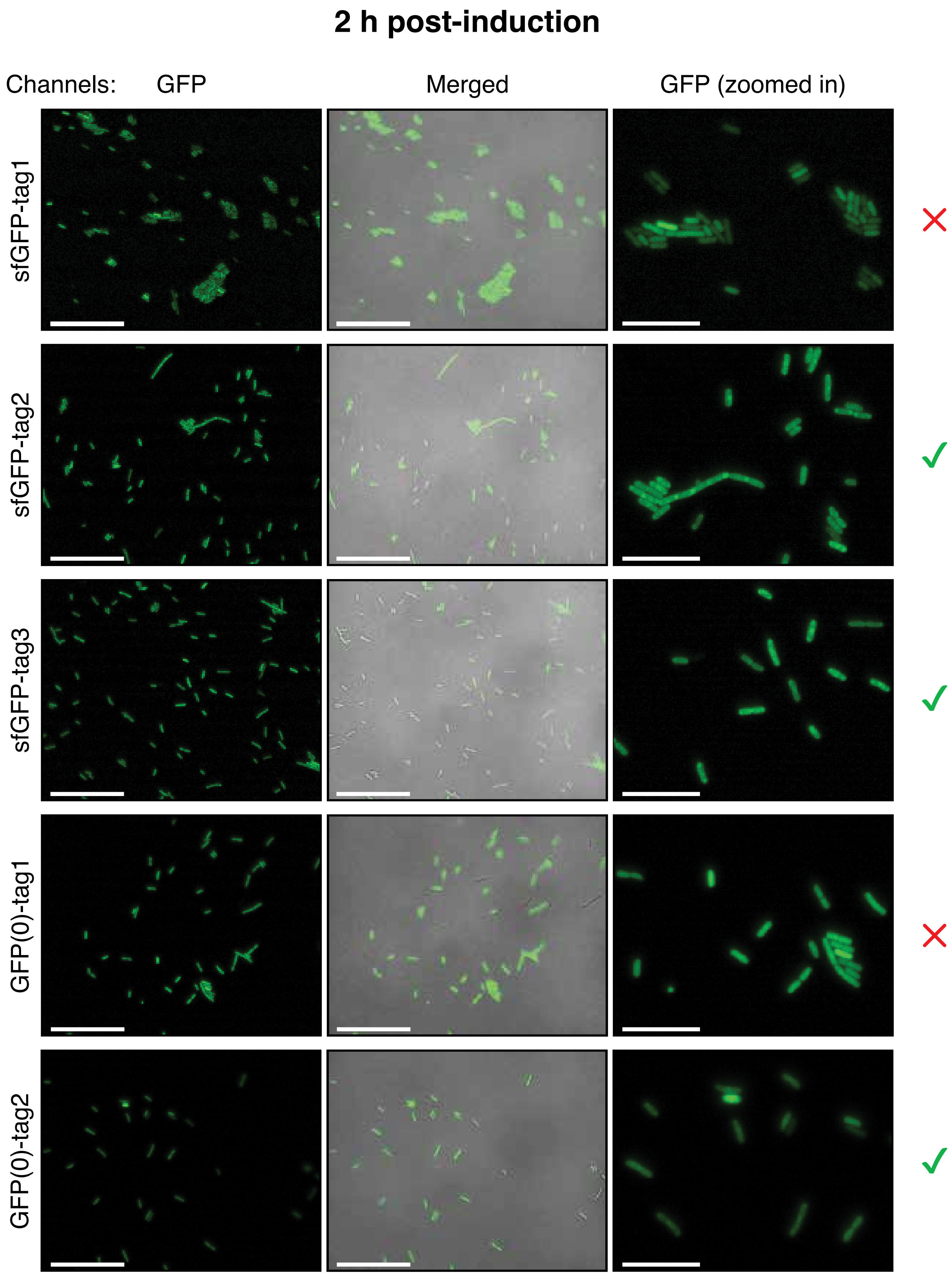
2 h post-induction
Channels:
GFP
Merged
GFP (zoomed in)
sfGFP-tag1
sfGFP-tag2
sfGFP-tag3
GFP(0)-tag1
GFP(0)-tag2
✗
✓
✓
✗
✓

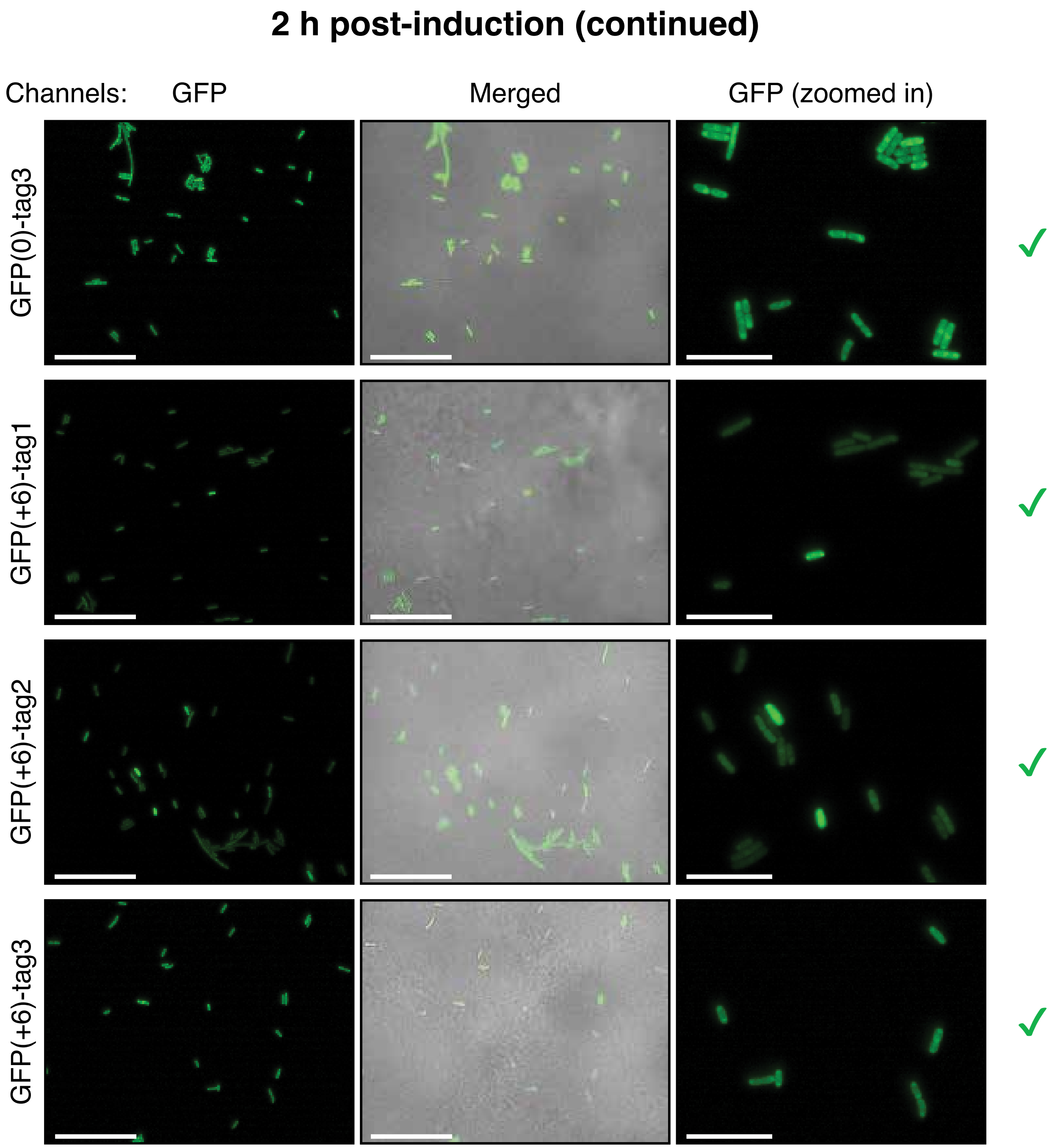

Supplementary Figure 2. Representative microscopy images of *E. coli* cells expressing each GFP variant at 2 h post-induction. GFP and merged (GFP and brightfield) images depict a population of cells for each variant. Scale bars are 25 μm for GFP (left) and merged (middle) images, and 10 μm for the zoomed-in (right) images. Green check marks indicate the presence of condensates and red “X”s indicate their absence. Similar results were observed in all the biological replicates (n=3) for each variant.

4 h post-induction

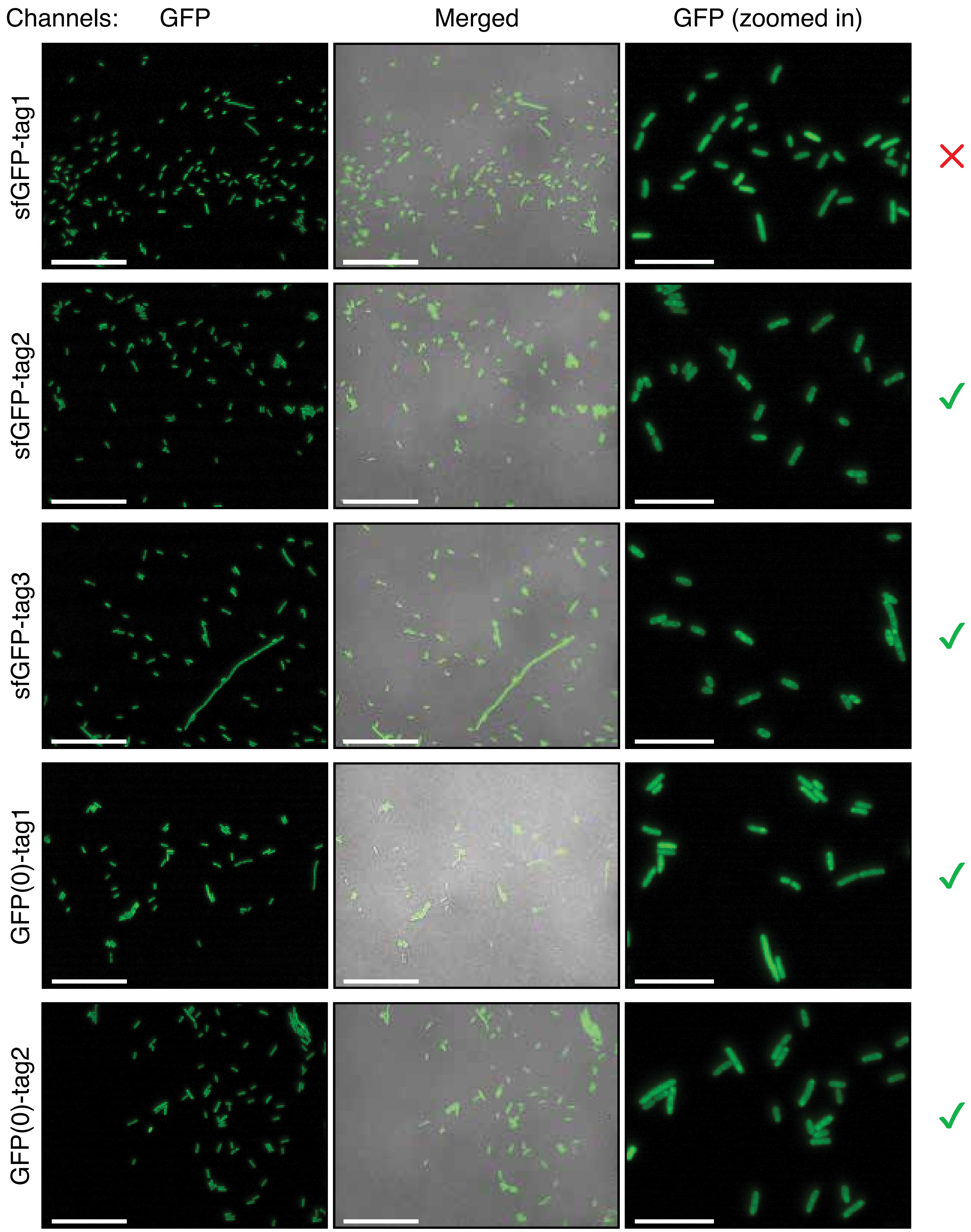

4 h post-induction (continued)

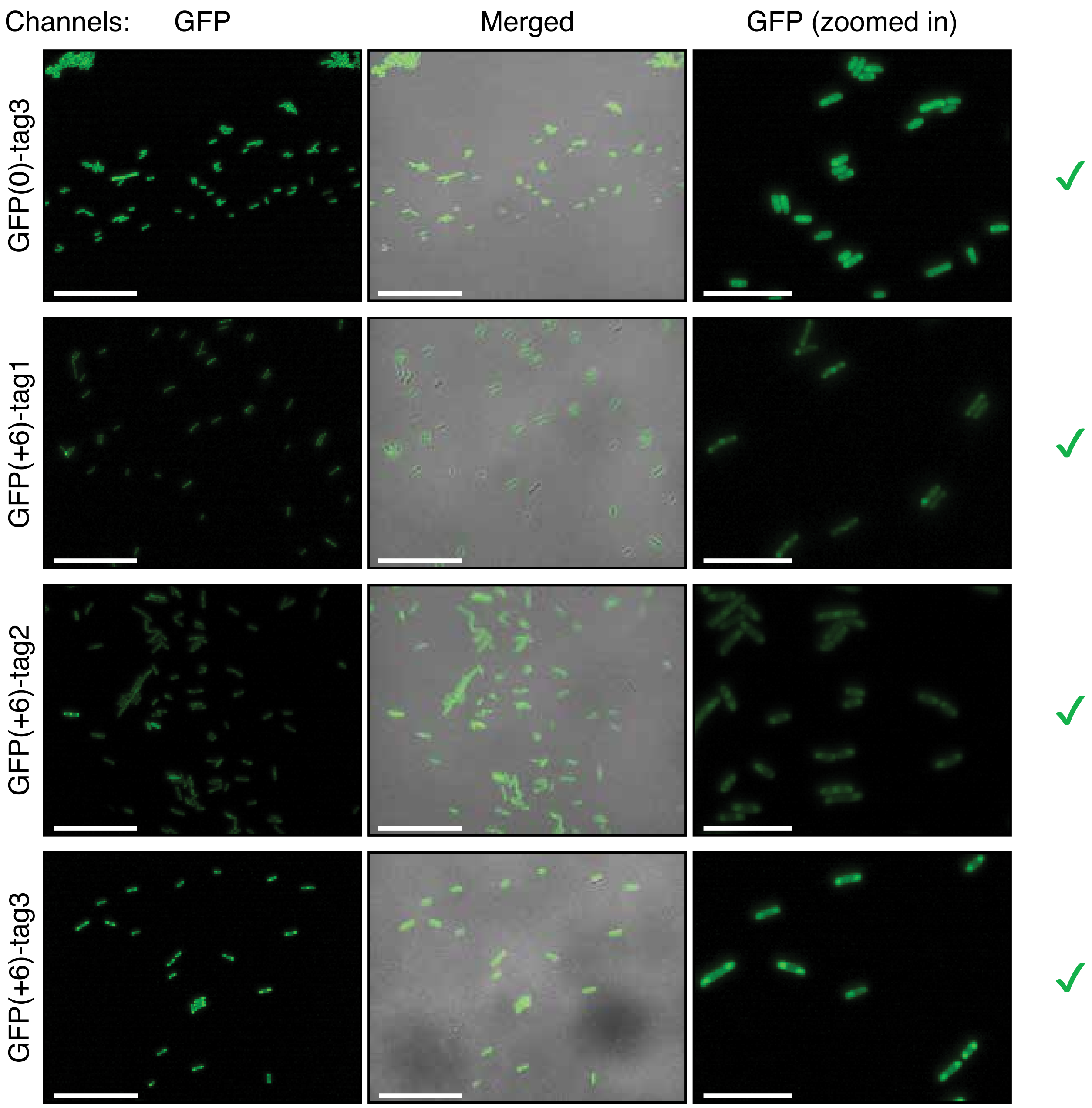

Supplementary Figure 3. Representative microscopy images of *E. coli* cells expressing each GFP variant at 4 h post-induction. GFP and merged (GFP and brightfield) images depict a population of cells for each variant. Scale bars are 25 μm for GFP (left) and merged (middle) images, and 10 μm for the zoomed-in (right) images. Green check marks indicate the presence of condensates and red "X"s indicate their absence. Similar results were observed in all the biological replicates (n=3) for each variant.

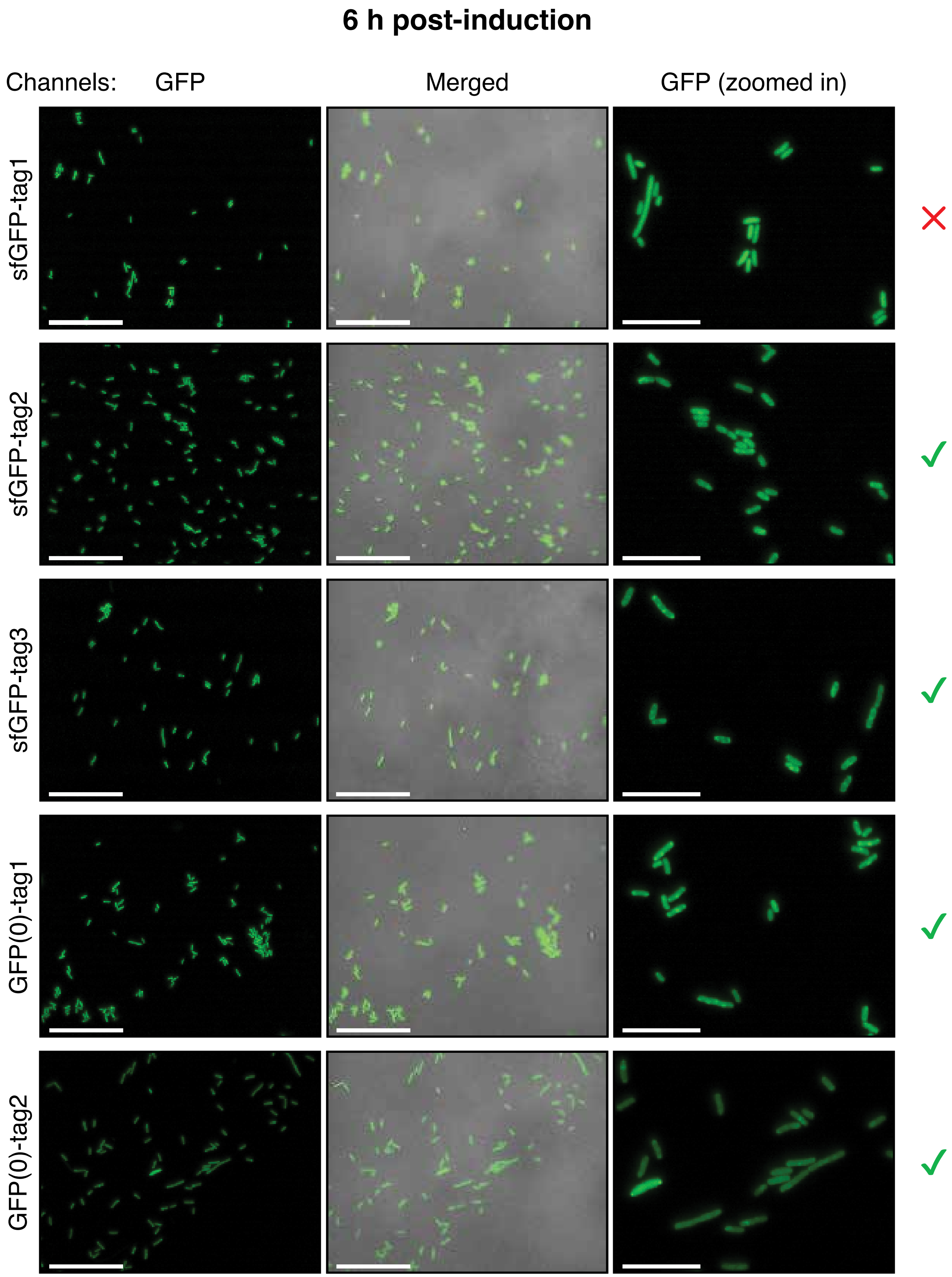
6 h post-induction
Channels:
GFP
Merged
GFP (zoomed in)
sfGFP-tag1
sfGFP-tag2
sfGFP-tag3
GFP(0)-tag1
GFP(0)-tag2

6 h post-induction (continued)

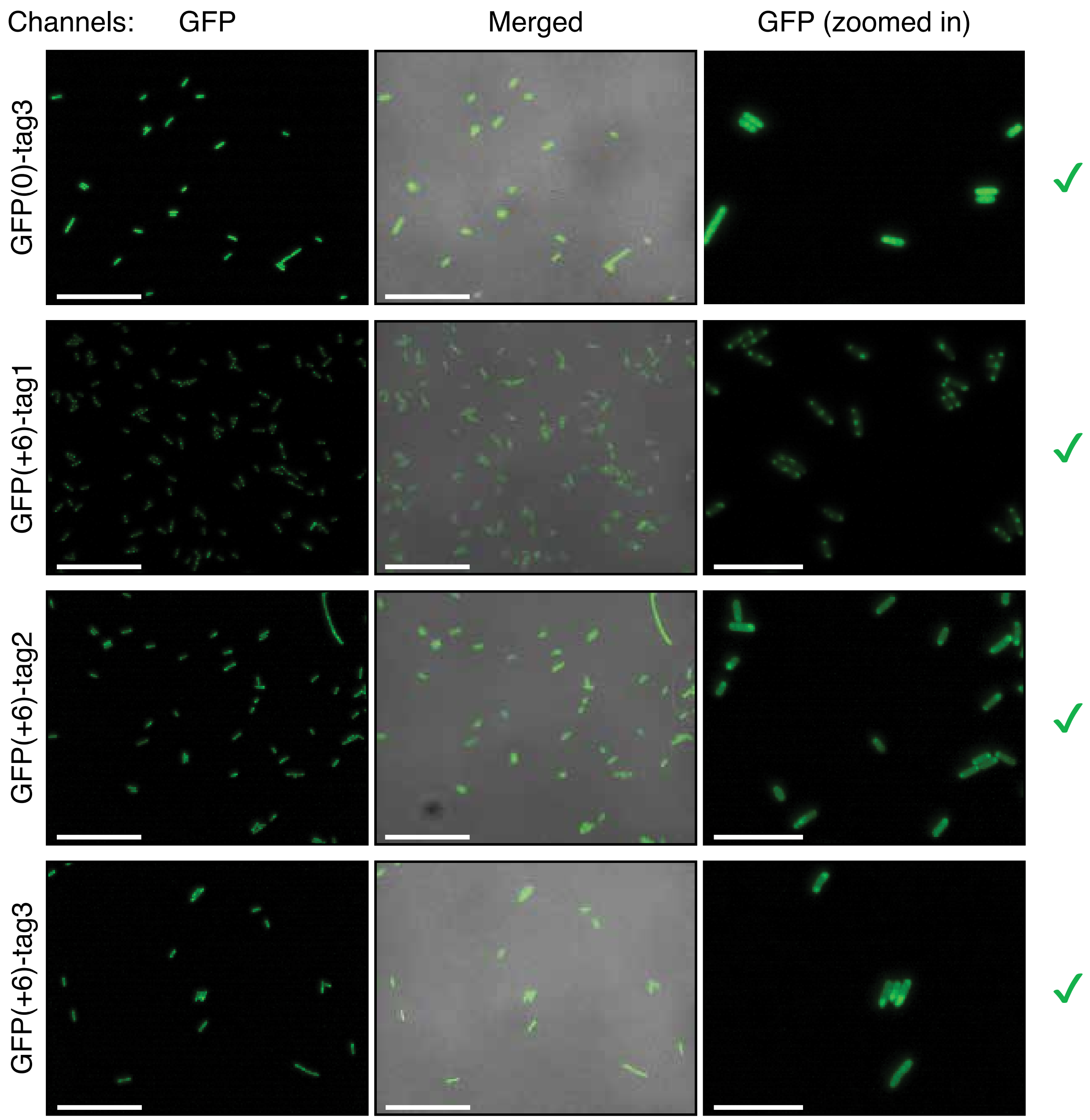

Supplementary Figure 4. Representative microscopy images of *E. coli* cells expressing each GFP variant at 6 h post-induction. GFP and merged (GFP and brightfield) images depict a population of cells for each variant. Scale bars are 25 μm for GFP (left) and merged (middle) images, and 10 μm for the zoomed-in (right) images. Green check marks indicate the presence of condensates and red "X"s indicate their absence. Similar results were observed in all the biological replicates (n=3) for each variant.

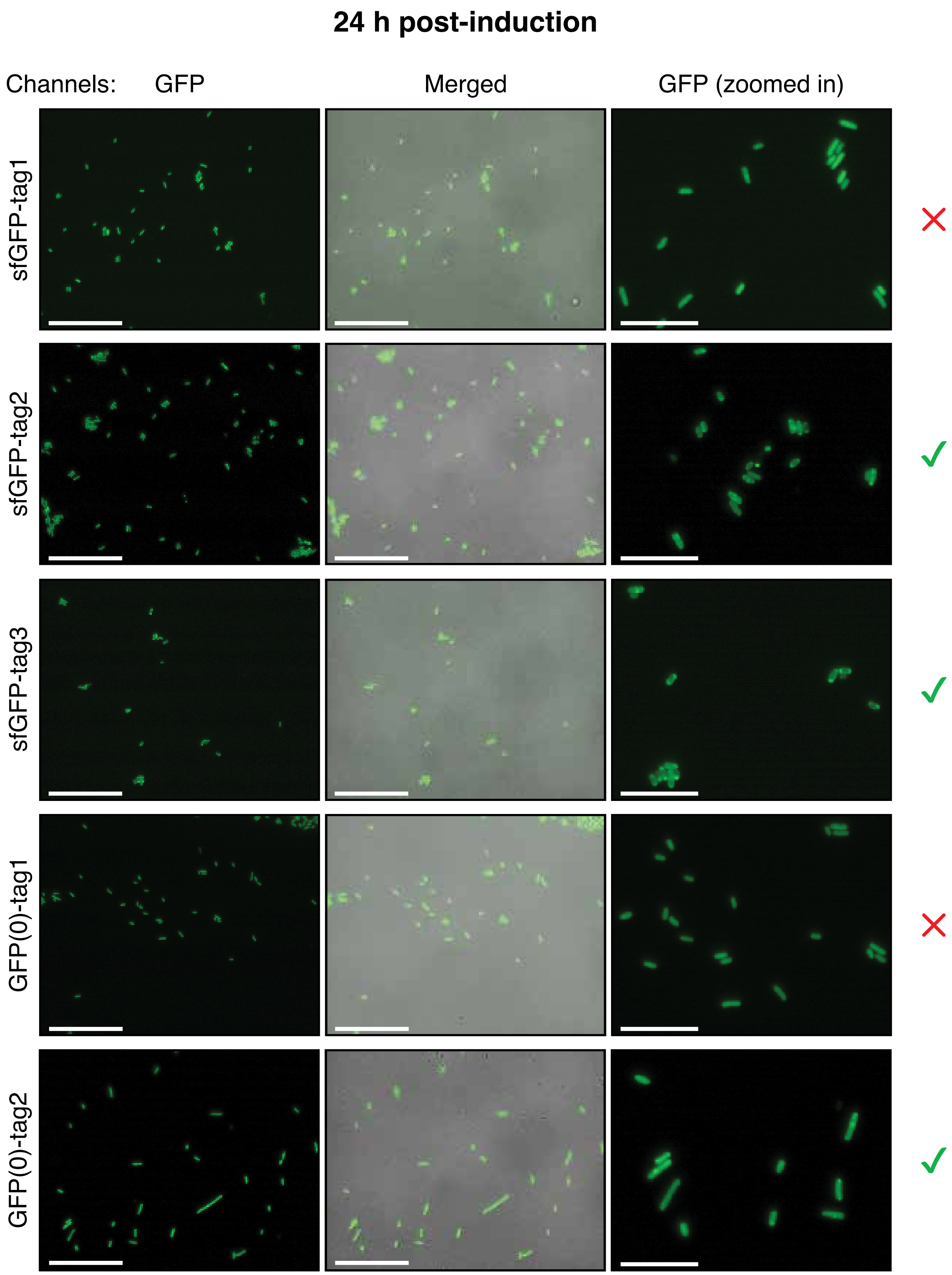
24 h post-induction
Channels:
GFP
Merged
GFP (zoomed in)
sfGFP-tag1
sfGFP-tag2
sfGFP-tag3
GFP(0)-tag1
GFP(0)-tag2

24 h post-induction (continued)

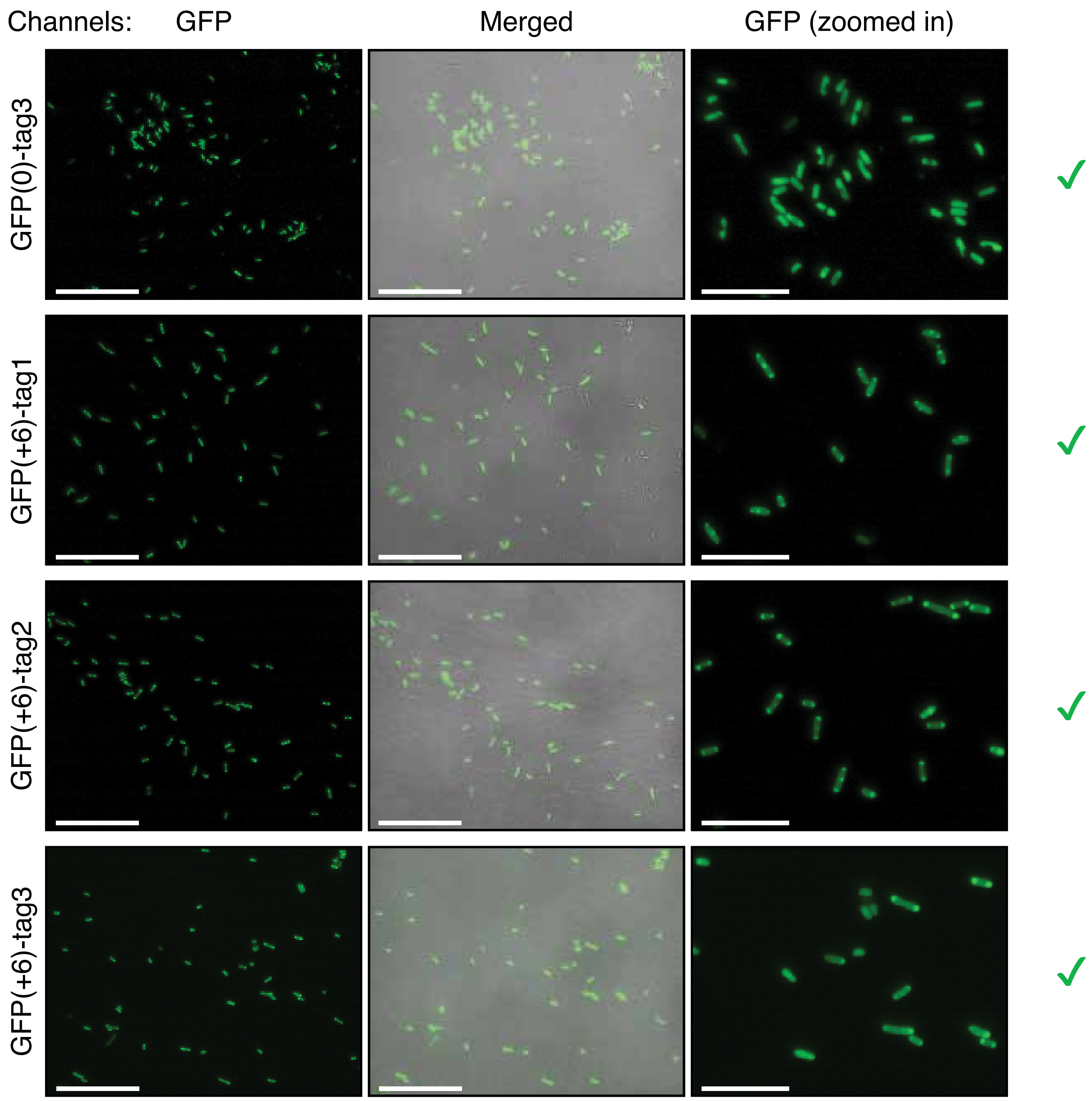

Supplementary Figure 5. Representative microscopy images of *E. coli* cells expressing each GFP variant at 24 h post-induction. GFP and merged (GFP and brightfield) images depict a population of cells for each variant. Scale bars are 25 μm for GFP (left) and merged (middle) images, and 10 μm for the zoomed-in (right) images. Green check marks indicate the presence of condensates and red "X"s indicate their absence. Similar results were observed in all the biological replicates (n=3) for each variant.

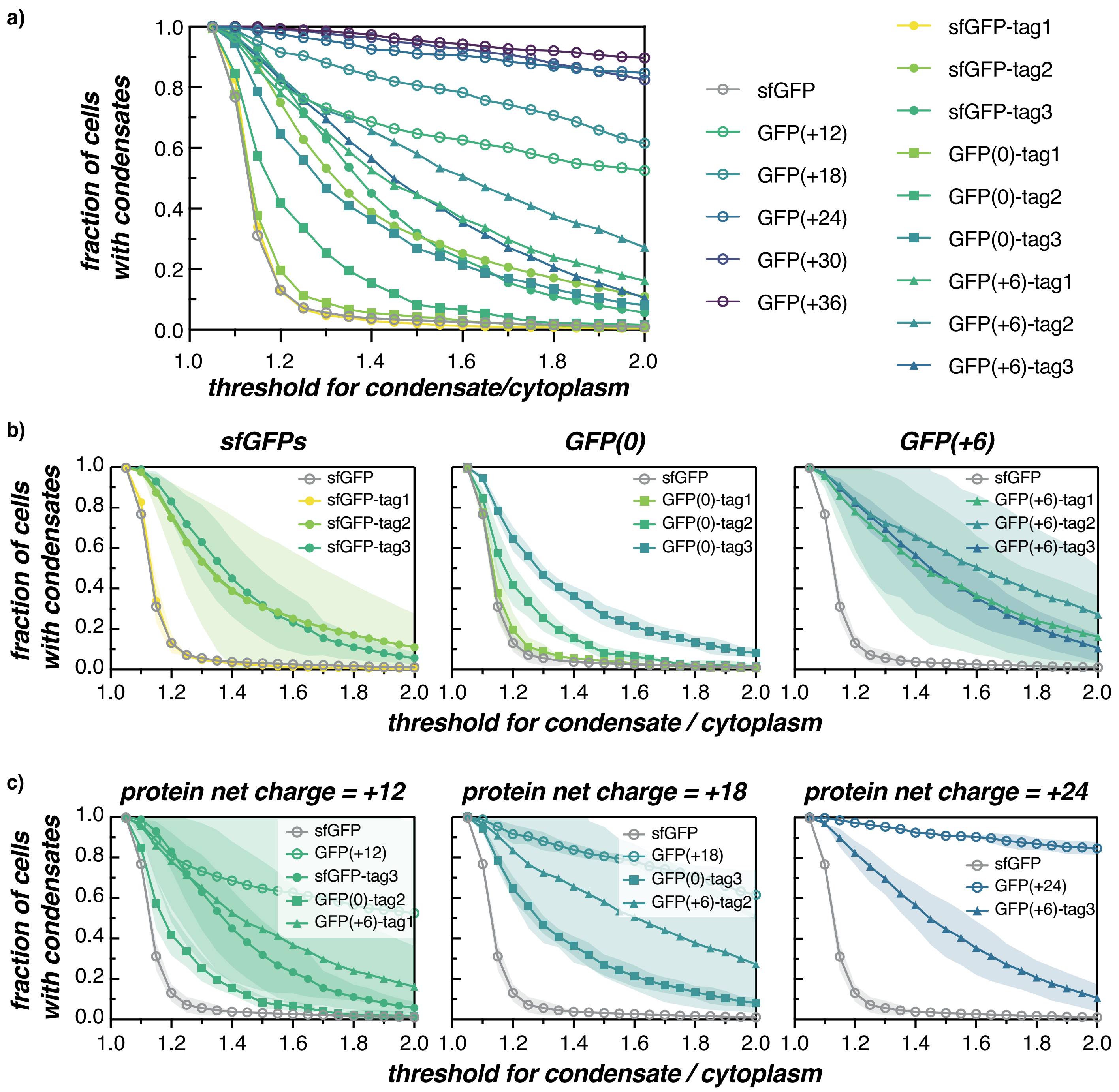

Supplementary Figure 6. Fraction of cells with condensates for select GFP isotropic and tagged variants at 24 h analyzed using various fluorescence intensity thresholds. The ratio of fluorescence intensity in the condensate to that in the cytoplasm was determined from microscopy images using a custom MATLAB script. (a) Average fraction of cells with condensates for select GFP variants (across 3 biological replicates) at 24 h post-induction remains unchanged for variants that form strong condensates (i.e., GFP(+36) and GFP(+30)) as the fluorescence intensity cutoff is increased. In contrast, variants that do not form condensates (i.e., sfGFP, GFP(0)-tag1) show a sharp drop in the average fraction of cells that levels off at a threshold of < 20%. Variants that form weak condensates show intermediate behaviors. (b) GFP variants shown in (a) binned by globular domain. (c) GFP variants shown in (a) binned by protein net charge. Standard deviations are depicted by the shaded regions.

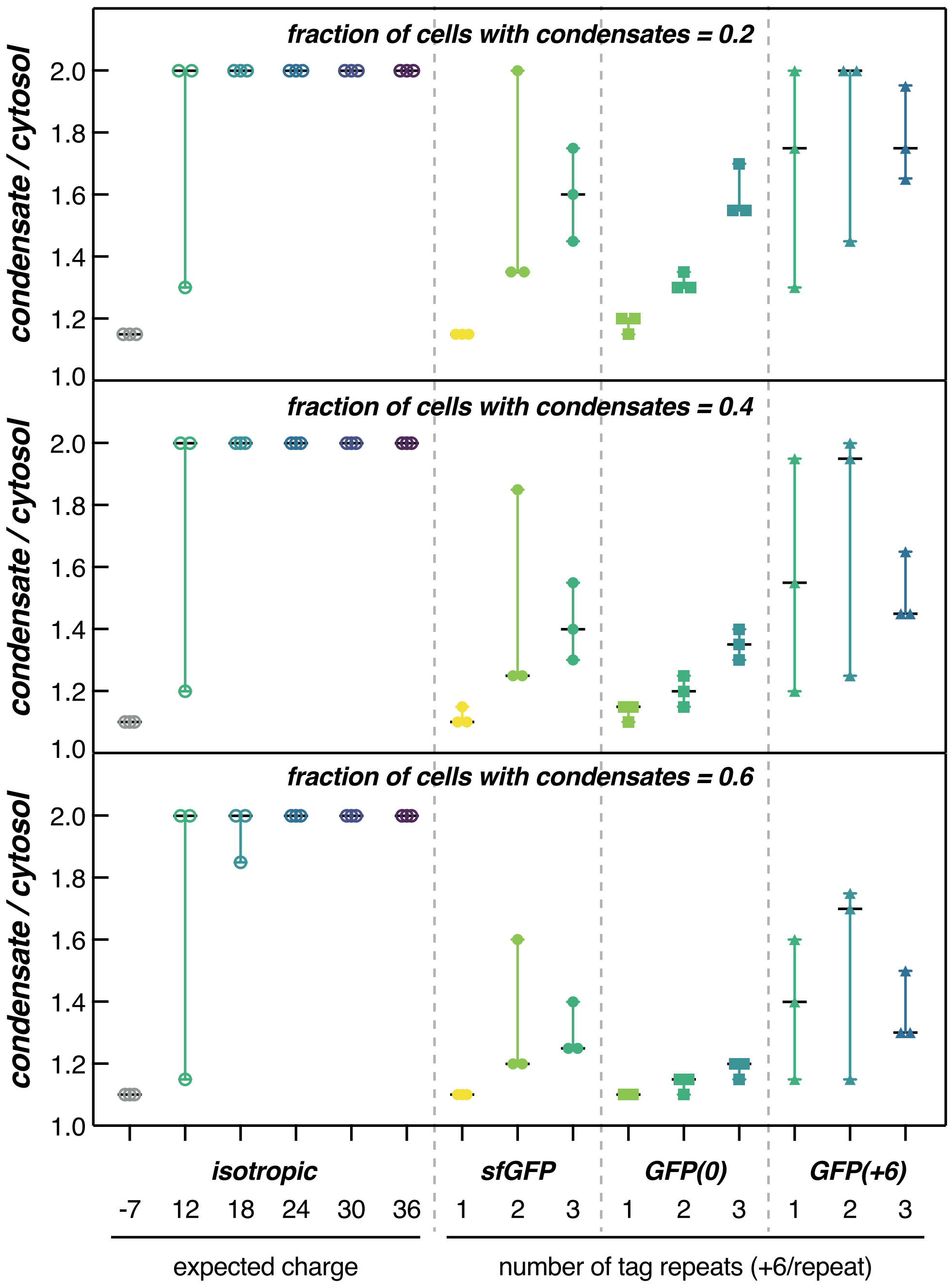

Supplementary Figure 7. GFP localization in cells at 24h post-induction. The ratio of fluorescence intensity in the condensate to that in the cytoplasm for cells expressing each GFP variant was obtained from microscopy images. The fraction of cells containing condensates was determined using a custom MATLAB script where the threshold value used to identify condensates was varied from 1.05 to 2.00 (see Supplementary Figure 6). The threshold value that identified the fraction of cells with condensates as at least 0.2 (top), 0.4 (middle) or 0.6 (bottom) was then plotted. The threshold value (y-axis) represents the approximate ratio of GFP fluorescence between the condensed phase and cytoplasm. Shown are three biological replicates for each GFP variant, the median, and interquartile ranges. For the isotropic GFP variants, the ratio of GFP in the condensate to that in the cytoplasm did not change, indicating the enrichment of GFP in the condensed phase was greater than two-fold. The tagged GFP variants demonstrated a slight decrease in the condensate/cytoplasm ratio as the required fraction of cells containing condensates was increased; however, general trends observed between the tagged variants remained the same.

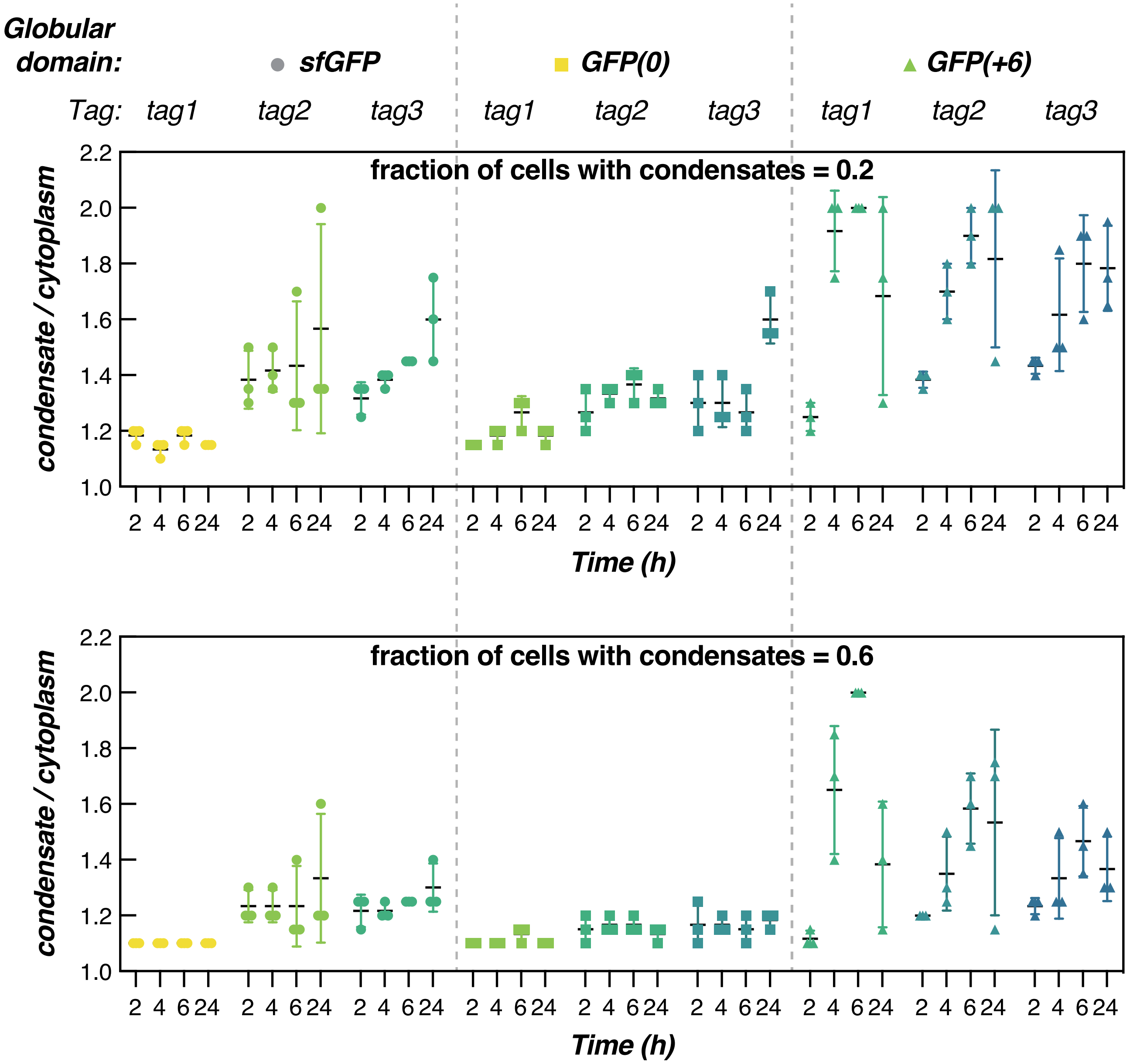

Supplementary Figure 8. Relative GFP fluorescence in the condensate and cytoplasm over time in cells expressing tagged GFP variants. Cells expressing each tagged GFP variant were imaged at various time points (2, 4, 6, and 24 h) after induction with IPTG to monitor condensate formation. The fraction of cells containing condensates was determined using a custom MATLAB script where the threshold value used to identify condensates was varied from 1.05 to 2.00 (see Supplementary Figure 6). The threshold value that identified the fraction of cells with condensates as at least 0.2 (top) or 0.6 (bottom) was then plotted. The threshold value (y-axis) represents the approximate ratio of GFP fluorescence between the condensed phase and cytoplasm. Similar to the trends observed for the tagged GFP variants at 24 h, proteins with the cationic globular domain demonstrated a higher condensate / cytoplasm ratio at earlier timepoints. The average and standard deviation of three biological replicates are plotted for each variant.

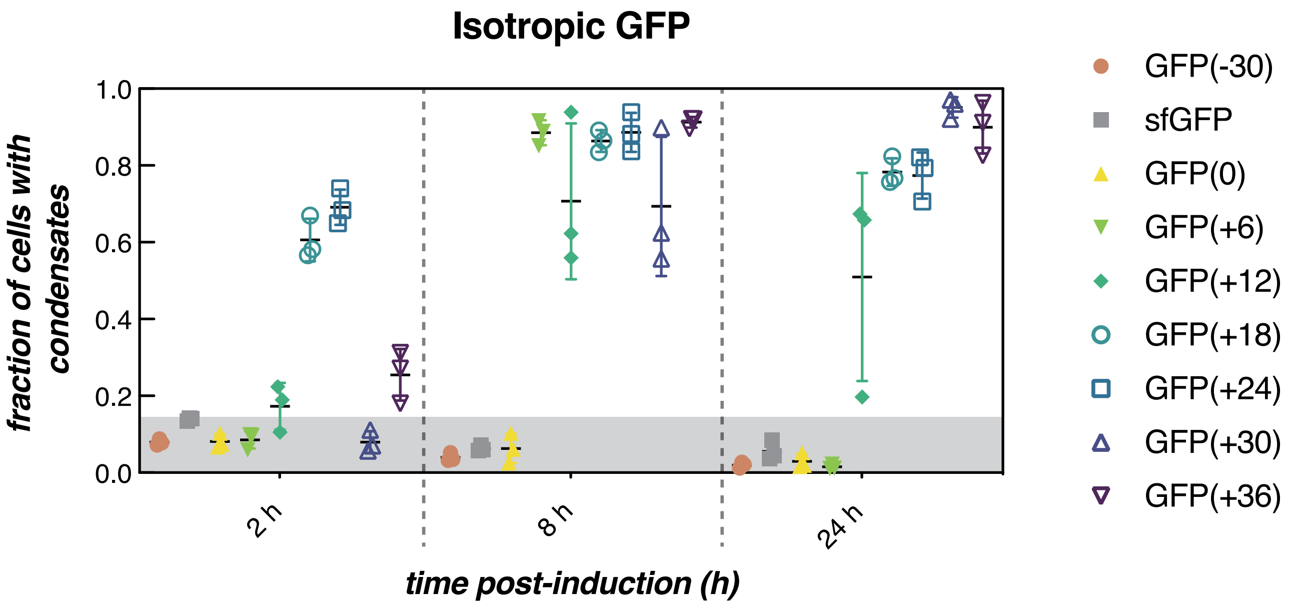

Supplementary Figure 9. Supercationic isotropic GFP variants form condensates over time. Image analysis further confirms intracellular phase separation in cells that express GFP variants with sufficient positive charge. As noted preivously, GFP variants with net charge ≥ +12 form and maintain condensates as protein is expressed post induction.[5] GFP(+6) undergoes reversible intracellular phase separation. The fracion of cells with condensates for three biological replicates (data points), the average (black line) and standard deviation (error bar) are plotted for each variant.

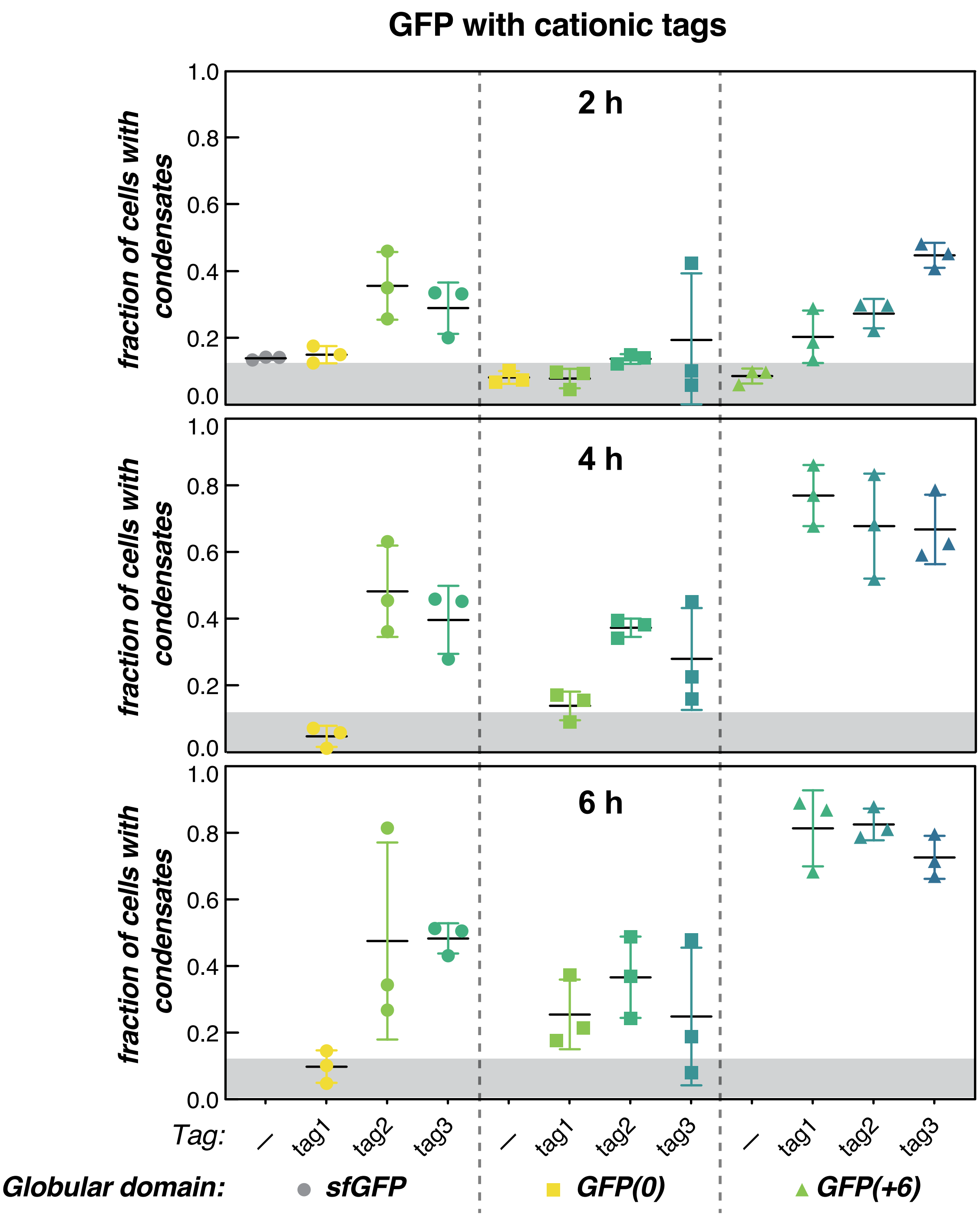

Supplementary Figure 10. Tagged GFP variants can form condensates over time. Quantification of microscopy images (n ≥ 416 total cells for each variant) at 2, 4, and 6 h post-induction. Data for isotropic variants were collected at 2 h, but not at 4 or 6 h [5]. The fraction of cells that contained detectable condensates were plotted for each variant. Generally, variants with a fraction < 0.11 did not form observable condensates. The average and standard deviation of three biological replicates are plotted for each variant.

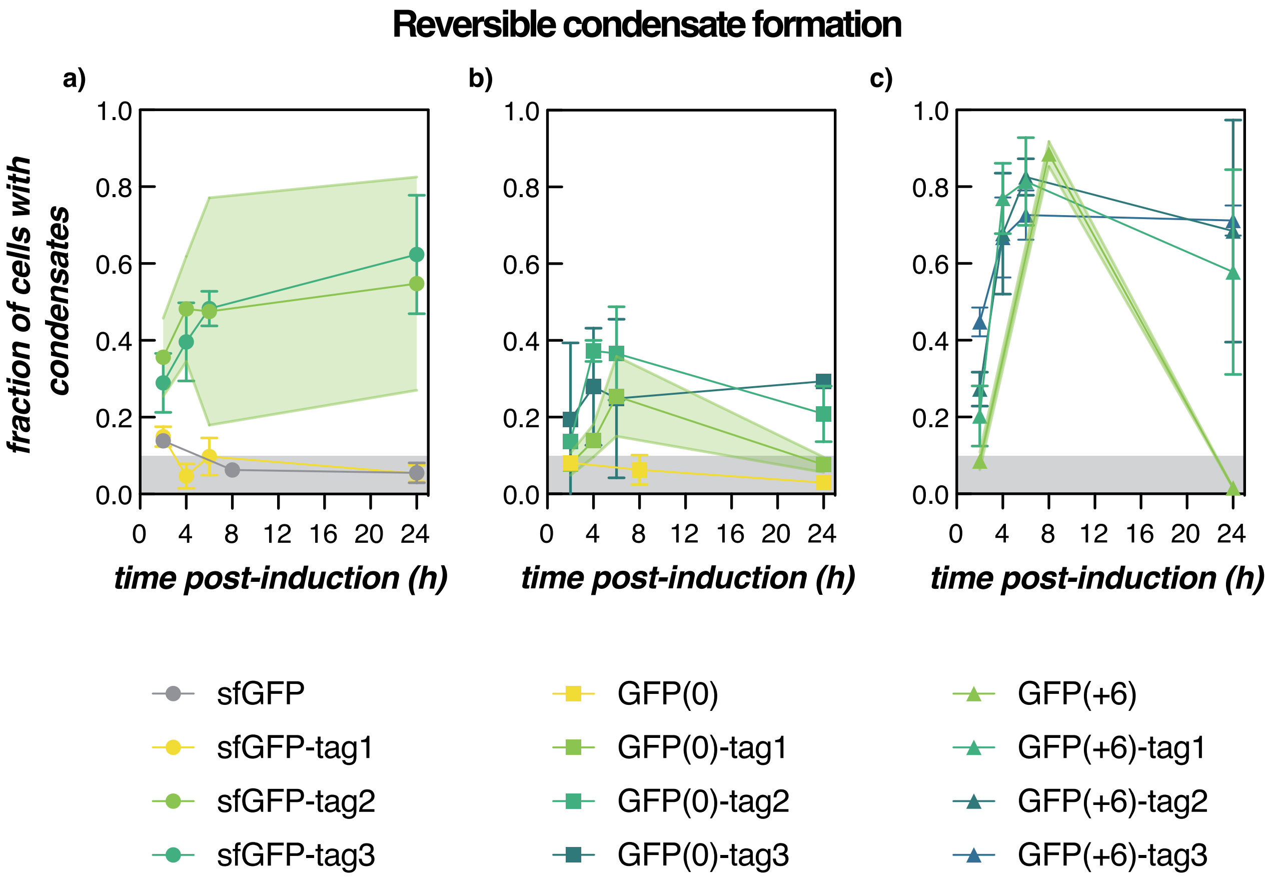

Supplementary Figure 11. Reversible condensate formation. The results from Supplementary Fig. 10 were replotted as a function of time instead of by protein variant, (a) sfGFP variants, (b) GFP(0) variants, and (c) GFP(+6) variants. The gray shaded region represents an approximate threshold, below which cells did not form observable condensates. Condensate formation was observed for cells expressing GFP(0)-tag1 (b) and GFP(+6) (c) at intermediate time points (6 h and 8 h, respectively). However, these condensates disappeared at 24 h, suggesting that condensate formation is reversible for these variants. The average and standard deviation of three biological replicates are plotted for each variant.

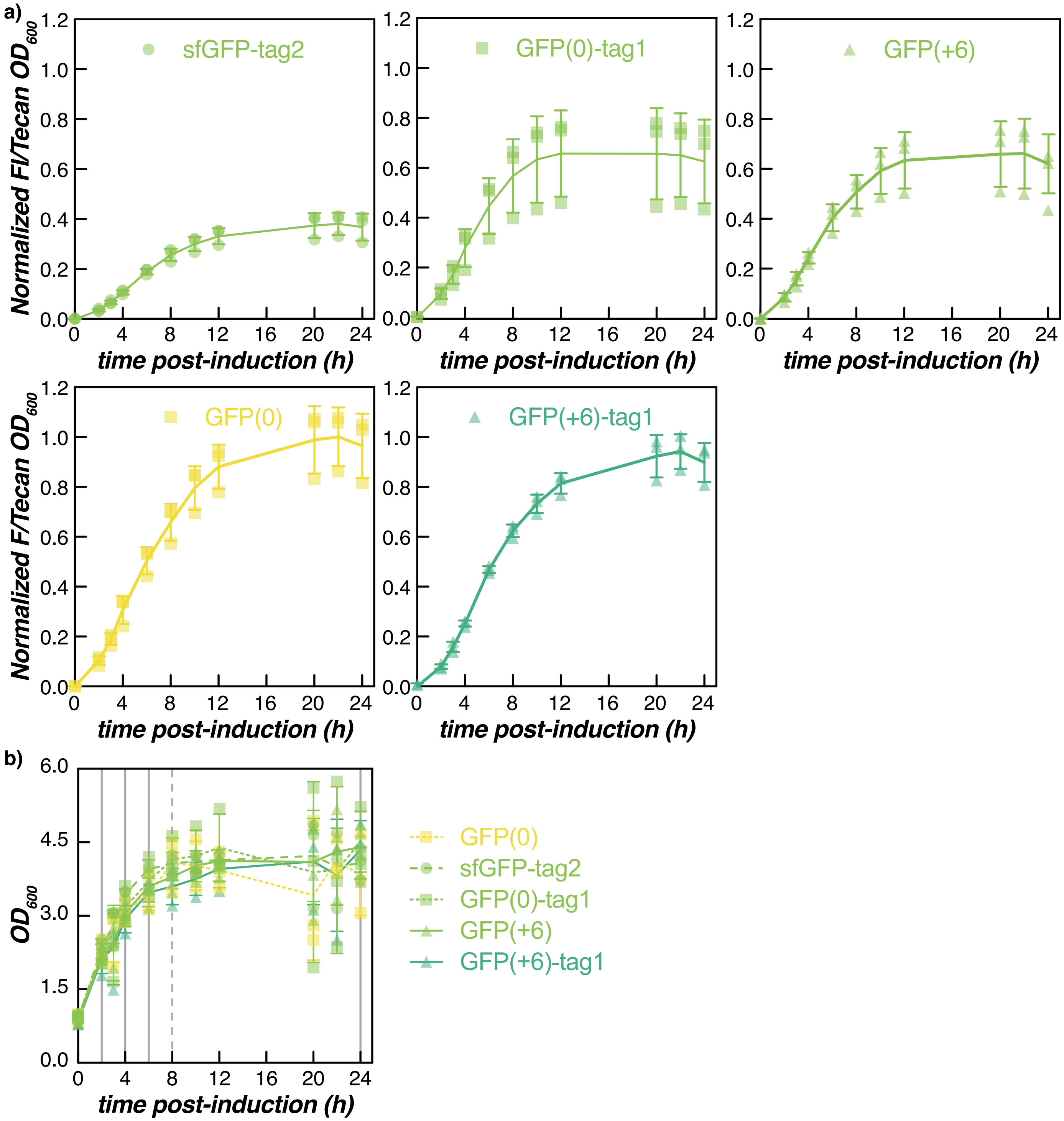

Supplementary Figure 12. Cell growth and protein expression. (a) The ratio of GFP fluorescence intensity to cell density was plotted on separate graphs for select GFP variants. This ratio was used as a proxy for the intracellular GFP concentration. The intracellular concentrations of like-charged GFP(+6) and GFP(0)-tag1 were similar, providing one explanation for how these two strains were able to traverse the phase boundary. Plotted are three individual biological replicates, average and standard deviation of the normalized fluorescence intensity/Tecan OD ratio for each variant. This ratio was calculated by averaging across three biological replicates and then normalizing to the highest average fluorescence intensity/Tecan OD ratio amongst all GFP variants (GFP(0) at t = 22 h). (b) The optical density was also measured in cuvettes for these same cultures. Plotted are the individual biological replicates, average and standard deviation of the cuvette OD_{600} for each variant in (a). Solid grey lines mark the times post-induction that tagged variants were imaged (2, 4, 6, and 24 h). The dashed grey line indicates the time post induction that the isotropically supercharged variant was imaged (8 h).

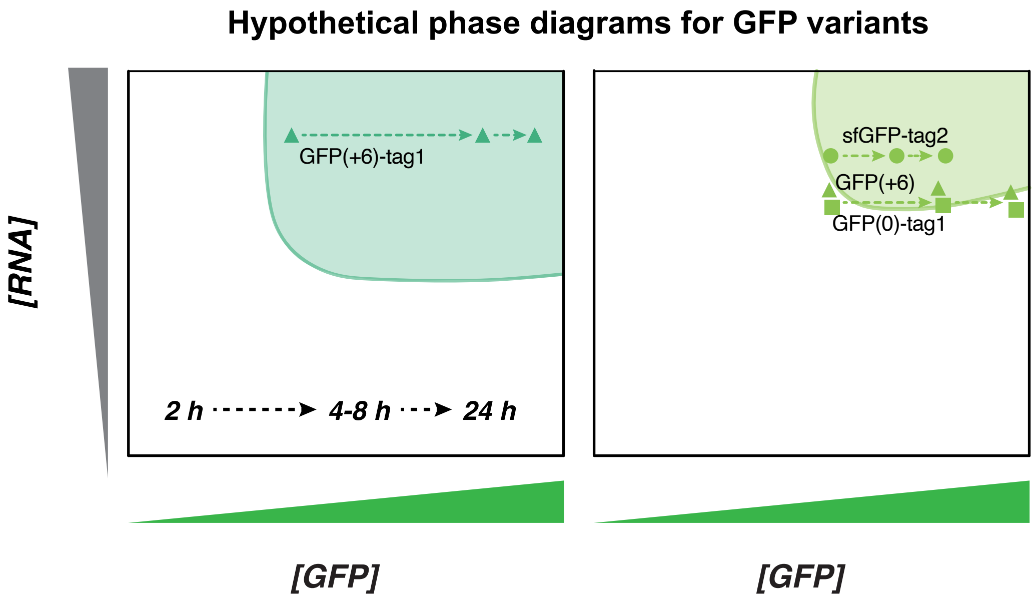

Supplementary Figure 13. Traversing the physiological phase boundary. This model phase diagram depicts how GFP(+6) and GFP(0)-tag1 may undergo reversible condensate formation (right). GFP(+6)-tag1 (left) has sufficient charge to remain within the two phase region despite large changes in intracelluar GFP concentration. In contrast, for GFP variants with lower net charge (e.g. +6) condensates were observed at some time points but not all. For these variants, the disappearance of condensates was correlated with changes in the intracellular GFP concentration, as monitored by average fluorescence/OD. We hypothesize that as the net protein charge decreases there are fewer biomolecule (GFP, RNA) compositions that lie within the two phase region, enabling GFP(+6) and GFP(0)-tag1 to traverse the phase boundary twice as the protein concentration increases over time.

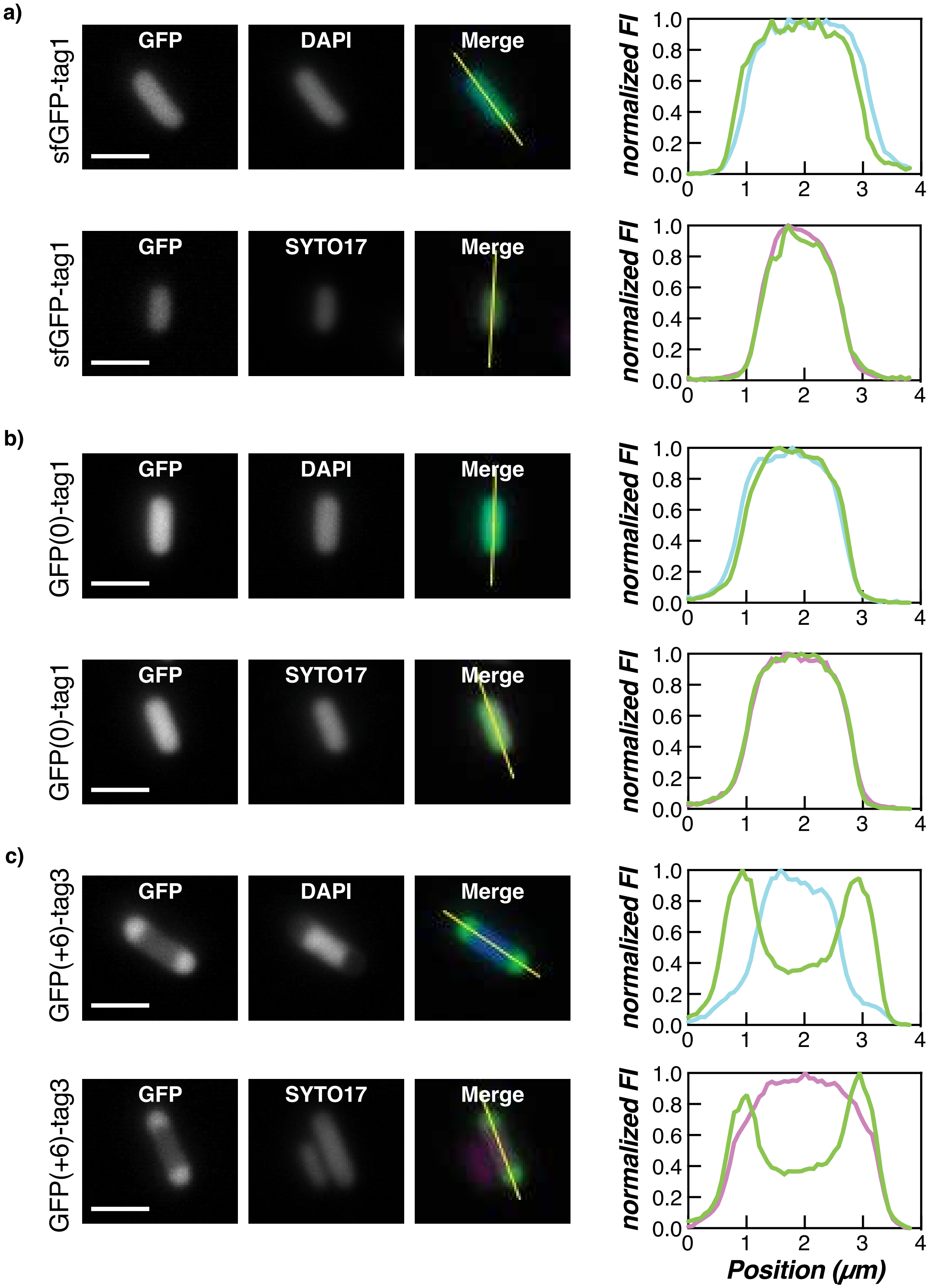

Supplementary Figure 14. Nucleic acid staining of cells at 24 h post-induction. DAPI (stains DNA) and SYTO17 (stains DNA and RNA) staining of cells expressing (a) sfGFP-tag1, (b) GFP(0)-tag1, and (c) GFP(+6)-tag3 at 24 h post-induction. Briefly, a line cut was drawn across representative cells and fluorescence intensities were measured along the line cut in each channel. Depicted are normalized fluorescence intensities at each position along the cell. DAPI is excluded in GFP(+6)-tag3 condensates while SYTO17 is present throughout the cell. This indicates that the condensates exclude DNA but allow for RNA partitioning . Scale bar is 2 μm. Microscopy images and analysis were obtained for cells in one biological replicate.

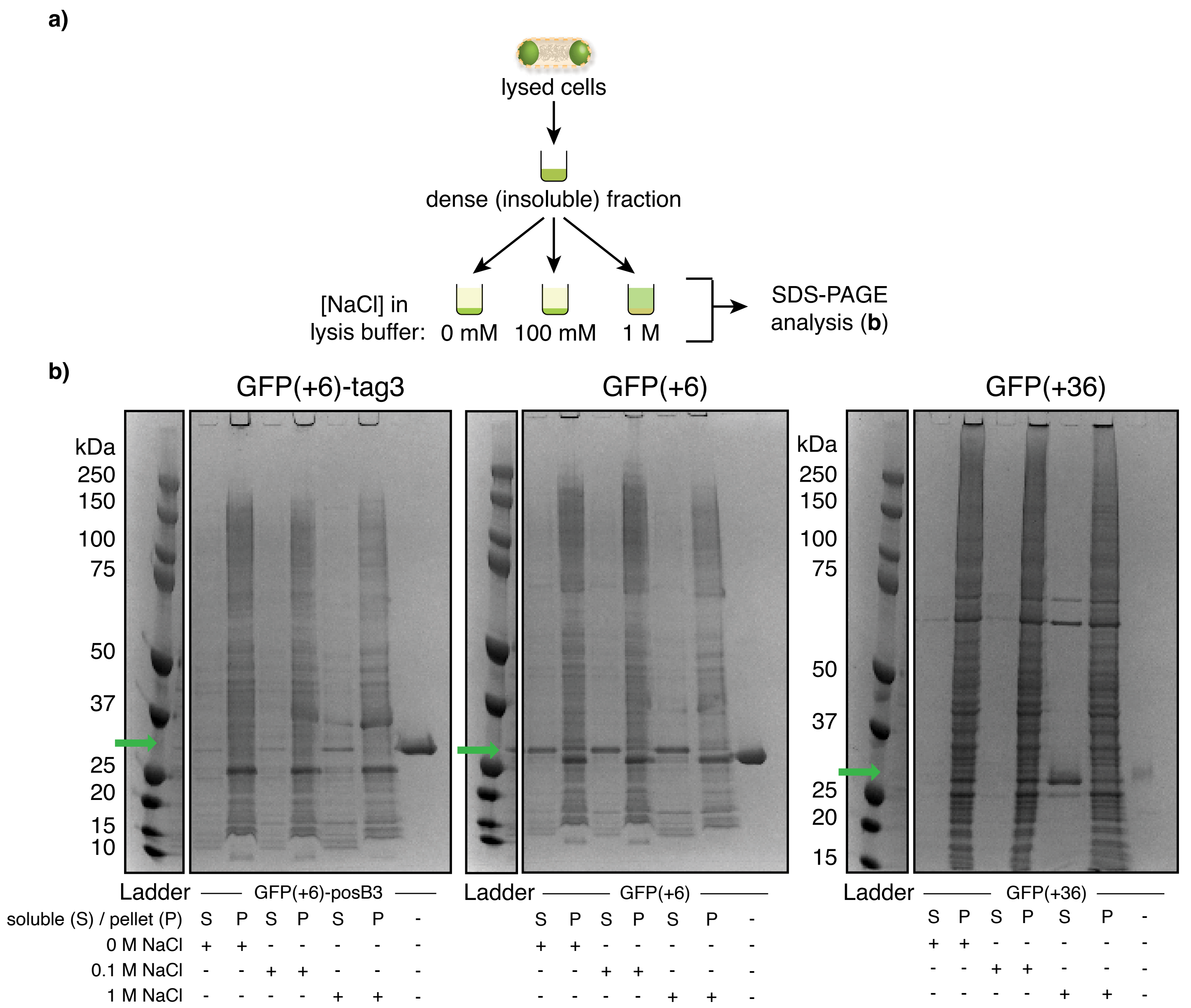

Supplementary Figure 15. Solubility of GFP(+6)-tag3, GFP(+6), and GFP(+36) in buffers of varied ionic strength. (a) Schematic depicting the extraction of GFP from the cell as previously reported [5]. Briefly, lysed cells were separated into soluble and insoluble fractions by centrifugation. The insoluble fraction was then washed and resuspended in a buffer containing different concentrations of NaCl. These samples were separated into their soluble and insoluble fractions, which were collected and analyzed on SDS-PAGE gels. (b) SDS-PAGE gels depicting similarities in solubility between the GFP(+6)-tagged variant and a coacervate-forming isotropic GFP variant, GFP(+36). "S" refers to the soluble fraction, and "P" refers to the pellet. "+" indicates treatment with buffers of varying NaCl concentration (0 M, 0.1 M, or 1 M). Precision Plus Protein Dual Color standard (Bio-rad) was loaded in lane 1 for all gels. Lanes 2-7 depict different buffer treatments of cell lysates and lane 8 corresponds to the respective purified GFP. The ladders shown were loaded onto the same gels as the individual GFP variants; however, irrelevant lanes were excluded for clarity. The band corresponding to GFP is indicated by the green arrows. All gels depicted here originated from the same experiment and were processed in parallel. Similar results were observed for two replicates.

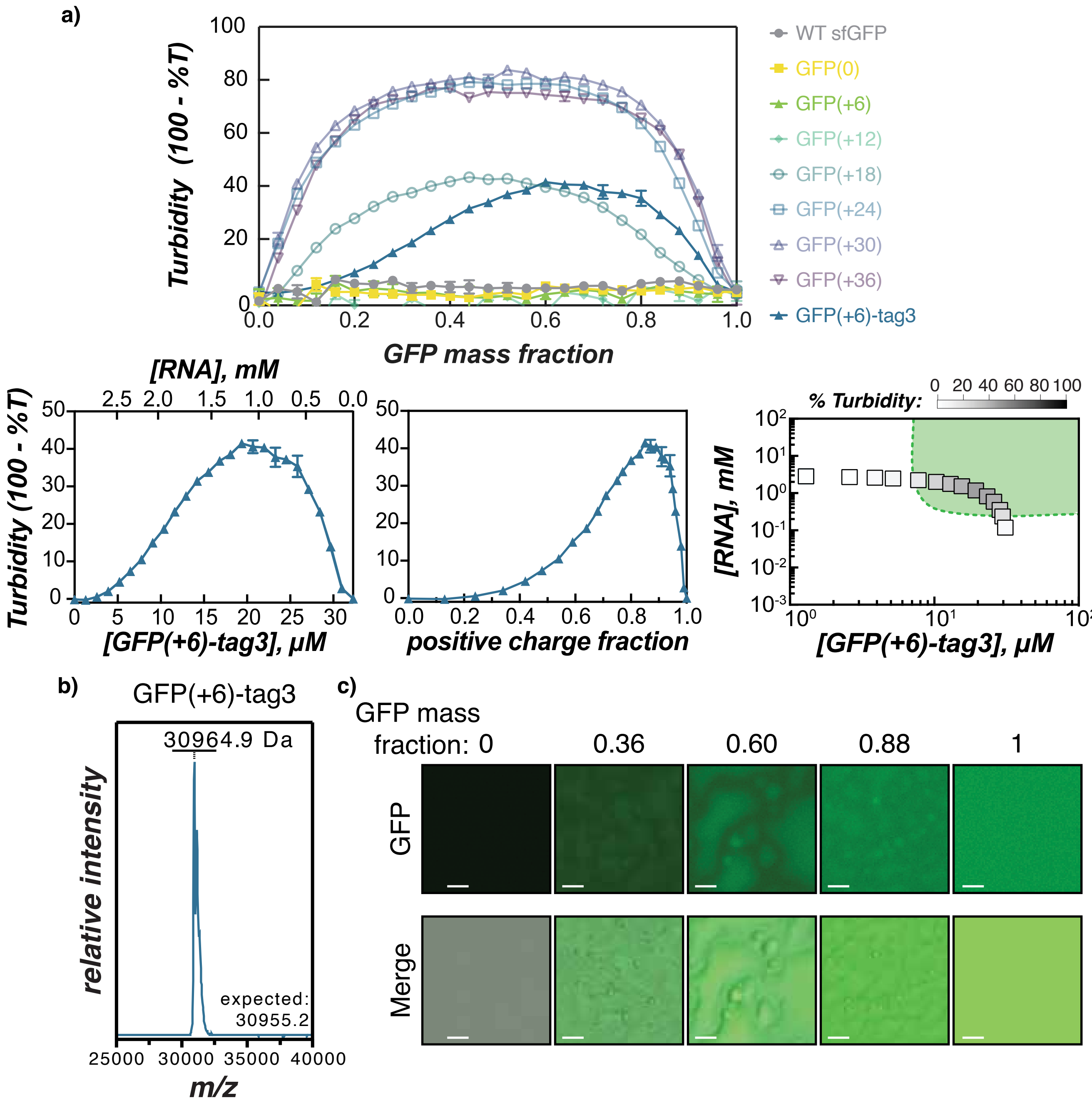

Supplementary Figure 16. *In vitro* complex coacervation of RNA with GFP(+6)-tag3. (a) Turbidity assay of supercharged cationic GFP variants with total RNA from torula yeast as reported previously [5]. (Top) The turbidity of the tagged variant falls between the untagged control, GFP(+6), and the isotropic protein with equivalent charge, GFP(+24). Turbidity values were plotted between -5 and 100% as turbdity values below 0 were an artifact of background subtraction (buffer blank). (Bottom) RNA concentration is plotted as the concentration of nucleotide monomers as total RNA is heterogeneous in length. The green shading in the phase diagram represents an approximated two phase region drawn based on turbidity values. The average and standard deviation of three technical replicates are plotted for each variant. (b) MALDI-TOF spectrum of purified GFP(+6)-tag3. The expected molecular weight was calculated based on the primary amino acid sequence without the methionine from the start codon. (c) Microscopy images of GFP(+6)-tag3/RNA mixtures at 1 mg/mL total macromolecule concentration show individual droplets, coalescence, and wetting at the surface. GFP (top) and merged (bottom) channels are depicted at the specified GFP mass fractions. Mixtures were prepared in a buffer that mimics the intracellular ion concentration in *E. coli* (70 mM K_2HPO_4, 60 mM KCl, 40 mM NaCl, pH 7.4). Scale bar is 10 μM. Microscopy images for one replicate were captured.

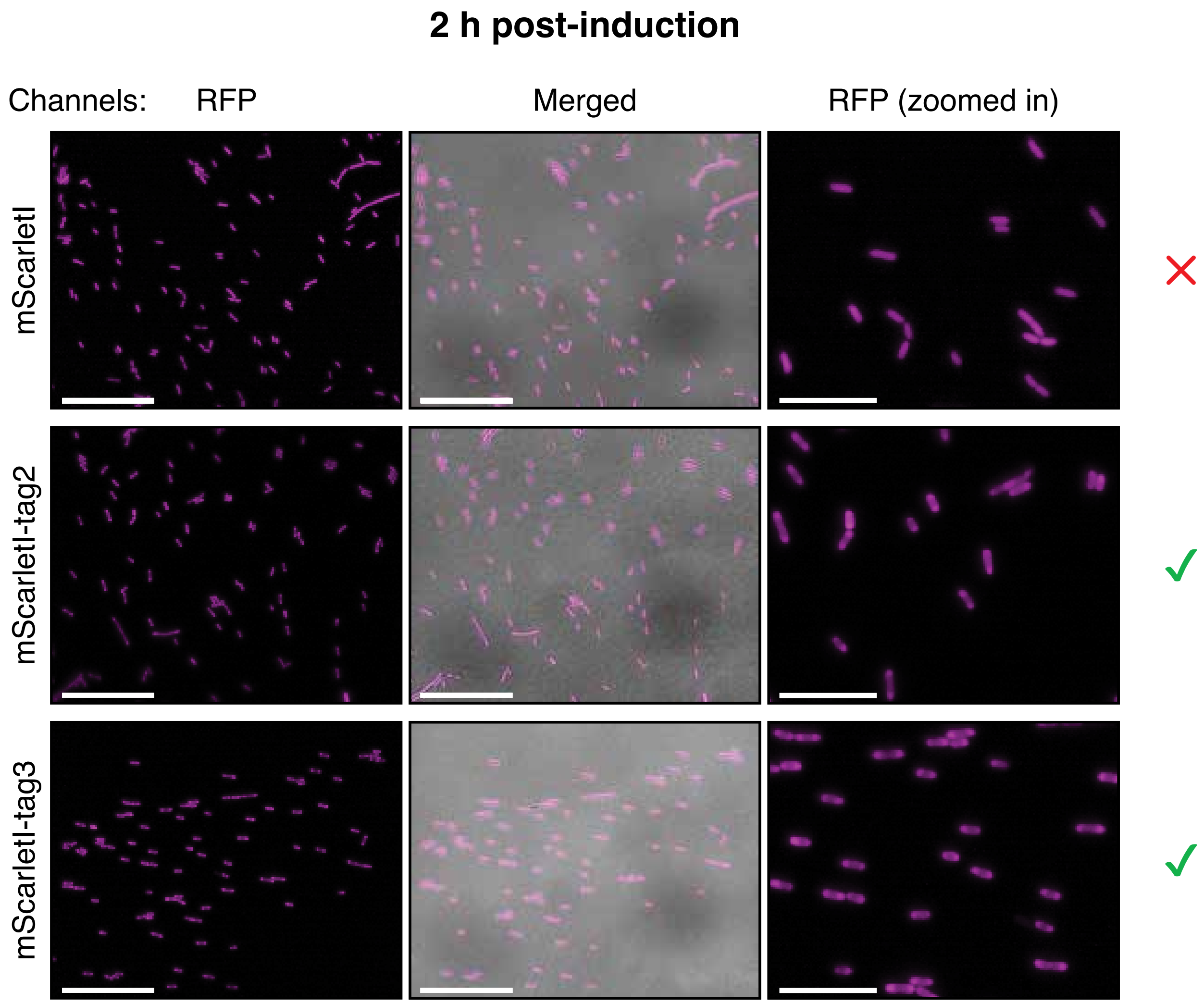

Supplementary Figure 17. Representative microscopy images of *E. coli* cells expressing each mScarletI variant at 2 h post-induction. TexasRed (RFP) and and merged (TexasRed and bright-field) channels depict a population of cells for each variant. Scale bars are 25 μm for RFP (left) and merged (middle) images, and 10 μm for the zoomed-in (right) images. Green check marks indicate the presence of condensates and red “X”s indicate their absence. Similar results were observed in all the biological replicates (n=3) for each variant.

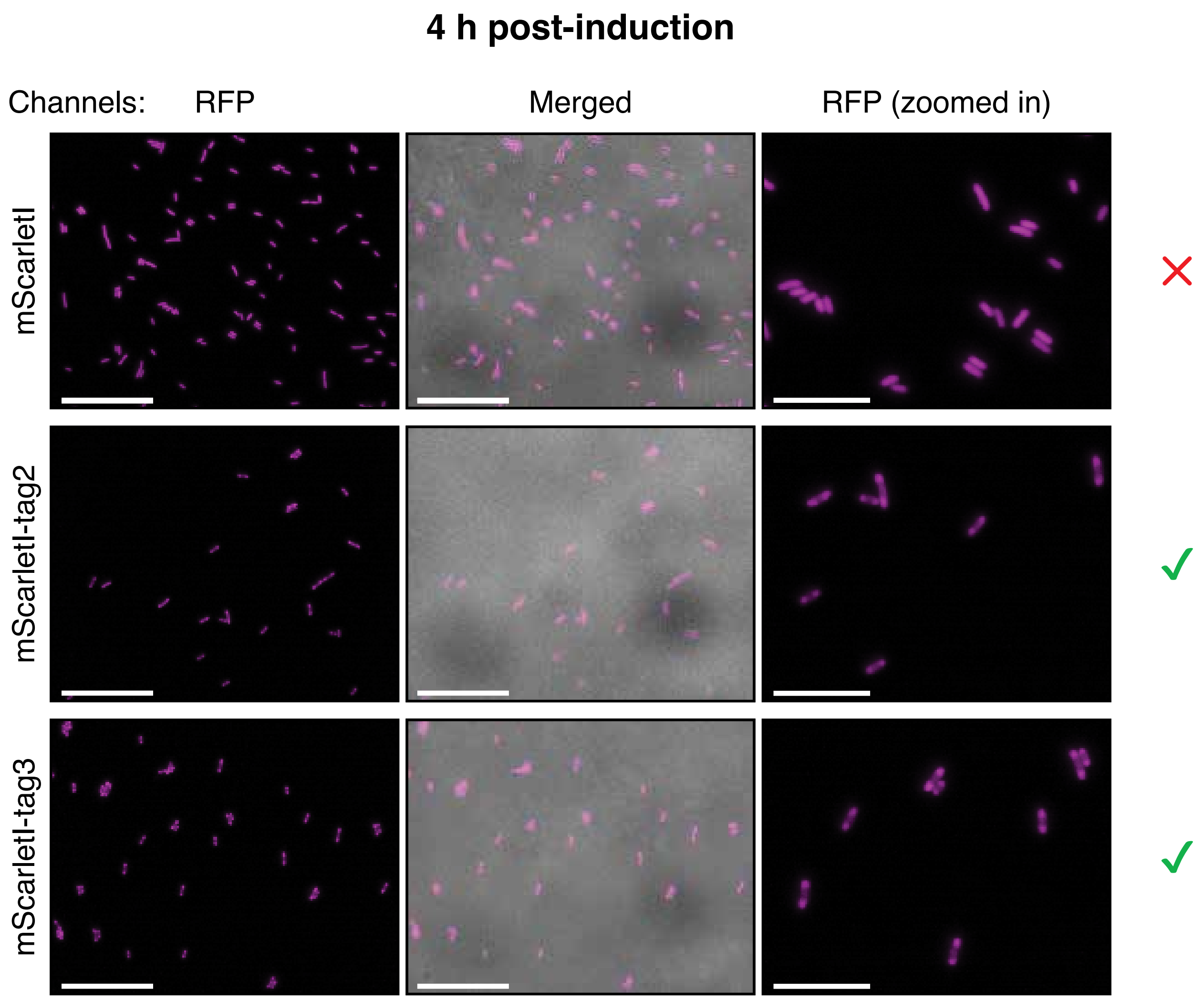

Supplementary Figure 18. Representative microscopy images of *E. coli* cells expressing each mScarletI variant at 4 h post-induction. TexasRed (RFP) and and merged (TexasRed and bright-field) channels depict a population of cells for each variant. Scale bars are 25 μm for RFP (left) and merged (middle) images, and 10 μm for the zoomed-in (right) images. Green check marks indicate the presence of condensates and red "X"s indicate their absence. Similar results were observed in all the biological replicates (n=3) for each variant.

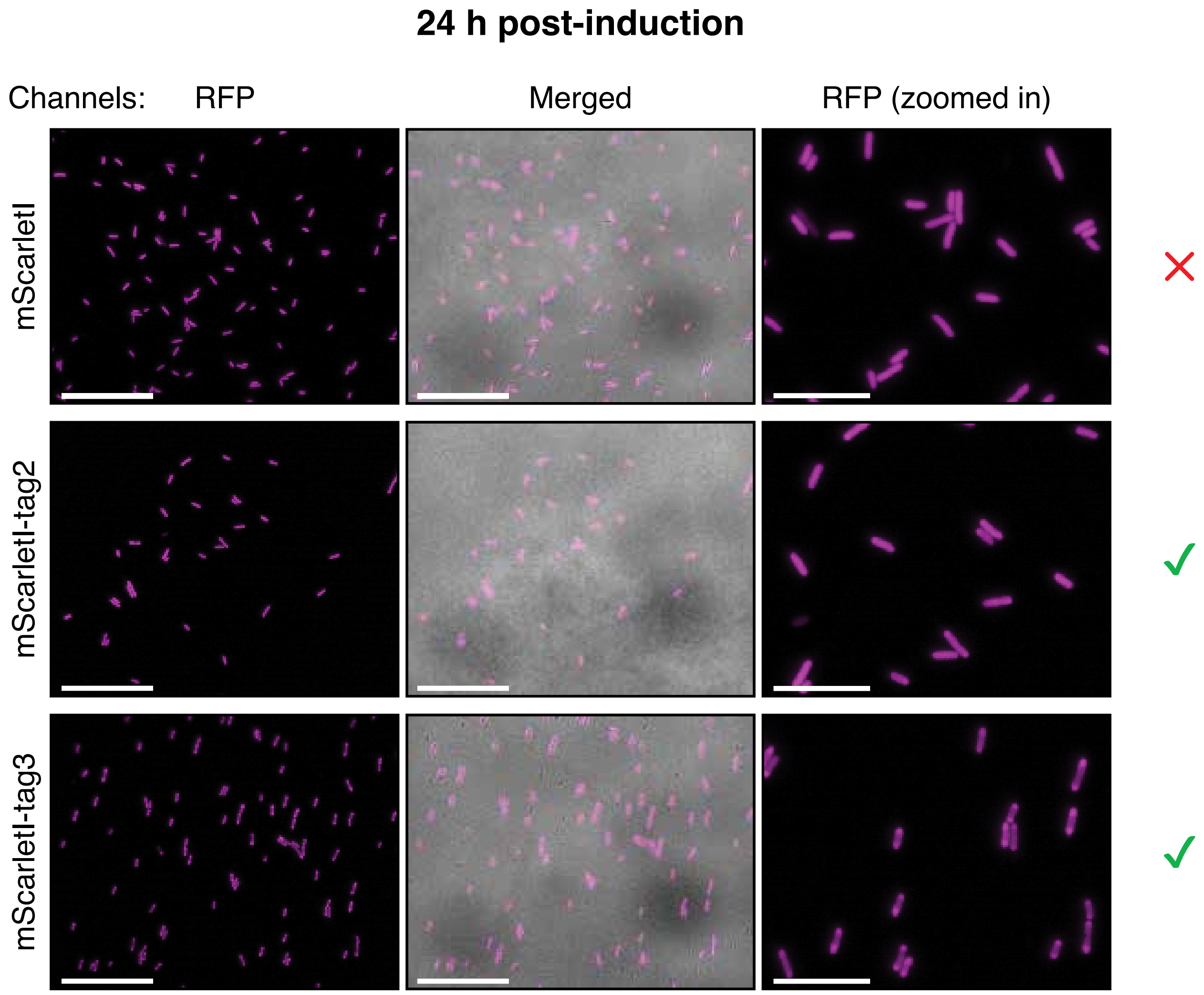

Supplementary Figure 19. Representative microscopy images of *E. coli* cells expressing each mScarletI variant at 24 h post-induction. TexasRed (RFP) and and merged (TexasRed and bright-field) channels depict a population of cells for each variant. Scale bars are 25 μm for RFP (left) and merged (middle) images, and 10 μm for the zoomed-in (right) images. Green check marks indicate the presence of condensates and red "X"s indicate their absence. Similar results were observed in all the biological replicates (n=3) for each variant.

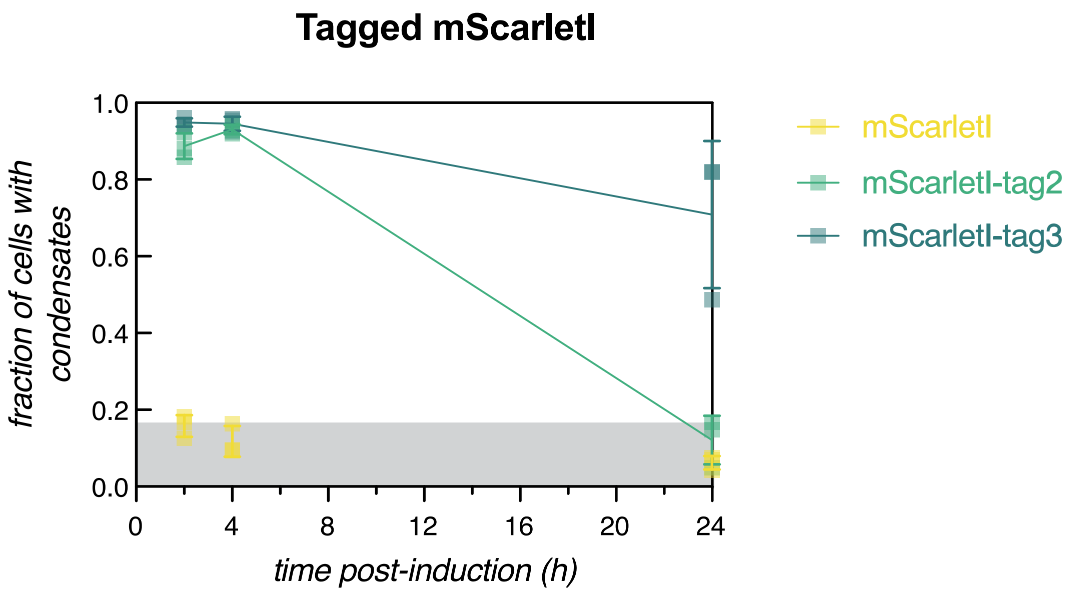

Supplementary Figure 20. Supercationic mScarletI variants form condensates over time. Quantificaition of microscopy images taken at 2, 4, and 24 h post-induction. The fraction of cells that contained detectable condensates were plotted for each variant as a function of time post-induction. This analysis further confirmed intracellular phase separation in cells that expressed mScarletI variants with sufficient positive charge. The average and standard deviation of three biological replicates are plotted for each variant along with the individual biological replicates.

24 h post-induction

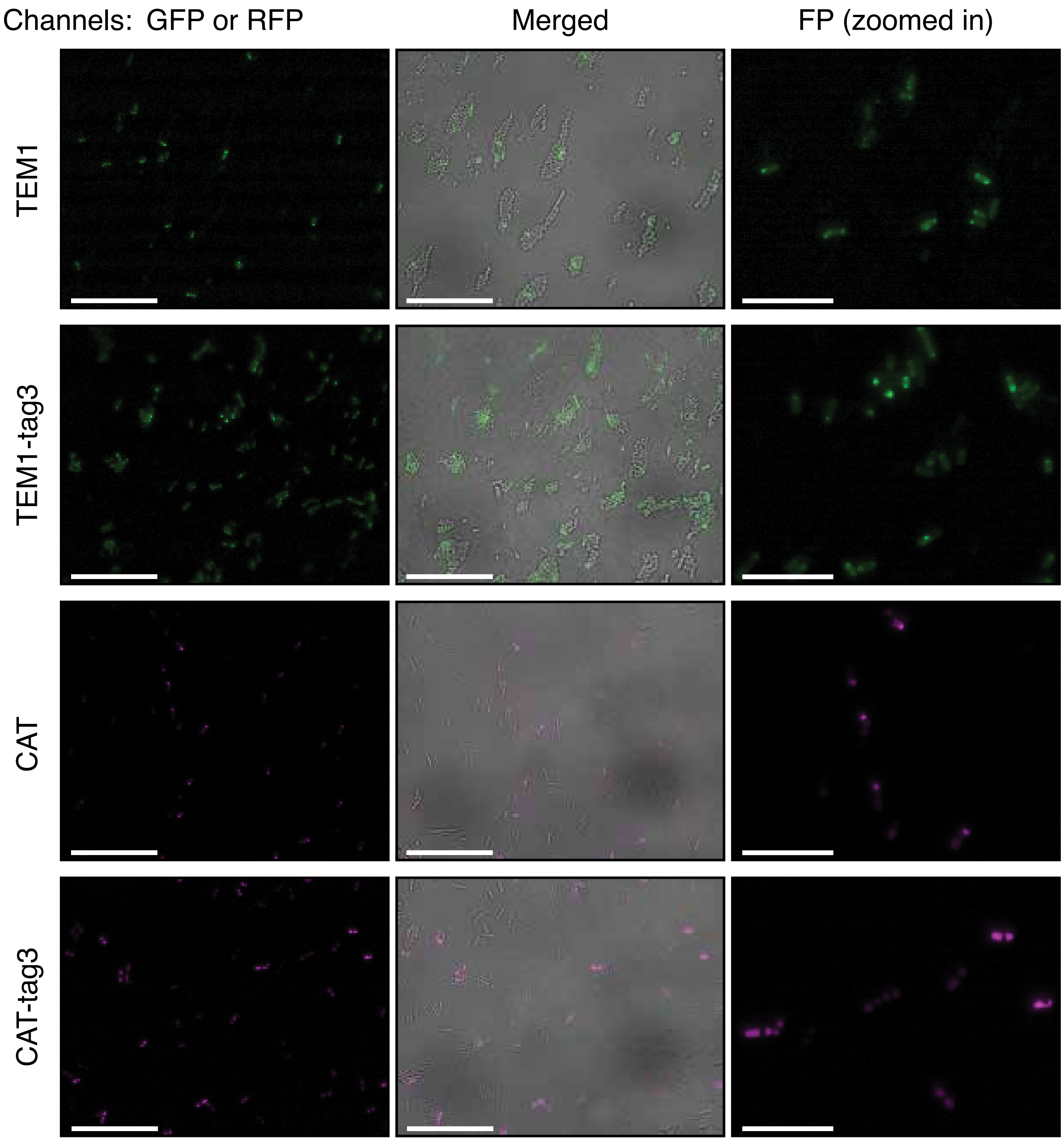

Supplementary Figure 21. Representative microscopy images of *E. coli* cells expressing beta-lactamase (TEM1) or chloramphenicol acetyltransferase (CAT) variants containing an N-terminal TC tag at 24 h post-induction. Beta-lactamase variants were stained with 10 μM FlAsH-EDT$_2$ and images of cell populations were acquired on GFP and brightfield channels. CAT variants were stained with 1 μM ReAsH-EDT$_2$ and images of cell populations were acquired on TexasRed and brightfield channels. Scale bars are 25 μm for GFP or RFP (left) and merged (middle) images, and 10 μm for the zoomed-in (right) images. Microscopy images and analysis were obtained for cells in one biological replicate.

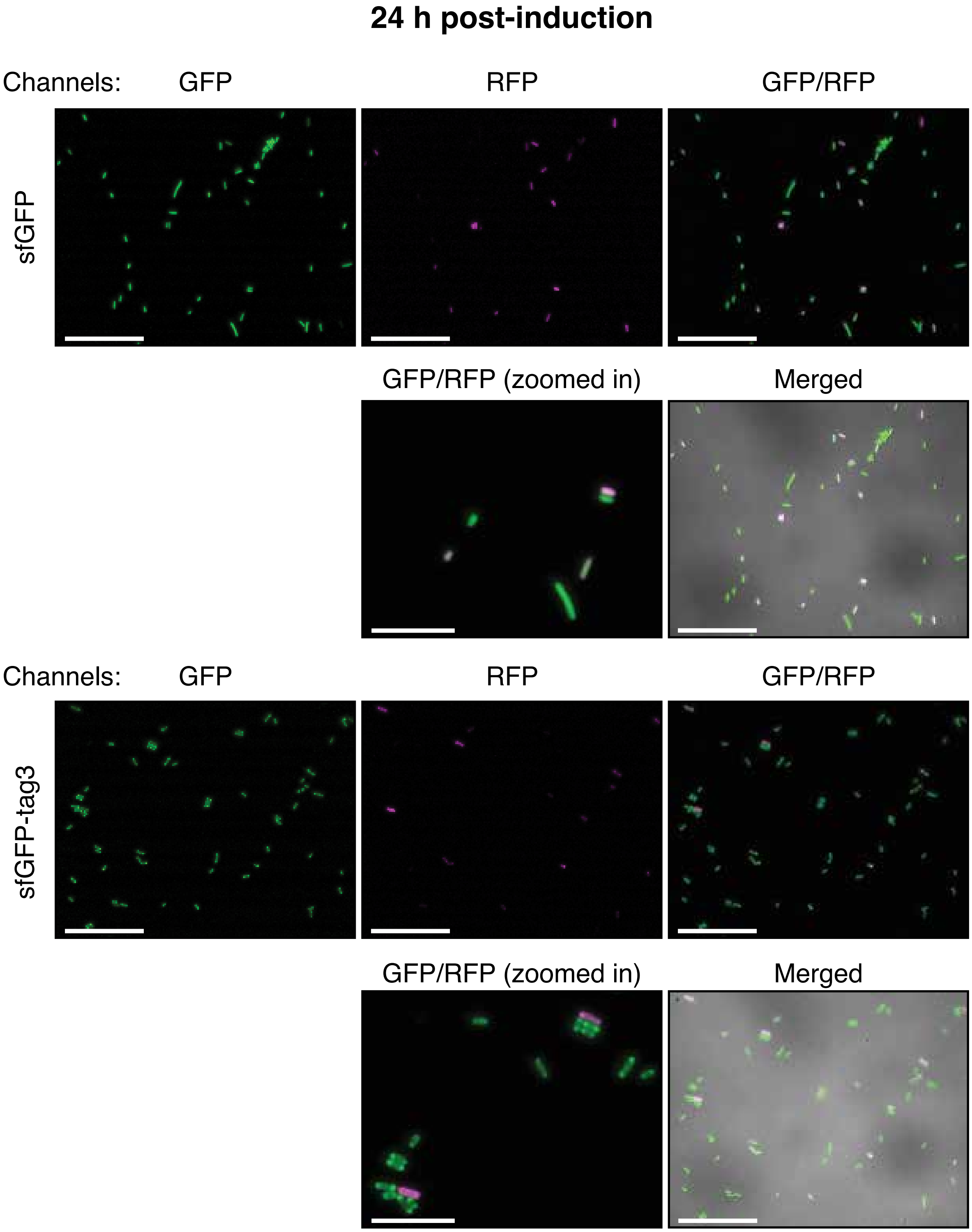

Supplementary Figure 22. Representative microscopy images of *E. coli* cells expressing sfGFP or sfGFP-tag3 containing an N-terminal TC tag at 24 h post-induction. Cells expressing sfGFP and sfGFP-tag3 were stained with 1 μM ReAsH-EDT$_2$ and images of cell populations were acquired on GFP, TexasRed, and brightfield channels. Scale bars are 10 μm for the zoomed-in images and 25 μm for all other images. Microscopy images and analysis were obtained for cells in one biological replicate.

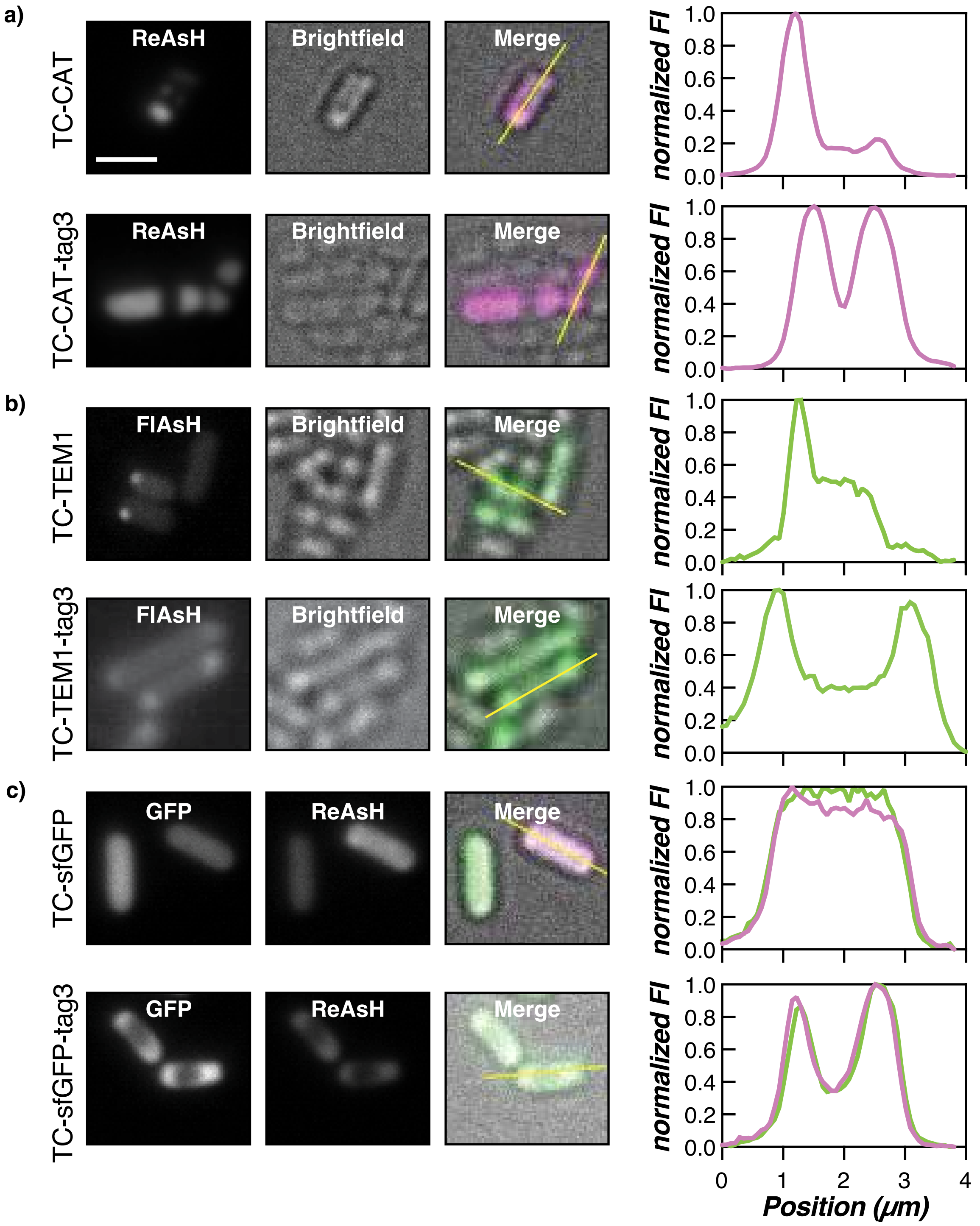

Supplementary Figure 23. ReAsH-EDT$_2$ and FlAsH-EDT$_2$ staining of cells at 24 h post-induction. Image analysis of cells stained with ReAsH (red) or FlAsH (green) for (a) TC-CAT and TC-CAT-tag3, (b) TC-TEM1 and TC-TEM1-tag3, and (c) TC-sfGFP and TC-sfGFP-tag3. Analysis was performed by drawing line cuts across the medial axis of representative cells and plotting the normalized fluorescence intensities in each channel. Scale bar is 2 μm. Two peaks were observed in cells expressing tag3 variants, indicating the localization of the tagged proteins to the cell poles. Unexpectedly, untagged TC-CAT and TC-TEM1 exhibited smaller puncta, which were smaller in size than the tagged variants. ReAsH puncta were also observed in cells expressing TC-sfGFP but only in the RFP channel and not in the GFP channel, suggesting that the puncta observed in untagged cells may be an artifact of the TC tag or arsenic dyes used. Colocalization of ReAsH with sfGFP-tag3 condensates indicates that the arsenic dyes colocalize with TC-tagged proteins. Microscopy images and analysis were obtained for cells in one biological replicate.

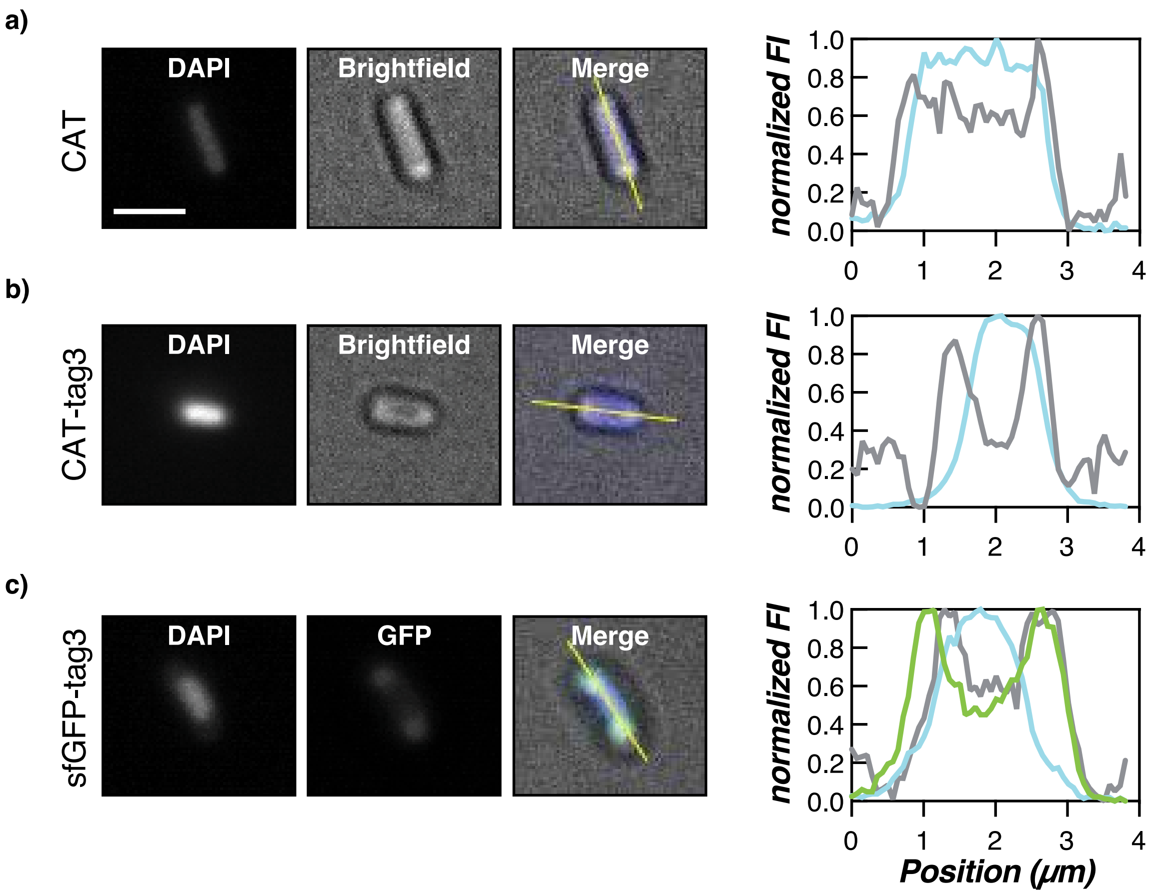

Supplementary Figure 24. DNA localization in cells expressing CAT, CAT-tag3, or sfGFP-tag3 at 24 h post-induction. Cells expressing (a) CAT, (b) CAT-tag3, or (c) sfGFP-tag3 were stained with DAPI at 24 h post-induction to examine the localization of DNA. Line cuts were drawn across representative cells and noramlized fluorescence intensities were plotted at each position along the cell. Scale bar is 2 μm. DAPI and brightfield channels are depicted for non-fluorescent proteins (CAT and CAT-tag3). DAPI, GFP, and merged (DAPI, GFP, brightfield) channels are depicted for sfGFP-tag3. Image analysis revealed that DNA is excluded from sfGFP-tag3 condensates (c). A single peak from the DAPI channel (blue) is found between the two peaks of the GFP (green) and brightfield (grey) channels. Similarly, DNA is excluded from the poles of cells expressing CAT-tag3, which are occupied by CAT-tag3 condensates. In contrast, DNA is distributed throughout cells expressing CAT. Microscopy images and analysis were obtained for cells in one biological replicate.

a)

b)

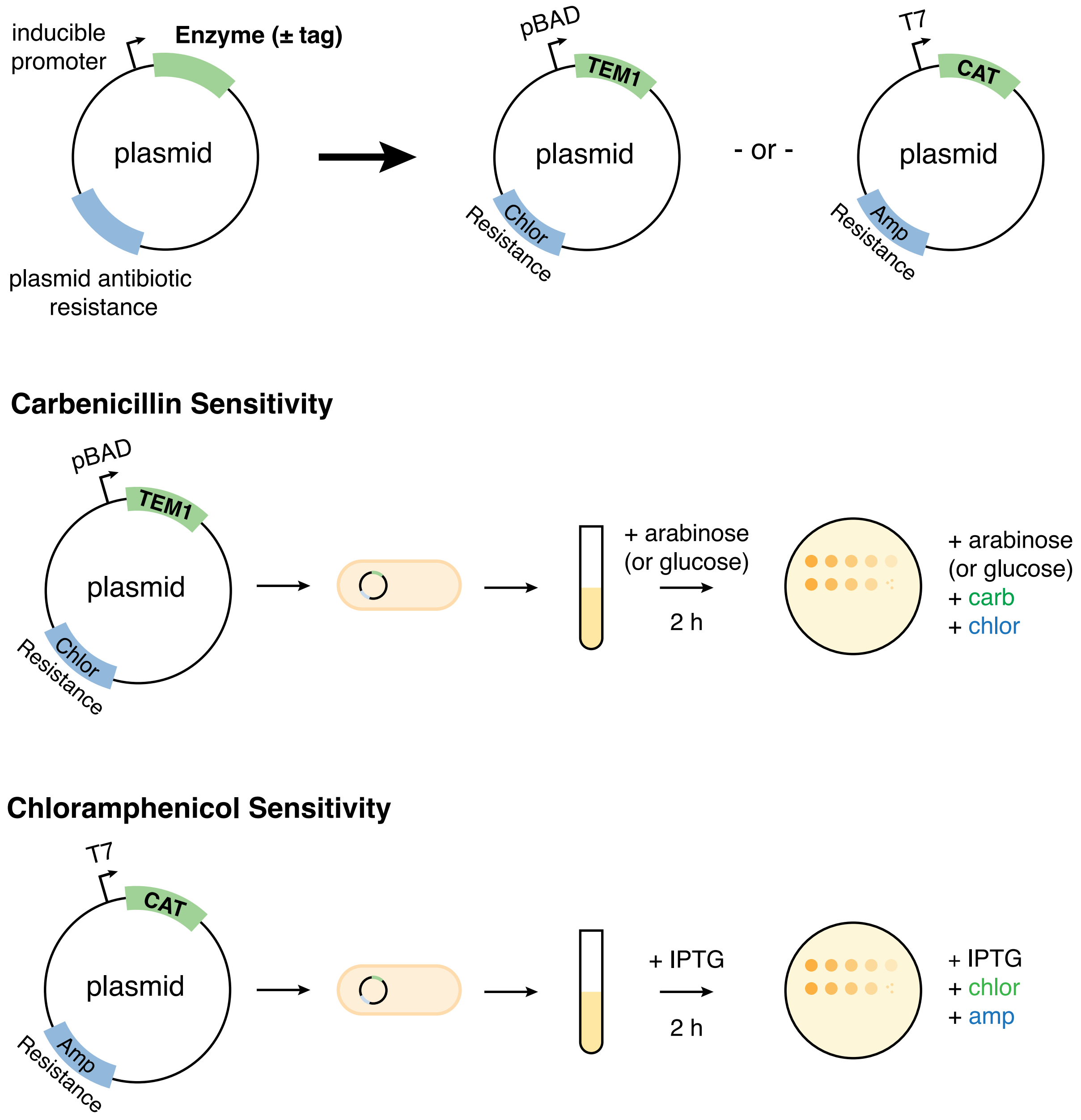

Supplementary Figure 25. Experimental design of carbenicillin and chloramphenicol sensitivity assays. (a) Design of plasmids expressing the beta-lactamase (TEM1) and chloramphenicol acetyltransferase (CAT) variants (in green). TEM1 and CAT expression were induced either by arabinose (pBAD) or IPTG (T7). Plasmids also contained a separate antibiotic resistance marker (in blue) to ensure plasmid retention during the sensitivity assays. (b) Spotting assays were conducted for beta-lactamase (TEM1) and chloramphenicol acetyltransferase (CAT) variants. Briefly, cells from overnight cultures were grown in fresh LB media and induced for 2 h to allow for condensate formation prior to spotting on LB agar plates containing the respective inducers and antibiotics. Plates were supplemented with varying concentrations of carbenicillin or chloramphenicol (in green) to test the sensitivity of the TEM1 or CAT variants, respectively. Plates were also supplemented with a 25 μg/mL chloramphenicol or 100 μg/mL ampicillin for plasmid selection (in blue).

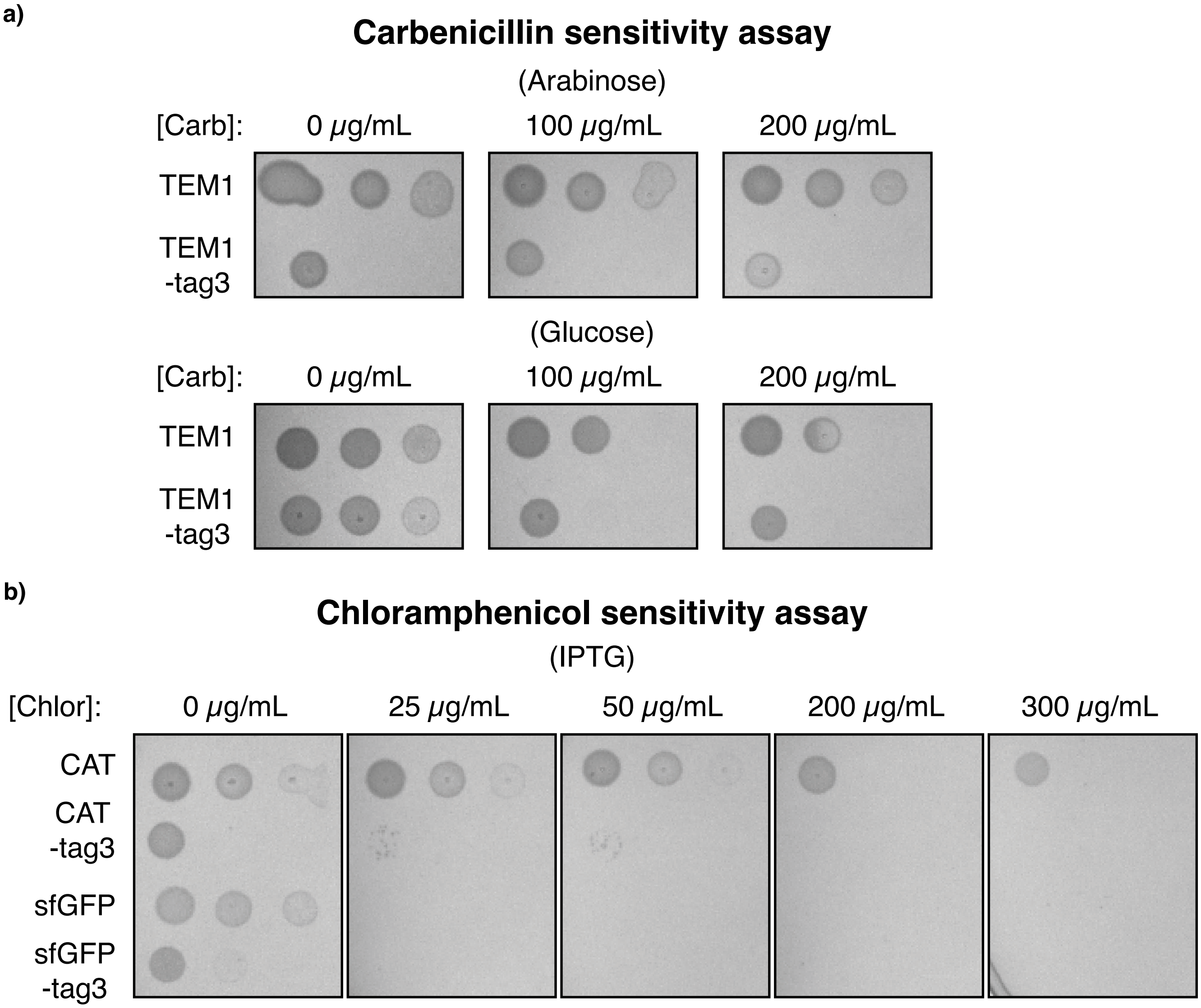

Supplementary Figure 26. Carbenicillin and chloramphenicol sensitivity assays. (a) TEM1 variants were grown on plates supplemented with either arabinose or glucose along with carbenicillin. Starting OD_{600} ~ 1 in the leftmost column, followed by subsequent dilutions (20X and 400X are shown). Reduced growth on the control (no carbenicillin) arabinose plate suggested that condensate formation in the TEM1-tag3 variant reduced cell fitness but did not abolish beta-lactamase activity as these variants could grow on plates supplemented with increasing concentrations of chloramphenicol. Growth of TEM1-tag3 variants on glucose plates showed that TEM1-tag3 maintained enzymatic activity when exposed to carbenicillin despite suppressed expression. Minimal growth differences between TEM1 and TEM1-tag3 were observed in the absence of carbenicillin on glucose plates. (b) CAT variants were grown on plates supplemented with ITPG and chloramphenicol. sfGFP variants were also included as charge-equivalent, negative controls for antibiotic resistance. Starting OD_{600} ~ 0.7 in the leftmost column, followed by subsequent dilutions (100X and 2000X are shown). Similar to the TEM1 variants, reduced fitness without elimination of enzyme activity was observed for tagged CAT variants.

a)

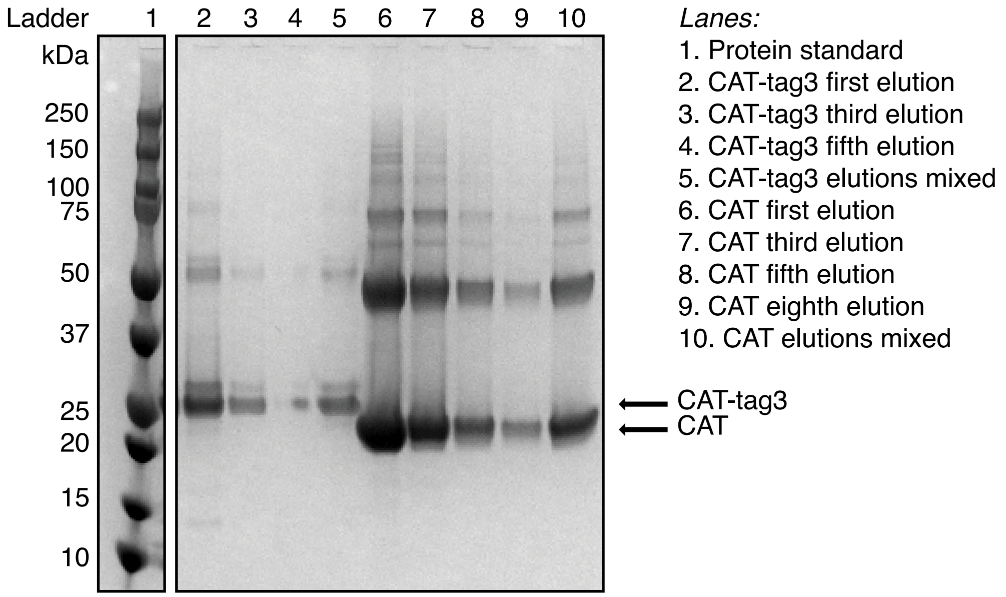

b)

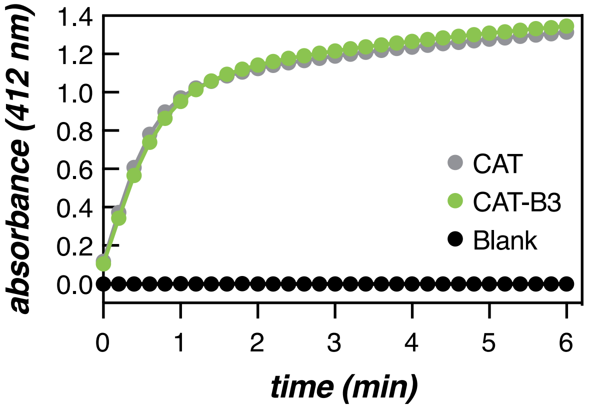

Supplementary Figure 27. Evaluation of CAT activity *in vitro*. (a) Each variant was purified by affinity chromatography and elution fractions were loaded onto a SDS-PAGE gel. Mixed elutions were prepared by mixing equal volumes of the previous elutions (i.e., CAT-tag3 elutions mixed was prepared by mixing equal volumes of the first, third, and fifth elutions). All samples shown here were loaded on the same gel with irrelevant lanes excluded for clarity. We note that CAT is an oligomer and that the higher molecular weight species observed on the SDS-PAGE gel likely correspond to dimers and trimers of CAT.(b) Enzyme activity of 0.25 mg/mL purified CAT variants in 10 mM Tris, 1 mM EDTA, 300 mM NaCl adjusted to pH 7.4. No difference was observed *in vitro* between CAT variants with and without tags. No activity was observed in the control (blank) that only contained buffer. Similar results were observed between three trials for each CAT variant, from the same batch of purified protein.

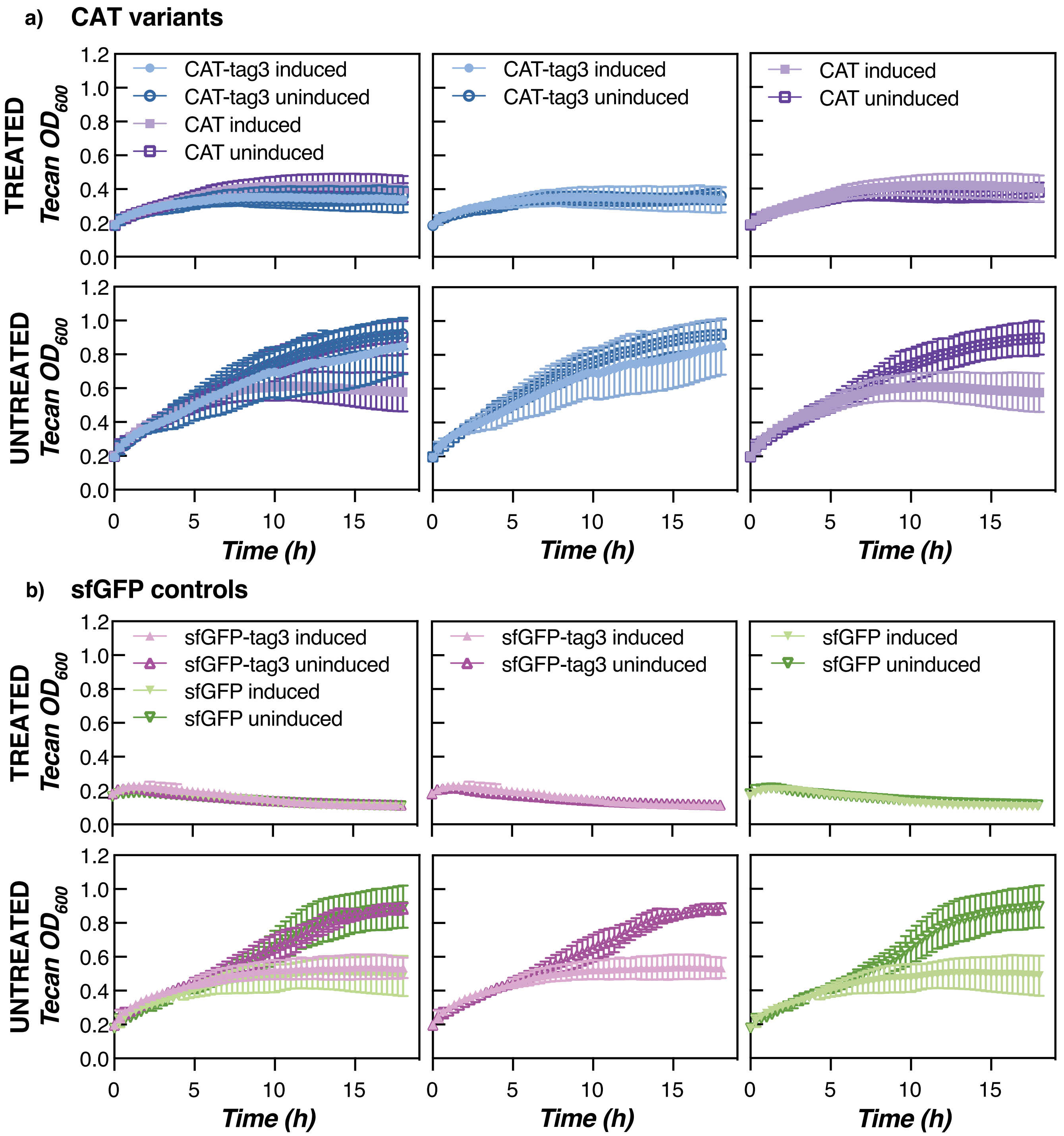

Supplementary Figure 28. Growth of isotropic and tagged CAT variants in liquid media. The growth of (a) CAT and (b) sfGFP variants were monitored in a plate reader in the presence of 200 μg/mL chloramphenicol in ethanol (treated samples). As a control, samples were also grown with the addition of the same volume of ethanol alone (untreated samples). Tecan OD_{600} was monitored for 18 h post-induction in a plate reader. No growth differences were observed between CAT and CAT-tag3 in liquid media treated with chloramphenicol (a). In contrast, uninduced CAT-tag3 appears to grow better than the induced CAT-tag3 in the absence of chloramphenicol. For the sfGFP controls, minimal growth differences were observed between the variants, regardless of induction or treatment. All sfGFP and sfGFP-tag3 samples showed impaired growth throughout the 18 h as these cells are not resistant to chloramphenicol. The average and standard deviation of five biological replicates are plotted for each variant in (a) and (b).

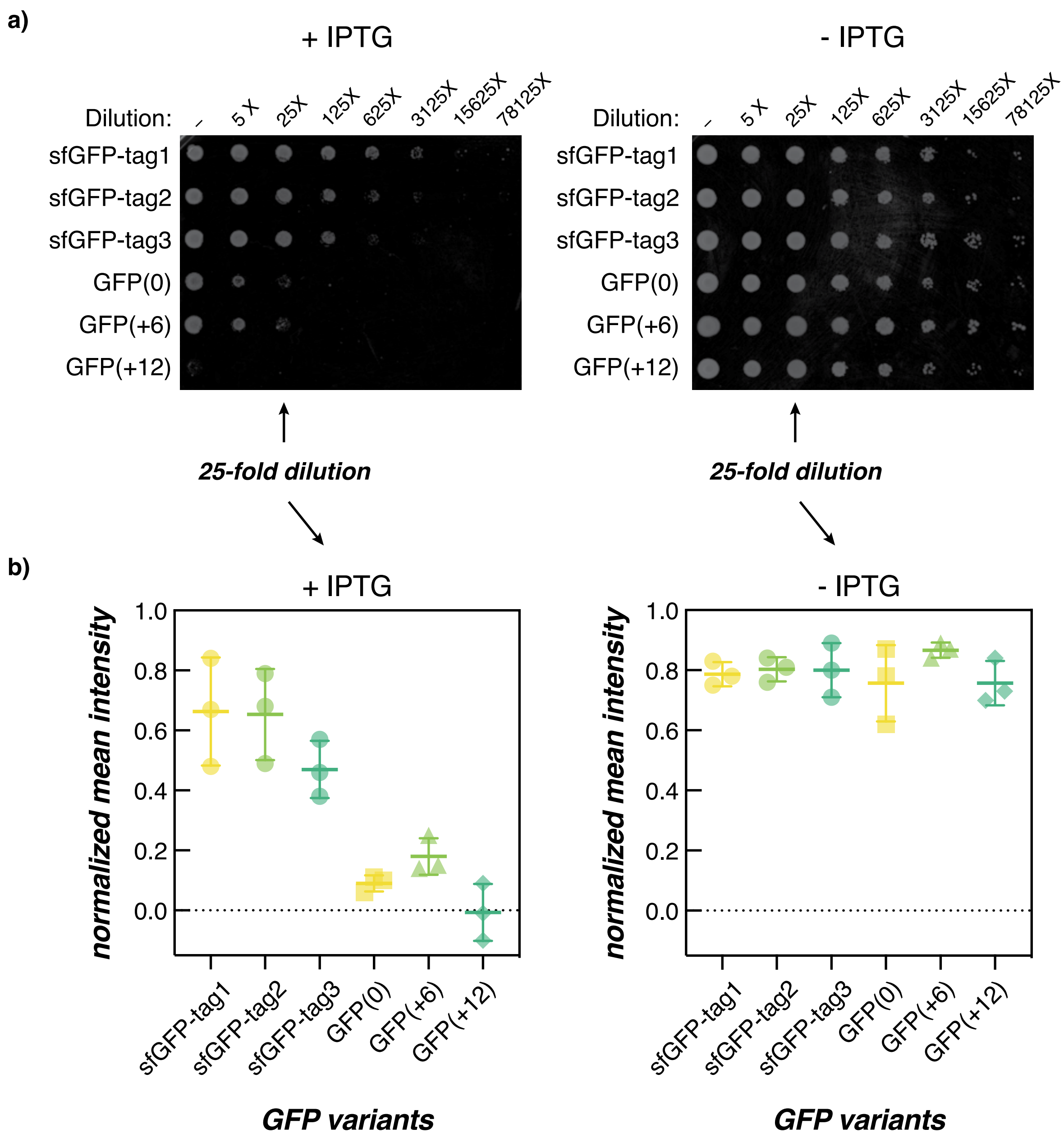

Supplementary Figure 29. Differential growth of isotropic and tagged sfGFP variants on solid media. (a) Representative agar plates depicting growth of cells expressing each sfGFP variant (left). A control was included in which IPTG was not added. One biological replicate is shown on each plate. Briefly, saturated overnight cultures were inoculated in LB media supplemented with 100 μg/mL ampli-cillin and grown to $OD_{600} > 0.9$. All cultures were then normalized to $OD_{600} = 0.9$, and a serial 5-fold dilution was performed in LB media supplemented with 100 μg/mL amplicillin before plating onto LB agar in the presence or absence of 1 mM IPTG using a 96-pin microplate replicator. After drying, plates were grown at 25 °C for ~30 h and scanned. (b) Analysis of samples diluted 25-fold. Briefly, the mean intensity of spotted cells was normalized to that of the undiluted sample for each GFP variant and plotted as the normalized mean intensity. Tagged GFP variants grow better on solid media than the isotropic equivalents. The average and standard deviation of three biological replicates are plotted for each variant.

Supplementary Note

```
% This MATLAB script identifies condensates following image
preprocessing by ImageJ and cell identification by MicrobeJ.
% This script averages nine central pixels within each cell image to
determine an approximate average pixel intensity for
% non-condensate areas of the cell. For each cell, all pixels 20%
% higher in intensity than its approximate average intensity were
identified as condensates. 20%
% higher intensity was selected by empirical evaluation of what best
distinguished cells that had been visually identified as
% condensate-containing from cells visually identified as non-
condensate containing.
% A duplicate image with all non-condensate pixels set to zero was
then created for each cell.
% The number of condensate-containing cells was counted for each
experiment
% and divided by the total number of cells to determine the fraction
of condensate-containing cells identified for each experiment.

% get all files of the .MAT type in the current folder, save the names
fds = fileDatastore('*.MAT', 'ReadFcn', @importdata);
fullFileNames = fds.Files;

% initialize variables for the loop below
partialNames = string(fullFileNames);
numFiles = length(fullFileNames);
cells = {};
average = {};
norm_average = {};
condensates = {};
size_condensate = {};
counter = 0;
num_cells = zeros(1,numFiles);
k = 0;
i = 0;

% Take each experiment, get all of the cells in each and store them as
an
% array of doubles. Also trim the file path off of the names for data
% exporting
for k = 1 : numFiles
    partialNames(k) = char(erase(fullFileNames(k),"O:
\Inventory\Temporary Transfer\Vivian\PROTHYMOSIN ALPHA
COLLABORATION\"));
    temp_cells = load(partialNames(k));
    num_cells(k) = length(temp_cells.Experiment.Bacteria);

    % storing each cell in the cells array
    for i = 1 : num_cells(k)
        temp_pixels = temp_cells.Experiment.Bacteria(i).PROFILE_MED;
        cells{k,i} = temp_pixels.ch.pixel;
```

```
    end
end

% initializing variables
per_non_zero = zeros(1,numFiles);
threshold = zeros(1,9);

% threshold all of the cells, store the thresholded cells. Record the
% percent of non-zero cells for each cell line at the given threshold
value
for k = 1 : numFiles
    num_non_zero = 0;
    temp_num_cells = 0;
    temp_frac_condenate = [];
    temp_size_cells = [];
    temp_size_condensate = [];

    % Loop to vary thresholds to determine which threshold will best
    % distinguish cells with condensates from cells without
condensates.
    % Currently hard-coded to 120% of the averaged central pixles
    for j = 1 : 1

        % loop through every cell in a given file. Determine each
cell's
        % threshold and the number of pixels that exceed the threshold
        for i = 1 : num_cells(k)

            % initialize temporary variables for pixels
            temp_pixels = cells{k,i};
            temp_average = mean(temp_pixels,'all');

            % Determine the central pixel of the cell. Determine the
            % threshold by averaging the 9 pixels in the center of the
cell
            % and multiplying by a threshold factor
            x_center = round(size(temp_pixels,1)/2);
            y_center = round(size(temp_pixels,2)/2);
            threshold(1) = temp_pixels(x_center,y_center) *
(1.05+0.05*(j+2));
            threshold(2) = temp_pixels(x_center+1,y_center) *
(1.05+0.05*(j+2));
            threshold(3) = temp_pixels(x_center,y_center+1) *
(1.05+0.05*(j+2));
            threshold(4) = temp_pixels(x_center+1,y_center+1) *
(1.05+0.05*(j+2));
            threshold(5) = temp_pixels(x_center-1,y_center-1) *
(1.05+0.05*(j+2));
            threshold(6) = temp_pixels(x_center-1,y_center) *
(1.05+0.05*(j+2));
```

```
            threshold(7) = temp_pixels(x_center,y_center-1) *
(1.05+0.05*(j+2));
            threshold(8) = temp_pixels(x_center-1,y_center+1) *
(1.05+0.05*(j+2));
            threshold(9) = temp_pixels(x_center+1,y_center-1) *
(1.05+0.05*(j+2));
            avg_threshold = mean(threshold);

            % set all pixels in temp_pixels under the average equal to
0
            temp_pixels(temp_pixels < avg_threshold) = 0;

            % store the condensates images, store the number of
condensate
            % pixels and number of total pixels
            condensates{k,i} = temp_pixels;
            size_condensate{k,i} = nnz(temp_pixels);
            temp_size_condensate(i) = nnz(temp_pixels);
            temp_size_cells(i) = numel(temp_pixels);
            size_cells{k,i} = numel(temp_pixels);



            % if there are any non-zero pixels in the condensate
image,
            % increase the number of non-zero cells
            if size_condensate{k,i} ~= 0
                num_non_zero = num_non_zero+1;
            end
        end


        % Determine the percentage of cells that have some area that
has
        % been identified as a condensate. Store in array for data
        % validation purposes, checking the
        per_non_zero(j,k) = num_non_zero / num_cells(k);
        num_non_zero = 0;

        % Put the fraction of each cell identified as condensate in an
array
        % for data validation purposes
        temp_frac_condensate = temp_size_condensate ./ temp_size_cells;
        for j = 1:length(temp_frac_condensate);
            frac_condensate{j,k} = temp_frac_condensate(j);
        end
    end
end
```

Supplementary References

1. Kapelner, R. A. & Obermeyer, A. C. Ionic polypeptide tags for protein phase separation. *Chem. Sci.* **10**, 2700–2707 (2019).
2. Lamiable, A. *et al.* PEP-FOLD3: faster *de novo* structure prediction for linear peptides in solution and in complex. *Nucleic Acids Res* **44**, W449–W454 (2016).
3. Shen, Y., Maupetit, J., Derreumaux, P. & Tufféry, P. Improved PEP-FOLD Approach for Peptide and Mini-protein Structure Prediction. *J. Chem. Theory Comput.* **10**, 4745–4758 (2014).
4. Thevenet, P. *et al.* PEP-FOLD: an updated de novo structure prediction server for both linear and disulfide bonded cyclic peptides. *Nucleic Acids Research* **40**, W288–W293 (2012).
5. Yeong, V., Werth, E. G., Brown, L. M. & Obermeyer, A. C. Formation of Biomolecular Condensates in Bacteria by Tuning Protein Electrostatics. *ACS Cent. Sci.* acscentsci.0c01146 (2020) doi:10.1021/acscentsci.0c01146.
