# Supplementary material for: Intracellular phase separation of globular proteins facilitated by short cationic peptides
Source: Nat Commun. 2022 Dec 22;13:7882. doi: 10.1038/s41467-022-35529-2 (PMC9780332; doi:10.1038/s41467-022-35529-2)
Supplement: Supplementary file 2 — Reporting Summary [file 41467_2022_35529_MOESM2_ESM.pdf]

nature portfolio

Corresponding author(s): Obermeyer, A. C.
Last updated by author(s): 11/29/2022

Reporting Summary

Nature Portfolio wishes to improve the reproducibility of the work that we publish. This form provides structure for consistency and transparency in reporting. For further information on Nature Portfolio policies, see our Editorial Policies and the Editorial Policy Checklist.

Statistics

For all statistical analyses, confirm that the following items are present in the figure legend, table legend, main text, or Methods section.

n/a	Confirmed	
☐	☒	The exact sample size (*n*) for each experimental group/condition, given as a discrete number and unit of measurement
☐	☒	A statement on whether measurements were taken from distinct samples or whether the same sample was measured repeatedly
☒	☐	The statistical test(s) used AND whether they are one- or two-sided<br>*Only common tests should be described solely by name; describe more complex techniques in the Methods section.*
☒	☐	A description of all covariates tested
☒	☐	A description of any assumptions or corrections, such as tests of normality and adjustment for multiple comparisons
☐	☒	A full description of the statistical parameters including central tendency (e.g. means) or other basic estimates (e.g. regression coefficient) AND variation (e.g. standard deviation) or associated estimates of uncertainty (e.g. confidence intervals)
☒	☐	For null hypothesis testing, the test statistic (e.g. *F*, *t*, *r*) with confidence intervals, effect sizes, degrees of freedom and *P* value noted<br>*Give P values as exact values whenever suitable.*
☒	☐	For Bayesian analysis, information on the choice of priors and Markov chain Monte Carlo settings
☒	☐	For hierarchical and complex designs, identification of the appropriate level for tests and full reporting of outcomes
☒	☐	Estimates of effect sizes (e.g. Cohen's *d*, Pearson's *r*), indicating how they were calculated

Our web collection on statistics for biologists contains articles on many of the points above.

Software and code

Policy information about availability of computer code

Data collection	No software used for data collection.
Data analysis	The following software was used for data analysis: GraphPad Prism (v. 9.1.0), microbeJ (v. 15.3l (14)), MATLAB (v. R2020b), PyMol (v. 2.4.1), and PEP-FOLD3. Code used to analyze condensates in cells was written in MATLAB and is included in the SI.

For manuscripts utilizing custom algorithms or software that are central to the research but not yet described in published literature, software must be made available to editors and reviewers. We strongly encourage code deposition in a community repository (e.g. GitHub). See the Nature Portfolio guidelines for submitting code & software for further information.

Data

Policy information about availability of data

All manuscripts must include a data availability statement. This statement should provide the following information, where applicable:

- Accession codes, unique identifiers, or web links for publicly available datasets
- A description of any restrictions on data availability
- For clinical datasets or third party data, please ensure that the statement adheres to our policy

The following data from public repositories was used herein: superfolder GFP crystal structure, PDB ID: 2B3P, and E. coli proteome information, UniProt: UP000002032. Raw uncropped microscopy and gel images are available via figshare http://dx.doi.org/10.6084/m9.figshare.21568371. All other data are available from the corresponding author upon request.

Human research participants

Policy information about studies involving human research participants and Sex and Gender in Research.

Reporting on sex and gender	not applicable
Population characteristics	not applicable
Recruitment	not applicable
Ethics oversight	not applicable

Note that full information on the approval of the study protocol must also be provided in the manuscript.

Field-specific reporting

Please select the one below that is the best fit for your research. If you are not sure, read the appropriate sections before making your selection.

- [x] Life sciences
- [ ] Behavioural & social sciences
- [ ] Ecological, evolutionary & environmental sciences

For a reference copy of the document with all sections, see nature.com/documents/nr-reporting-summary-flat.pdf

Life sciences study design

All studies must disclose on these points even when the disclosure is negative.

Sample size	No sample size calculation was performed to determine the necessary sample size. In each experiment, we captured at least three independent biological replicates in the form of separate measurements with multiple (10-100s) of cells monitored in each replicate. The analysis of this number of cells and biological replicates was sufficient to make qualitative comparisons between various engineered proteins and whether or not they formed cellular condensates.
Data exclusions	No data were excluded.
Replication	Experiments were repeatable with the three independent measurements that were performed. All attempts at replication were successful. There was some expected variation between biological replicates but none that altered the findings or claims in the manuscript.
Randomization	This is not relevant to our study because each engineered strain was an independent to be analyzed.
Blinding	At least one of the investigators was blinded for the manual identification of whether or not condensates were present in the strains expressing the various engineered proteins.

Reporting for specific materials, systems and methods

We require information from authors about some types of materials, experimental systems and methods used in many studies. Here, indicate whether each material, system or method listed is relevant to your study. If you are not sure if a list item applies to your research, read the appropriate section before selecting a response.

Materials & experimental systems

n/a	Involved in the study	
☒	☐	Antibodies
☒	☐	Eukaryotic cell lines
☒	☐	Palaeontology and archaeology
☒	☐	Animals and other organisms
☒	☐	Clinical data
☒	☐	Dual use research of concern

Methods

n/a	Involved in the study	
☒	☐	ChIP-seq
☒	☐	Flow cytometry
☒	☐	MRI-based neuroimaging
